# High‐dimensional spectral flow cytometry uncovers progressive T‐cell exhaustion and myeloid cell reprogramming in a CLL mouse model

**DOI:** 10.1002/hem3.70476

**Published:** 2026-09-14

**Authors:** Hannah Briesch, Mariana Coelho, Alessia Floerchinger, Yuliia Borysovych, Nicolas Aubert, Daniel Medina‐Gil, Manina Breitinger, Marc Zapatka, Stella E. Autenrieth, Martina Seiffert

**Affiliations:** ^1^ Immune Modulation in Cancer German Cancer Research Center (DKFZ) Heidelberg Germany; ^2^ Division of Molecular Genetics German Cancer Research Center (DKFZ) Heidelberg Germany; ^3^ Faculty of Biosciences Heidelberg University Heidelberg Germany; ^4^ Dendritic Cells in Infection and Cancer German Cancer Research Center (DKFZ) Heidelberg Germany

## Abstract

Chronic lymphocytic leukemia (CLL) exhibits marked clinical heterogeneity that cannot be fully explained by genetic alterations, underscoring the critical role of the tumor microenvironment in disease progression and therapeutic resistance. To delineate the temporal evolution of immune dysregulation in CLL, we used the Eµ‐TCL1 adoptive transfer (TCL1‐AT) mouse model combined with high‐dimensional spectral flow cytometry to longitudinally profile lymphoid and myeloid compartments. To assess the reproducibility across different disease kinetics, we analyzed seven independent TCL1‐AT cohorts generated from distinct primary tumors. CLL progression was associated with coordinated restructuring of adaptive and innate immune cell populations. Within the T‐cell compartment, early activation and effector expansion transitioned to a progressive accumulation of terminally exhausted CD8^+^ T cells. Concurrently, immunoregulatory populations expanded, including regulatory type 1 (Tr1)‐like CD4^+^ T cells, activated regulatory T cells (Tregs) and, importantly, a previously underappreciated Eomes‐expressing Treg subset. Natural killer (NK) cells exhibited transient expansion followed by differentiation and acquisition of exhaustion markers. Myeloid cell profiling revealed an expansion of immunosuppressive PD‐L1^high^ patrolling monocytes, mature regulatory dendritic cells (mregDCs), and phenotypic reprogramming of dendritic cells (DCs) consistent with impaired antigen presentation and promotion of T‐cell exhaustion and regulatory T‐cell subsets. Despite substantial variation in leukemia kinetics, expansion of patrolling monocytes, exhausted CD8^+^ T cells, and Tregs was consistently observed across all seven independent TCL1‐AT cohorts, demonstrating robust immune remodeling. Collectively, our findings demonstrate a stepwise transition toward a suppressive microenvironment involving innate and adaptive immune compartments in the Eµ‐TCL1 mouse model, providing a framework for understanding immune failure in CLL and informing strategies to restore anti‐leukemic immunity.

## Graphical abstract

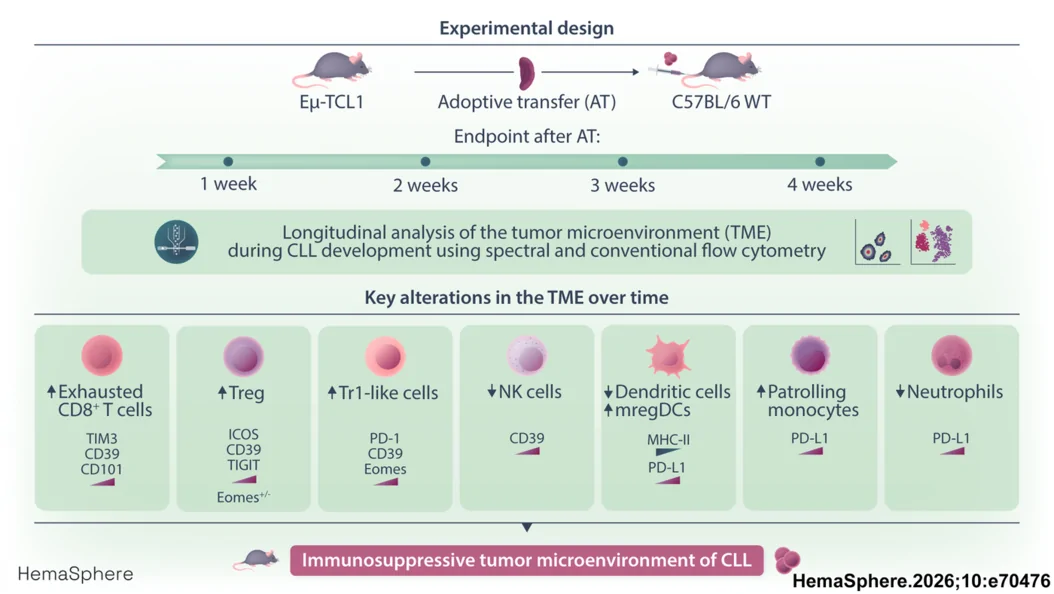

## INTRODUCTION

Chronic lymphocytic leukemia (CLL) is characterized by profound clinical heterogeneity: while some patients experience indolent disease for many years, others progress rapidly or develop resistance to targeted therapies despite sharing similar genetic features.^1^ This disconnect between genetic alterations and clinical behavior underscores the need to examine additional layers of disease biology, especially the tumor microenvironment (TME), which plays a central role in shaping leukemic cell survival, immune evasion, and treatment response.^2^ Increasing evidence suggests that CLL cannot be fully understood solely through malignant cells; instead, its course is profoundly influenced by dynamic interactions between leukemic cells and the surrounding immune ecosystem.^3^ Strong evidence comes from a recent report showing that malignant “seed” cells associated with the aggressive transformation of CLL into Richter's syndrome were detectable years before the transformative event, indicating that genomic aberrations were not the only drivers; extrinsic factors also contribute to disease control and escape in CLL.^4^

This need has become even more pressing in the era of immunotherapies. Despite transformative success in other hematologic malignancies, approaches such as immune checkpoint blockade and CAR T‐cell therapy have shown limited efficacy in CLL.^5^, ^6^ One emerging explanation is that CLL creates a uniquely dysfunctional TME, marked by chronic antigenic stimulation, impaired T‐cell fitness, and myeloid‐driven immune suppression, conditions that blunt therapeutic immune activation.^7^ Although we and others have described individual components of the CLL microenvironment,^8^, ^9^, ^10^ the temporal evolution of the TME as leukemia progresses remains poorly understood. Understanding *how* immune dysregulation emerges and deepens over time may reveal why some hosts maintain durable immune control while others succumb to progressive disease or therapy resistance.

To address this gap, there is a growing emphasis on high‐dimensional approaches that capture the complexity of the immune system. Using mass cytometry (CyTOF), Wierz et al. revealed substantial immune heterogeneity and complex cellular interactions within the CLL microenvironment in the Eµ‐TCL1 mouse model of CLL, highlighting the coexistence of multiple immunoregulatory cell populations.^11^ Likewise, single‐cell RNA sequencing analyses of peripheral blood (PB) and lymph node (LN) samples from CLL patients demonstrated that the immune microenvironment shapes both transcriptional programs and clonal evolution of leukemic cells, underscoring the dynamic interplay between malignant and immune compartments.^12^ Our recent observations, combining single‐cell RNA sequencing, mass cytometry, and spatial profiling of PB and LN samples from patients with CLL and other B‐cell lymphomas, identified pronounced enrichment of activated and exhausted T‐cell populations within malignant LNs, underscoring the importance of the lymphoid microenvironment as a site of progressive immune dysfunction and immune escape.^8^, ^13^ However, these studies were largely cross‐sectional and therefore could not resolve how immune dysfunction emerges and evolves over time during disease progression.

High‐dimensional spectral flow cytometry (spectral FC) enables simultaneous measurement of multiple immune markers at single‐cell resolution while preserving the throughput required to study longitudinal disease evolution. Spectral FC precisely delineates adaptive and innate immune populations, including activated and exhausted T‐cell subsets, neutrophils, monocytes, and suppressive myeloid cells, offering an unprecedented view of the CLL TME's structure and dynamics. It is ideal for studying how immune networks change over time in vivo and for identifying coordinated lineage shifts that may drive or reflect leukemia progression.

The Eµ‐TCL1 AT mouse model, which recapitulates key immunologic and leukemic features of human CLL, offers an ideal system for high‐resolution immune profiling over the course of CLL development.^14^ Using this model, we observed a stepwise remodeling of the immune microenvironment: T cells become progressively activated yet increasingly exhausted, while the myeloid compartment undergoes substantial restructuring. Early neutrophil expansion is followed by the accumulation of immunosuppressive monocytes, suggesting a gradual shift toward a myeloid‐dominated suppressive niche that undermines adaptive immune surveillance. Mapping these trajectories with spectral FC provides mechanistic insight into how CLL shapes, and is shaped by, its microenvironment across disease stages.

By delineating the evolving states of adaptive and innate immune populations during CLL development, our study aims to clarify how immune dysfunction arises, how it contributes to leukemic progression, and why current immunotherapies fail to achieve robust responses in CLL. Understanding these processes is essential for designing strategies that restore immune competence and improve therapeutic outcomes for patients with CLL.

## MATERIAL AND METHODS

### Mice and tumor models

Female C57BL/6 wild‐type (WT) mice were purchased from Janvier Labs (France). Adoptive transfer (AT) of malignant B cells from Eμ‐TCL1 mice was conducted as described previously.^8^, ^15^ In brief, malignant B cells were enriched from cryopreserved splenocytes of Eµ‐TCL1 mice^16^ using EasySep Mouse Pan‐B Cell Isolation Kit (StemCell Technologies) according to the manufacturer's instructions. The purity of CD5^+^CD19^+^ CLL cells, which was usually above 95% of purified cells, was confirmed by flow cytometry. For AT into C57BL/6J recipient mice, 0.5–1.5 × 10^7^ malignant TCL1 splenocytes were transplanted by intravenous (i.v.) injection into 7–10‐week‐old C57BL/6 WT female mice. Disease progression was monitored via daily assessment of the mice and via CLL load analysis in PB by weekly blood withdrawal from facial vein into ethylenediaminetetraacetic acid (EDTA)‐coated tubes (Sarstedt), followed by flow cytometry analysis.

### Tissue sample collection and processing

Mice were sacrificed with increasing concentrations of carbon dioxide (CO_2_) before reaching the established endpoint criteria. Blood was drawn via cardiac puncture and collected into EDTA‐coated tubes, and single‐cell suspensions were prepared from spleen, bone marrow (BM), and inguinal LNs. Single‐cell suspensions of spleens were obtained via mechanical disruption in 9 mL medium in Gentle MACS tubes C using gentleMACS tissue dissociator (Miltenyi Biotec, program 4.01 for 1 min followed by program 1.01 for 1 min). After red blood cell lysis in RBC lysis buffer (BioLegend) for 10 min, cells were filtered through 70‐μm cell strainers. BM cells were flushed from femurs with 5 mL phosphate‐buffered saline (PBS), 2% fetal calf serum and filtered through 40 μm cell strainers. Inguinal LNs were mechanically dissociated by grinding the tissue through 70‐µm cell strainers (BD Biosciences). Total viable cell counts of splenocytes and BM cells were obtained using Vi‐Cell XR cell viability analyzer (Beckman Coulter). Afterwards, cells were stained or cryopreserved for later analysis. To deplete CLL cells, the EasySep Mouse CD19 Positive Selection Kit II (StemCell) was used according to the manufacturer's instructions.

### Seven independent TCL1 adoptive transfer experiments

To investigate changes in the TME while accounting for intertumoral heterogeneity, seven independent TCL1 adoptive transfer (TCL1‐AT) experiments were performed. Splenic B cells derived from seven individual tumors (T1–T7) were adoptively transferred into 10‐week‐old female C57BL/6 J mice (*n* = 5 per tumor, except T6 with *n* = 9). Age‐matched untreated or PBS‐injected C57BL/6J mice served as controls. CLL burden and immune cell populations in PB were assessed by conventional flow cytometry one day prior to AT and subsequently at weekly intervals until mice reached endpoint criteria and were euthanized. At endpoint, spleen, inguinal LNs, and BM were collected for further analysis. The exact origin of the tumors, as well as time points of blood withdrawals and euthanasia for each of the seven TCL1‐AT experiments are provided in Supporting Information S1: Table 1.

### Longitudinal TCL1 adoptive transfer model

To assess immune dynamics during CLL progression in mice, four groups of 7‐week‐old, female C57BL/6J mice (*n* = 9 per time point) received a single i.v. injection of cryopreserved, B‐cell–enriched splenocytes of Eµ‐TCL1 mice at 1‐week intervals for four consecutive weeks, as described above. One control mouse per week received sterile PBS. All mice were euthanized on the same day, with cohorts representing sequential stages of tumor development. Blood, spleen, LNs, and BM were collected as described above for flow cytometric analysis of immune cell subsets and for characterization of the splenic TME by spectral FC.

### Conventional and spectral flow cytometry

A detailed description of staining and acquisition of samples, and analysis of conventional and spectral FC data is provided in Supporting Information S2.

## RESULTS

### Longitudinal analysis of blood samples from TCL1 adoptive transfer experiments reveals consistent immune remodeling during CLL development

**Figure 1:**
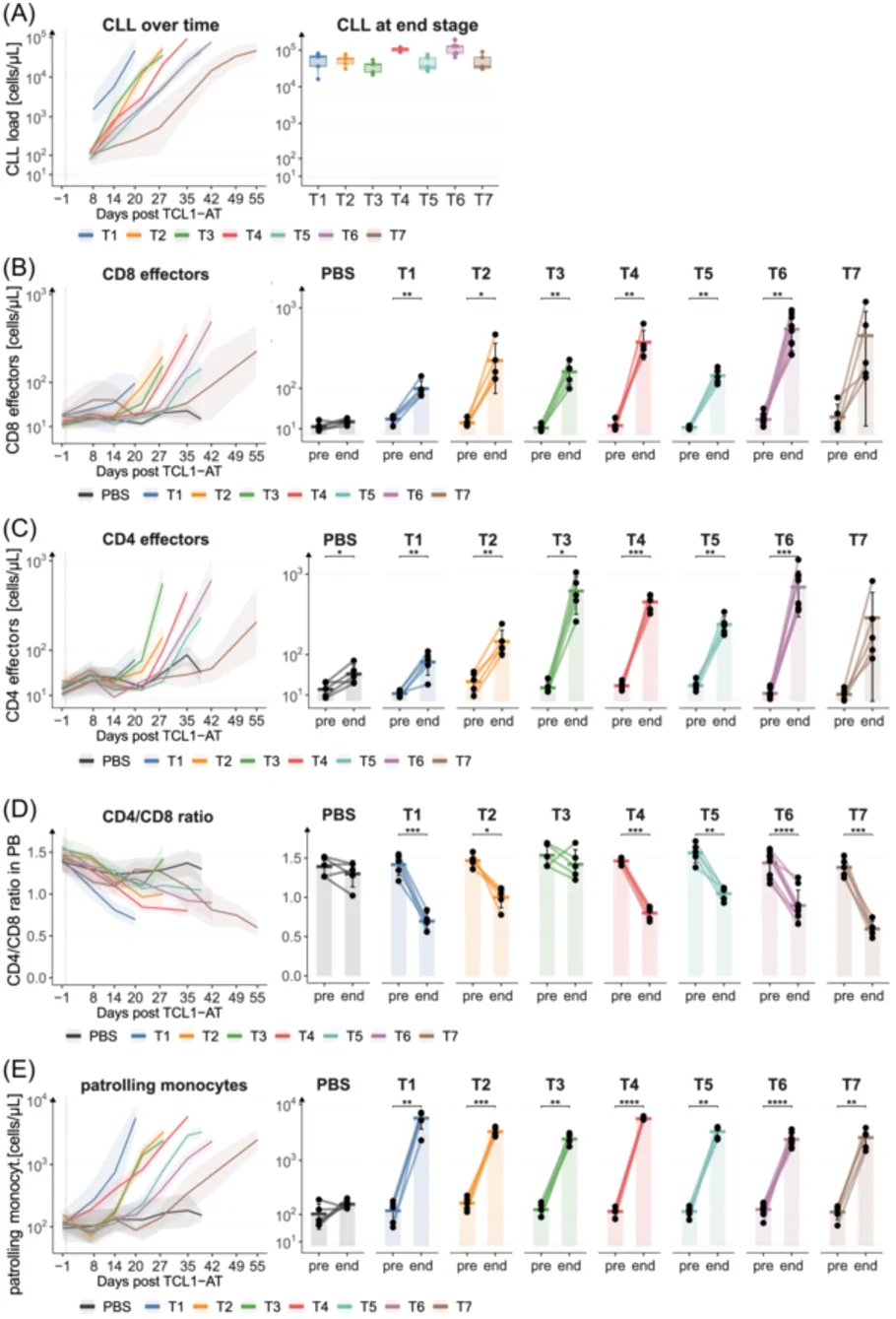
**Longitudinal analysis of independent TCL1 adoptive transfer (TCL1‐AT) experiments shows reproducible immune remodeling during chronic lymphocytic leukemia (CLL) development.** Seven independent TCL1 donor tumors (T1–T7) were transplanted into C57BL/6J recipient mice (*n* = 5 per tumor, except T6 with *n* = 9); phosphate‐buffered saline (PBS)‐injected mice were used as control. CLL burden and immune cell populations in peripheral blood were analyzed 1 day before AT (Day −1) and during weekly blood withdrawals until mice were euthanized upon reaching endpoint criteria. The exact time points of blood withdrawals and euthanasia for each of the seven TCL1‐AT experiments are provided in Supporting Information S1: Table 1. **(A–E)** Conventional flow cytometry‐based analysis of CLL, T, and myeloid cells in peripheral blood after TCL1‐AT. Absolute counts of CLL cells (A), CD8^+^ effector T cells (B), CD4^+^ effector T cells (C), as well as the CD4/CD8 ratio (D), and counts of patrolling monocytes (E) in peripheral blood (PB) were quantified over time (left panels). Lines represent the mean, and shaded areas indicate the standard deviation (SD). Right panels show paired comparisons between measurements obtained 1 day before AT (pre) and the last blood withdrawal performed 0–5 days before euthanasia upon reaching endpoint criteria (end, exact days provided in Supporting Information S1: Table 1). Bar plots indicate the mean with SD; paired measurements within the same mice are indicated by a connected line. Statistical significance between 1 day before AT and the last time point was assessed within each group using paired two‐tailed Student's *t*‐tests with Bonferroni–Holm correction for multiple testing. ****P < 0.0001, ***P ≤ 0.001, **P ≤ 0.01, and *P ≤ 0.05.

To investigate the dynamics of immune remodeling during CLL development, we employed the TCL1‐AT model and longitudinally monitored CLL and immune cell counts in PB by flow cytometry. Using seven independent TCL1 donor tumors (T1–T7; Supporting Information S1: Table 1), each transplanted into five or nine C57BL/6J recipient mice, we captured a broad range of disease kinetics while enabling the identification of conserved patterns of immune remodeling associated with leukemia progression. All seven cohorts showed progressive accumulation of circulating CD5^+^CD19^+^ CLL cells over time, confirming robust leukemia development across experiments. Disease progression rates varied substantially between cohorts, with mice reaching end‐stage disease between Days 22 and 60 after AT (Figure 1A; Supporting Information S1: Table 1; Supporting Information S3: Figure 1 for gating strategy). Despite these differences in progression rate, leukemic burden at end‐stage was remarkably similar across all cohorts, indicating convergence toward a comparable terminal disease state.

**Figure 2:**
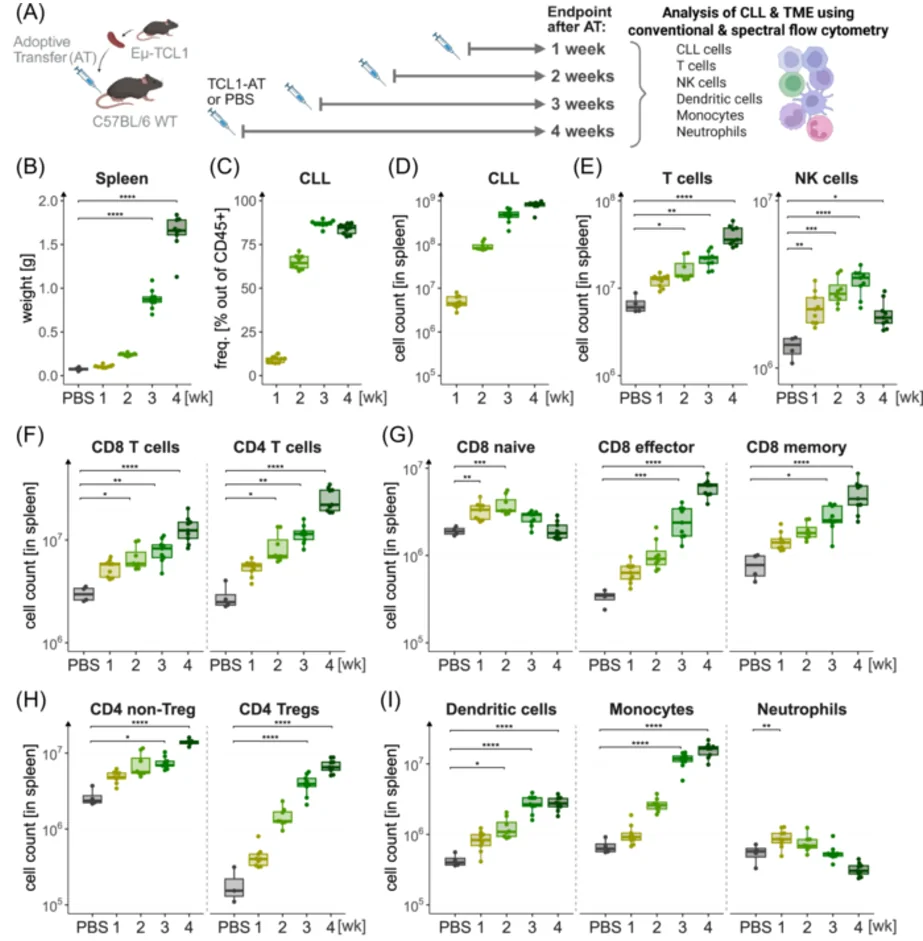
**Progressive chronic lymphocytic leukemia (CLL) burden and remodeling of tumor microenvironment (TME) in spleen after TCL1 adoptive transfer (TCL1‐AT). (A)** A schematic overview of the longitudinal in vivo study. Created with Biorender.com. For four consecutive weeks (wk), C57BL/6J recipient mice (*n* = 9 mice per time point, except Week 2 *n* = 8) received a single intravenous injection of 1 × 10^7^ Eμ‐TCL1 splenocyte–derived CLL cells. One control mouse per week received phosphate‐buffered saline (PBS). All mice were euthanized on the same day, with cohorts representing sequential stages of tumor development. Analysis of TCL1 CLL cells, T, natural killer (NK), and myeloid cells via conventional flow cytometry **(B–I)**. **(B)** Spleen weight, in grams. **(C, D)** TCL1 CLL cell frequency within CD45^+^ cells **(C)** and absolute TCL1 CLL cell count **(D)** in spleen. **(E–I)** Absolute cell count of T and NK cells **(E)**, CD8^+^ and CD4^+^ T cells **(F)**, naïve, effector and memory CD8^+^ T cells **(G)**, conventional CD4^+^ and CD4^+^ regulatory T cells (Tregs **H**), and dendritic cells, monocytes and neutrophils **(I)** in spleen. Box plots indicate medians with interquartile ranges (IQRs) (whiskers to 1.5 × IQR). One‐way analysis of variance (ANOVA) followed by Dunnett's multiple comparisons test (each group vs. PBS control). ****P < 0.0001, ***P ≤ 0.001, **P ≤ 0.01, and *P ≤ 0.05.

To identify immune populations consistently associated with leukemia progression, we monitored changes in monocyte and T‐cell subsets in the blood from pre‐transfer until end‐stage disease. The adaptive immune compartment underwent consistent remodeling across all TCL1‐AT experiments. Absolute numbers of CD8^+^ effector T cells increased substantially during leukemia progression and were markedly elevated at end stage compared with pre‐transfer samples and tumor‐naïve control mice (Figure 1B; Supporting Information S3: Figure 2A). The accumulation appeared stronger in mice reaching end‐stage disease later, indicating that a slower CLL disease course is associated with enhanced expansion of CD8^+^ effector T cells. Also, CD4^+^ effector T‐cell numbers increased over time, with a significantly higher increase compared to naïve mice an preferentially in mice that reached end‐stage disease later (Figure 1C; Supporting Information S3: Figure 2B). In line with observations in patients with CLL,^17^ this resulted in a progressive decline in the CD4/CD8 T‐cell ratio (Figure 1D; Supporting Information S3: Figure 2C). These findings indicate an early and reproducible activation of the cytotoxic T‐cell compartment in response to emerging leukemia.

Concomitant changes were observed within the myeloid compartment. Patrolling monocytes exhibited a striking and reproducible expansion in all TCL1‐AT experiments, with absolute numbers increasing steadily over time and reaching significantly higher levels at end‐stage than before AT or in tumor‐naïve controls (Figure 1E; Supporting Information S3: Figure 2D). In contrast, inflammatory monocytes showed only a modest increase in absolute cell numbers during disease progression (Supporting Information S3: Figure 2E,F). Analysis of monocyte subset composition further revealed a progressive and significant enrichment of patrolling monocytes within the total monocyte compartment, accompanied by a relative reduction of inflammatory monocytes (Supporting Information S3: Figure 2G,H). Despite variability in leukemia kinetics between individual experiments, these immune alterations were remarkably conserved. The reproducible expansion of patrolling monocytes and effector T cells suggests that TCL1‐driven leukemia induces coordinated remodeling of both innate and adaptive immune compartments early during disease development.

### Coordinated lymphoid and myeloid remodeling of the splenic compartment during CLL development

To characterize immune remodeling within the splenic TME during CLL progression, we established a longitudinal TCL1‐AT cohort representing defined stages of disease development (Figure 2A). For four consecutive weeks, C57BL/6J recipient mice (*n* = 9 each week) received a single i.v. injection of B‐cell–enriched splenocytes from one TCL1‐AT mouse model (T8; Supporting Information S1: Table 1). Mice injected with PBS were used as controls. All mice were euthanized on the same day, representing groups of 1, 2, 3, or 4 weeks post‐AT, allowing longitudinal assessment of defined stages of disease progression in the splenic compartment by flow cytometry.

**Figure 3:**
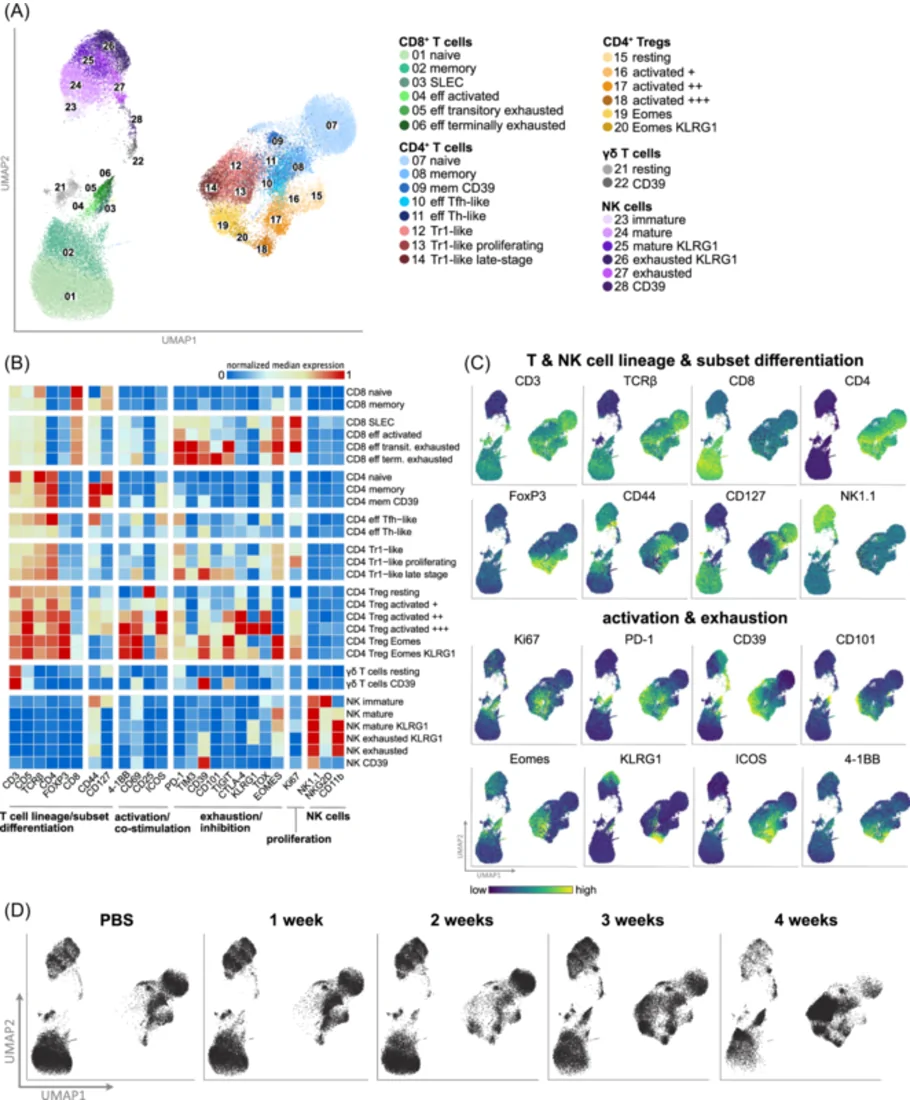
**Spectral flow cytometry identifies T‐cell heterogeneity in chronic lymphocytic leukemia (CLL) tumor microenvironment (TME).** Unsupervised analysis of the T and natural killer (NK) cell compartment in spleens of mice without (phosphate‐buffered saline [PBS]) and after 1–4 weeks of TCL1 adoptive transfer (TCL1‐AT) using a 33‐marker spectral flow cytometry antibody panel (*n* = 9 mice per time point, except for 2 weeks after AT, *n* = 8, PBS group *n* = 4). **(A)** Uniform Manifold Approximation and Projection (UMAP) projection of annotated T‐ and NK‐cell clusters (Phenograph) across all time points. **(B)** Heatmap displaying the min–max scaled (0–1) median marker intensities for each annotated cluster, with red indicating higher and blue indicating lower expression. **(C)** UMAP projection overlaid with T‐ and NK‐cell lineage and subset differentiation markers (above, CD3, TCRβ, CD8, CD4, FoxP3, CD44, CD127, and NK1.1) and activation and exhaustion markers (below, Ki67, PD‐1, CD39, CD101, Eomes, KLRG1, ICOS, and 4‐1BB). Marker intensities are represented by a color scale, with yellow indicating higher and blue indicating lower expression. **(D)** UMAPs stratified by time point, illustrating time‐specific cellular distributions.

As expected, leukemia progression was marked by a steady accumulation of CLL cells in the spleen, accompanied by progressive splenomegaly (Figure 2B). Both the frequency and absolute number of CLL cells in the spleen increased over time, indicating ongoing tumor expansion within this tissue (Figure 2C,D). In parallel, leukemic burden in the PB rose continuously, with increases in both absolute counts and CLL cell frequencies detected longitudinally (Supporting Information S3: Figure 3A,B). Consistent increases in CLL cell frequencies were also observed in LNs and BM, reflecting systemic dissemination of disease (Supporting Information S3: Figure 3B).

**Figure 4:**
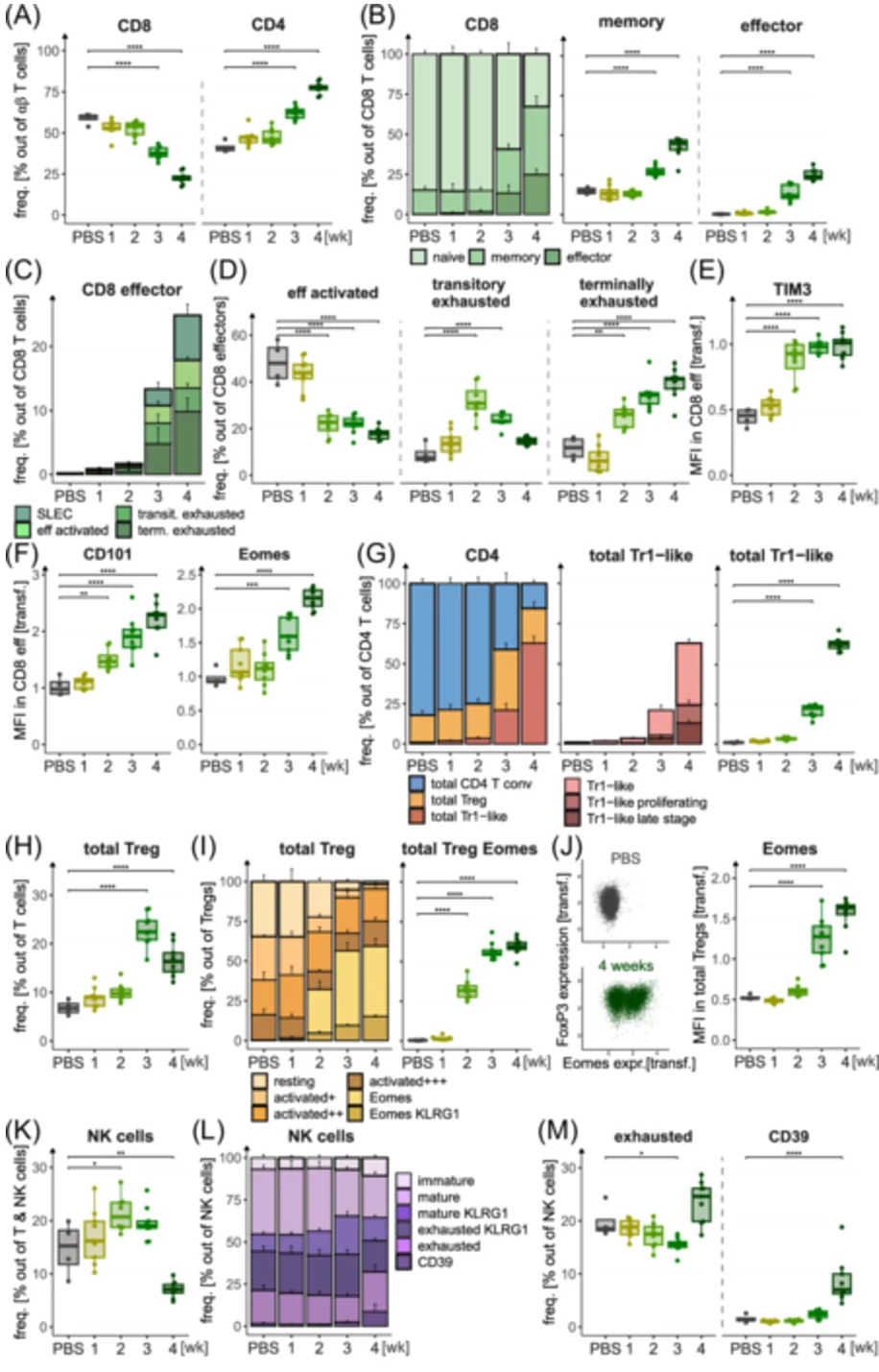
**Accumulation of exhausted CD8^+^ T cells, regulatory T cells (Tregs), and regulatory Type 1 (Tr1)‐like cells during chronic lymphocytic leukemia (CLL) progression.** Quantitative analysis of the T‐ and natural killer (NK)‐cell compartment in the spleen during CLL development using the spectral flow cytometry data shown in Figure 3. **(A)** Frequencies of CD8^+^ (left) and CD4^+^ T cells (right) over time (phosphate‐buffered saline [PBS] control, and 1–4 weeks [wk] post‐adoptive transfer [AT]). **(B)** Proportions of naïve, memory, and effector CD8^+^ T cells within CD8^+^ T cells, shown as stacked bar plot (left) and box plots (right). **(C, D)** Proportions of CD8^+^ effector subsets (short‐lived effector cells [SLECs], effector [eff] activated, transitory [transit.] exhausted, and terminally [term.] exhausted CD8^+^ T cells) within total CD8^+^ T cells (C, stacked bar plot) and CD8^+^ effector cells (D, box plots). **(E, F)** Quantification of marker expression (transformed [transf.] median fluorescent intensity [MFI]) of TIM3 (E), CD101 (F, left), and Eomes (F, right) in CD8^+^ effectors. **(G)** Proportions of total conventional (conv) CD4^+^ T cells, total Tregs, total Tr1‐like cells within CD4^+^ T cells, shown as stacked bar plot (left). Proportions of Tr1‐like subsets within CD4^+^ T cells, shown as stacked bar plot (middle). Frequency of total Tr1‐like cells within CD4^+^ T cells over time (right). **(H)** Percentage of total Tregs. **(I)** Proportions of Treg subsets within total Tregs, shown as stacked bar plot (left). Frequency of total Tregs Eomes within total Tregs (right). **(J)** Scatterplot of FoxP3 and Eomes expression (transf. MFI) in total Tregs from PBS control mice (above) and 4 weeks (below) after TCL1‐AT (left, all mice per group concatenated). Quantification of Eomes expression in total Tregs over time (right). **(K)** Frequencies of total NK cells within all groups. **(L)** Proportions of NK‐cell subsets within the NK‐cell compartment, shown as stacked bar plot. **(M)** Percentages of NK exhausted (left) and NK CD39 subset (right) within NK cells over time. **(A–M)** Stacked bars show subset proportions within indicated parent populations as mean with SD error bars; box plots indicate medians with interquartile ranges (IQRs) (whiskers to 1.5 × IQR). One‐way analysis of variance (ANOVA) followed by Dunnett's multiple comparisons test (each group vs. PBS control). ****P < 0.001, ***P ≤ 0.001, **P ≤ 0.01, and *P ≤ 0.05.

Concomitant with leukemic expansion, substantial remodeling of the T‐cell compartment was observed (Supporting Information S3: Figure 4 for gating strategy). Absolute numbers of total T cells and NK cells increased over the course of disease progression (Figure 2E) with a significant drop in NK‐cell counts at end‐stage disease (Supporting Information S3: Figure 3C). The increase in T cells was largely driven by a pronounced expansion of CD4^+^ T cells, which rose more markedly than CD8^+^ T cells and led to an increase in CD4/CD8 ratio, particularly at later stages of disease (Week 4) (Figure 2F; Supporting Information S3: Figure 3D). This is in contrast to the more pronounced rise of CD8^+^ T cells in the blood, which resulted in a drop of the CD4/CD8 ratio (Figure 1D).

Phenotypic analysis of CD8^+^ T cells revealed dynamic changes in differentiation states over time. An initial transient increase in naïve CD8^+^ T cells occurred during the early phase of disease (Weeks 1–2), followed by a pronounced decline at later stages (Figure 2G; Supporting Information S3: Figure 3E). In contrast, CD8^+^ effector and memory T‐cell subsets expanded steadily throughout leukemia progression, suggesting ongoing activation and differentiation of CD8^+^ T cells in response to the evolving TME (Figure 2G).

Within the CD4^+^ T‐cell compartment, conventional CD4^+^ T cells showed a massive expansion, most prominently at advanced disease stages (Week 4) (Figure 2H). In parallel, regulatory T cells (Tregs) displayed a modest but consistent increase over time, indicating a gradual enrichment of immunoregulatory mechanisms during CLL progression (Figure 2H). The concomitant expansion of effector/memory T cells and Tregs suggests a complex balance between immune activation and immune suppression in the CLL microenvironment.

**Figure 5:**
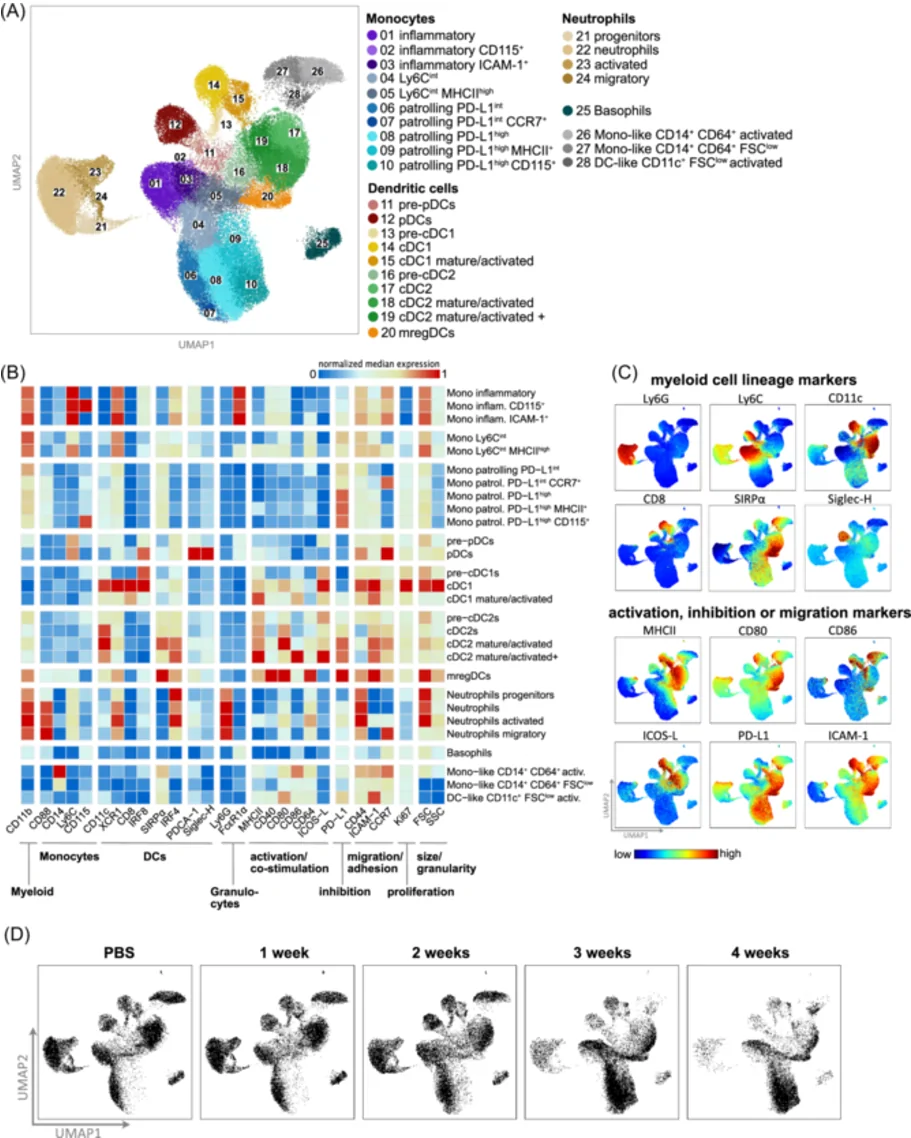
**Spectral flow cytometry reveals myeloid cell heterogeneity in the chronic lymphocytic leukemia (CLL) tumor microenvironment (TME).** Unsupervised analysis of the myeloid cell compartment in mouse spleens before (phosphate‐buffered saline [PBS]) and at 1–4 weeks after TCL1 adoptive transfer (TCL1‐AT) using a 34‐marker spectral flow cytometry antibody panel (*n* = 9 mice per time point, except for 2 weeks after adoptive transfer (AT), *n* = 8, PBS group *n* = 4). **(A)** Uniform Manifold Approximation and Projection (UMAP) projection of annotated monocyte, dendritic cell (DC), and neutrophil clusters (Phenograph) across all time points. **(B)** Heatmap displaying the min–max scaled (0–1) median marker intensities for each annotated cluster, with red indicating higher and blue indicating lower expression. **(C)** UMAP projection overlaid with myeloid cell lineage and subset differentiation markers (Ly6G, Ly6C, CD11c, CD8, SIRPα, and Siglec‐H) and activation, inhibition and migration markers (MHC II, CD80, CD86, ICOS‐L, PD‐L1, and ICAM‐1). Marker intensities are represented by a color scale, with red indicating higher and blue indicating lower expression. **(D)** UMAPs stratified by time point, illustrating time‐specific cellular distributions.

Beyond lymphoid populations, myeloid cell compartments were also dynamically altered (Supporting Information S3: Figure 5 for gating strategy). Absolute numbers of dendritic cells (DCs) and monocytes increased steadily over disease progression (Figure 2I), consistent with enhanced myeloid involvement in the leukemic microenvironment. Neutrophils, in contrast, exhibited a transient rise at the earliest time point (Week 1), followed by a continuous moderate decline in absolute counts at later time points (Figure 2I; Supporting Information S3: Figure 3F), suggesting differential regulation of myeloid subsets during leukemia development.

To determine whether the immune alterations observed in the synchronized longitudinal cohort were reproducible across independent TCL1 tumors, we analyzed the splenic immune composition at end‐stage disease in the seven independent TCL1‐AT experiments described in Figure 1. Despite substantial differences in disease kinetics, all cohorts developed marked splenomegaly and accumulated comparable absolute numbers of leukemic CD5^+^CD19^+^ cells within the spleen (Supporting Information S3: Figure 3G–K). Interestingly, endpoint spleens exhibited a pronounced expansion of CD4^+^ T cells, whereas the CD8^+^ T cells expanded more prominently in the blood, resulting in higher CD4/CD8 ratios in the spleen than in blood in five of the seven analyzed TCL1‐AT cohorts (Supporting Information S3: Figure 3L). Moreover, we observed a consistent increase in absolute monocyte counts and a moderate increase in total DC numbers at end‐stage disease (Supporting Information S3: Figure 3M,N).

Together, these data demonstrate that the major lymphoid and myeloid alterations identified during longitudinal disease progression are highly reproducible across independent TCL1 tumors and converge toward a common immune landscape at advanced stages of CLL development. In particular, the progressive shift from naïve to effector and memory T‐cell phenotypes, alongside increasing Treg frequencies and expanding myeloid populations, underscores the dynamic immune remodeling that occurs during leukemia evolution in the TCL1‐AT model. These observations establish a robust framework for the subsequent high‐dimensional characterization of immune remodeling during CLL progression.

### High‐dimensional profiling uncovers evolving T‐cell states in CLL

To enable in‐depth characterization of immune heterogeneity during CLL progression in the TCL1‐AT mouse model, we established and validated high‐dimensional spectral FC panels optimized to capture phenotypic and functional diversity within T and myeloid cell compartments. Two antibody panels comprising either 34 or 33 antibodies were designed to simultaneously resolve lineage identity, differentiation state, activation, and exhaustion features of T and NK cells (Panel 1) and myeloid cells (Panel 2).

**Figure 6:**
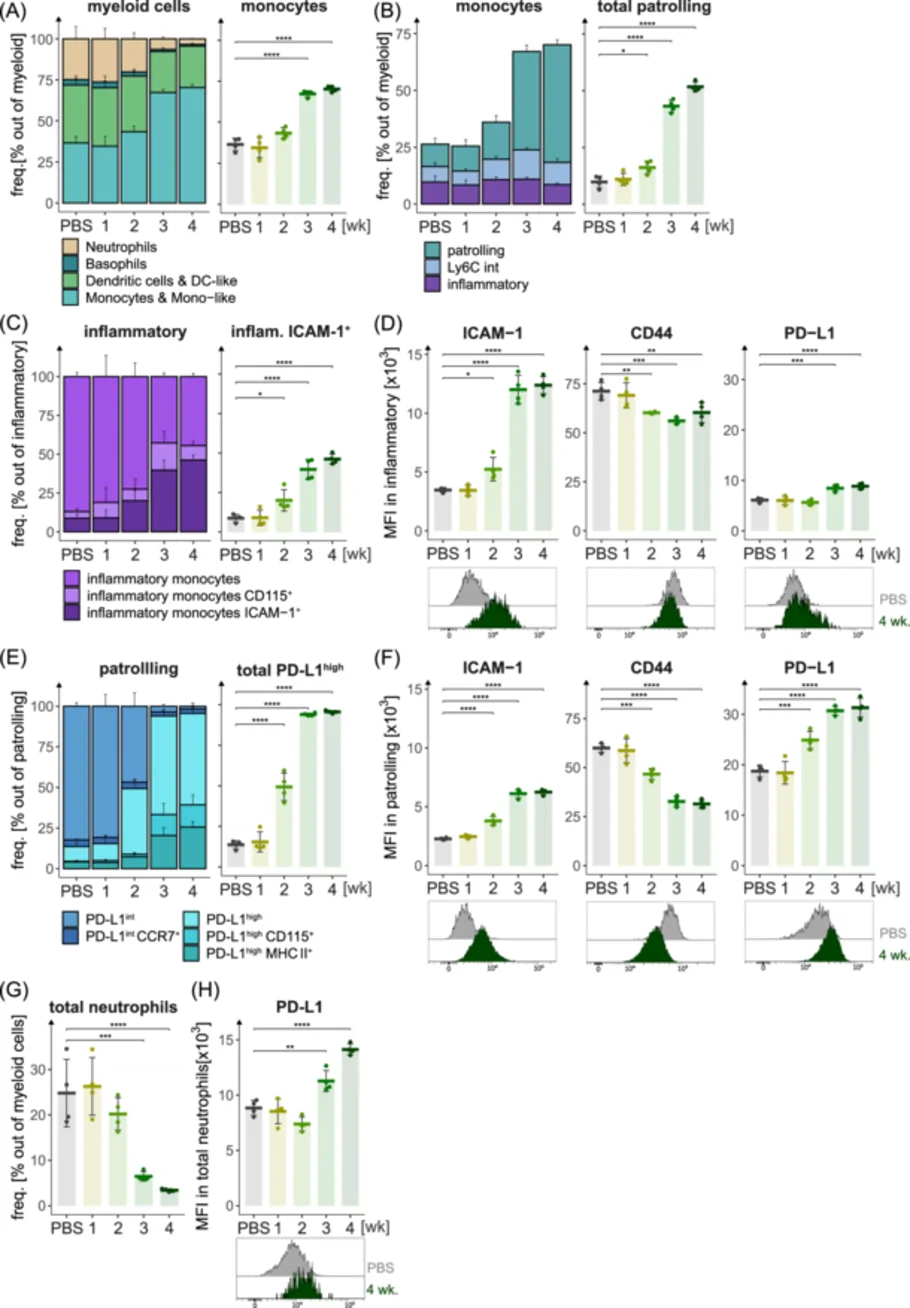
**Myeloid cell rewiring during chronic lymphocytic leukemia (CLL) development favors patrolling monocytes.** Quantitative analysis of monocytes and neutrophils in the spleen during CLL progression using the spectral flow cytometry data shown in Figure 5. **(A)** Proportions of neutrophils, basophils, dendritic cells (DCs) and DC‐like cells as well as monocytes and Mono‐like cells within myeloid cells (left). Frequency of monocytes within myeloid cells over time (right, phosphate‐buffered saline [PBS] control, and 1–4 weeks (wk) post‐adoptive transfer [AT]). **(B)** Proportions of patrolling, Ly6C^int^, and inflammatory monocytes within myeloid cells (left). Percentage of total patrolling monocytes within myeloid cells (right). **(C)** Proportions of inflammatory monocyte subsets within total inflammatory monocytes (left). Frequency of ICAM‐1^+^ inflammatory monocytes within total inflammatory monocytes (right). **(D)** Quantifications of ICAM‐1, CD44, and PD‐L1 expression (median fluorescent intensity [MFI]) in total inflammatory monocytes over time (above). Respective histograms in total inflammatory monocytes from PBS control mice (gray) and 4 weeks (dark green) after AT (below, all mice per group concatenated). **(E)** Proportions of patrolling monocyte subsets within patrolling monocytes (left). Frequency of total PD‐L1^high^ patrolling monocytes within total patrolling monocytes (right). **(F)** Quantifications of ICAM‐1, CD44, and PD‐L1 expression (MFI) in total patrolling monocytes over time (above). Respective histograms in total patrolling monocytes from PBS control mice (gray) and 4 weeks (dark green) after AT (below, all mice per group concatenated). **(G)** Frequency of total neutrophils within myeloid cells. **(H)** Quantifications of PD‐L1 expression (MFI) in total neutrophils over time. Histograms show PD‐L1 expression in total neutrophils from PBS control mice (gray) and 4 weeks (dark green) after AT (below, all mice per group concatenated). **(A–H)** Stacked bars show subset proportions within indicated parent populations as mean with SD error bars; bar plots indicate mean with SD. One‐way analysis of variance (ANOVA) followed by Dunnett's multiple comparisons test (each group vs. PBS control). ****P < 0.0001, ***P ≤ 0.001, **P ≤ 0.01, and *P ≤ 0.05.

**Figure 7:**
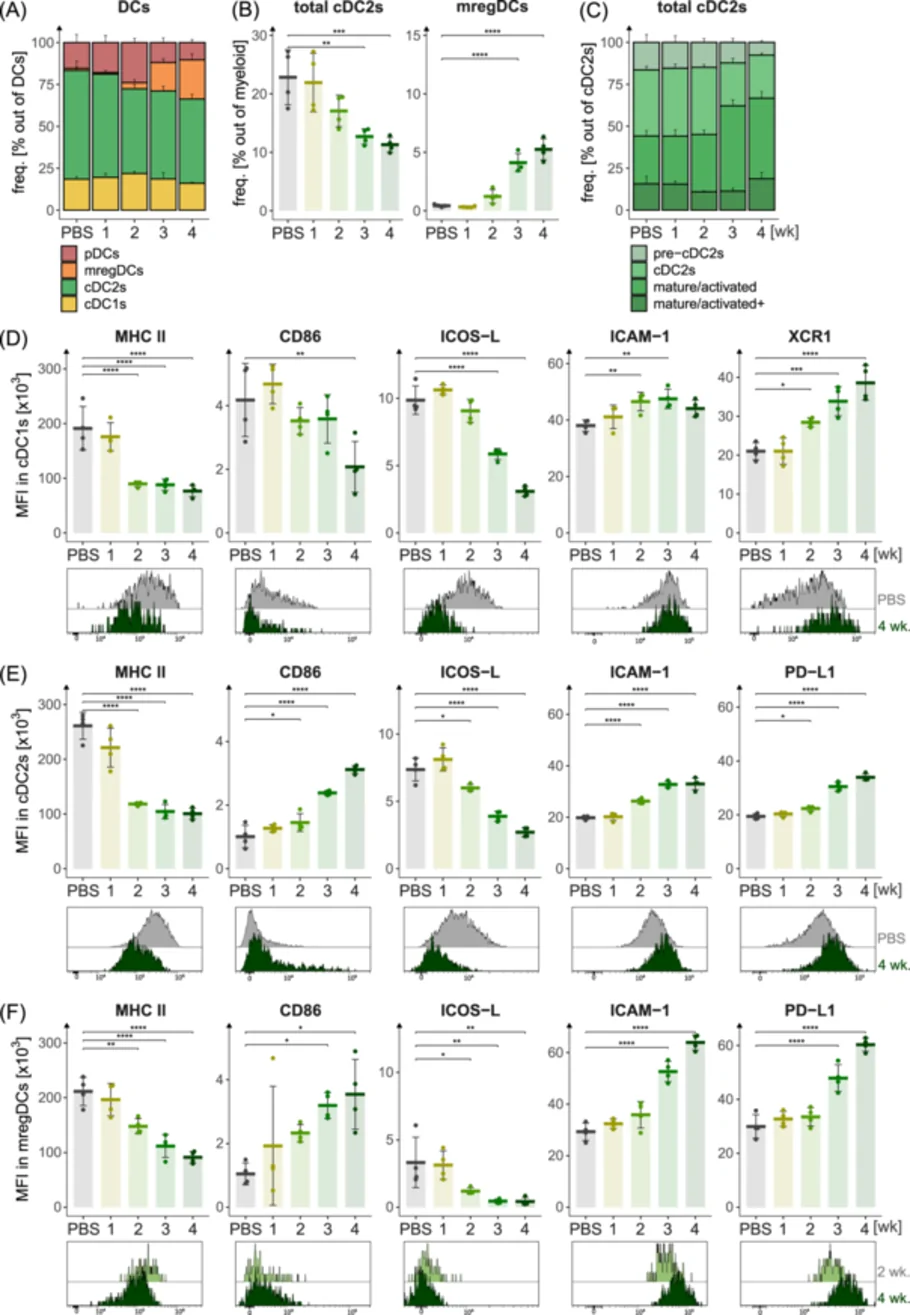
**Myeloid cell phenotypic changes during chronic lymphocytic leukemia (CLL) progression promote the expansion of mature immune regulatory dendritic cells (mregDCs) while reducing the antigen presentation ability in conventional dendritic cell (cDC) subsets.** Quantitative analysis of dendritic cells (DCs) in the spleen during CLL progression using the spectral flow cytometry data shown in Figure 5. **(A)** Proportions of conventional Type 1 (cDC1s), Type 2 (cDC2s), plasmacytoid (pDCs), and mregDCs within DCs, shown as a stacked bar plot. **(B)** Frequency of total cDC2s (left) and mregDCs (right) within myeloid cells. **(C)** Proportions of cDC2 subset in total cDC2s, shown as a stacked bar plot. **(D–F)** Quantification of MHC II, CD86, ICOS‐L, ICAM‐1, and PD‐L1 or XCR1 expression (median fluorescent intensity [MFI]) in total cDC1s (D), cDC2s (E), and mregDCs (F) (above). Histograms of marker expression in respective DC subsets from PBS control mice (D, E, gray), 2 weeks (F, light green), or 4 weeks (D–F, dark green) after AT (below, all mice per group concatenated). **(A–F)** Stacked bars show subset proportions within indicated parent populations as mean with SD error bars; bar plots indicate mean with SD. One‐way analysis of variance (ANOVA) followed by Dunnett's multiple comparisons test (each group vs. PBS control). ****P < 0.0001,***P ≤ 0.001, **P ≤ 0.01, and *P ≤ 0.05.

**Figure 8:**
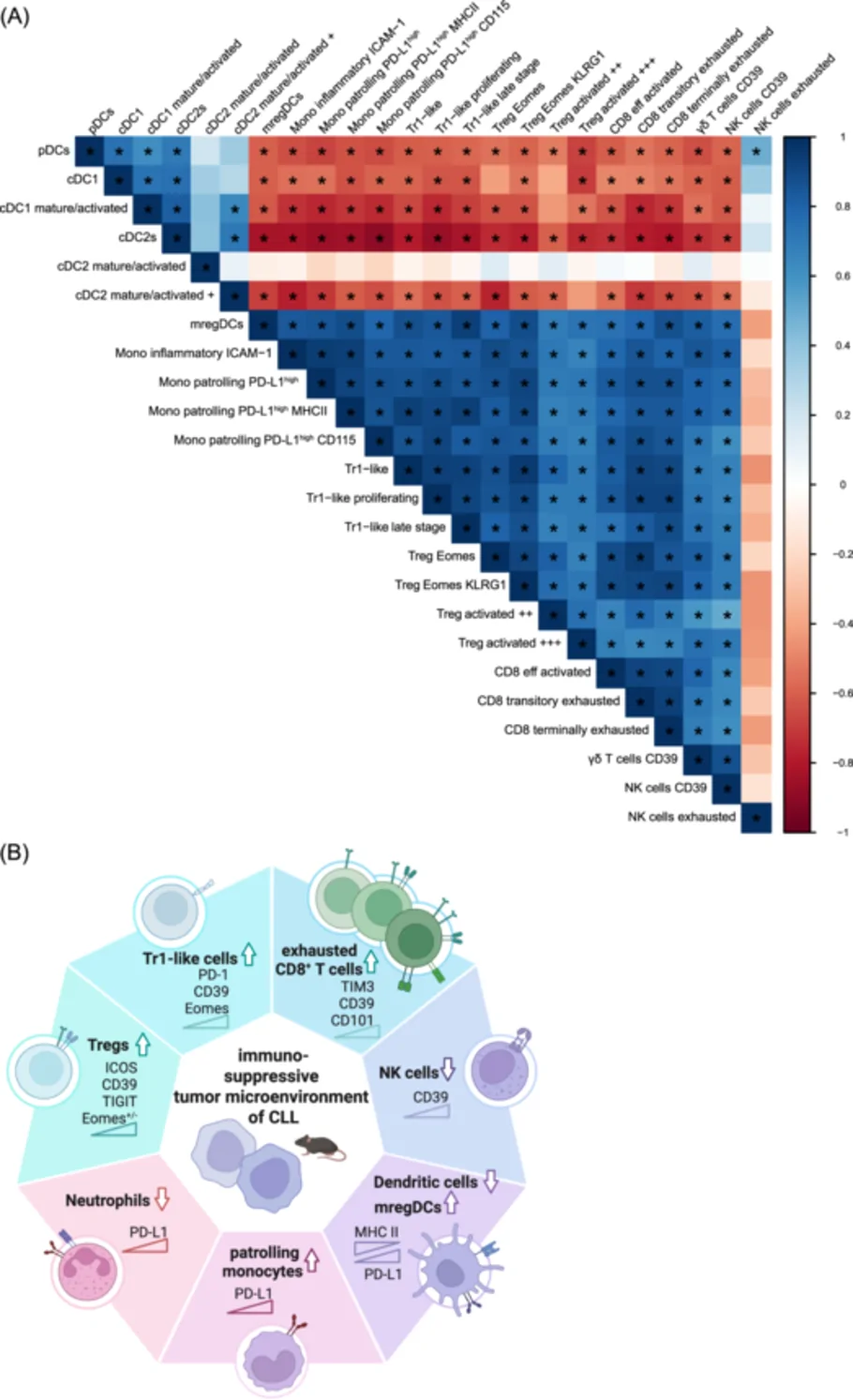
**Coordinated remodeling of T‐cell and myeloid compartments during chronic lymphocytic leukemia (CLL) progression. (A)** Heatmap visualizing Spearman rank correlations across all time points combined for cell population frequencies of T/natural killer (NK) and myeloid cells in spleens of mice (*n* = 20), with blue indicating positive and red indicating negative correlations. Significant correlations (adjusted for multiple comparisons using the Benjamini–Hochberg method, P < 0.05) marked with asterisks. **(B)** Schematic illustration summarizing the T, NK, and myeloid cell compartment contributing to the immunosuppressive tumor microenvironment in CLL in the Eµ‐TCL1 mouse model. Created with Biorender.com.

A standardized spectral FC workflow was performed, incorporating optimized staining, rigorous pre‐gating, and a hierarchical population tree to define major immune lineages before unsupervised clustering (Supporting Information S3: Figure 6, Panel 1; Supporting Information S1: Table 2). Cluster identities were validated by parallel manual gating, demonstrating strong concordance between computational and conventional approaches (Supporting Information S3: Figure 7, Panel 1). Data quality and reproducibility were further confirmed by consistent signal distributions across groups and individual samples (Supporting Information S3: Figure 8, Panel 1), establishing spectral FC as a reliable platform for longitudinal immune profiling in the TCL1‐AT model.

Using the established antibody panel, we performed deep phenotypic profiling of T cells to resolve dynamic changes in activation and exhaustion states during CLL progression. Unsupervised clustering visualized by Uniform Manifold Approximation and Projection (UMAP) revealed a diverse landscape of phenotypically distinct T‐cell subsets (Figure 3A). These clusters were defined by differential expression of the full 33‐marker panel and summarized in a heatmap highlighting heterogeneity across lineage, differentiation, activation, and exhaustion markers (Figure 3B). To enable biological interpretation, clusters were subsequently annotated by projecting selected canonical markers onto the UMAP (Figure 3C; Supporting Information S3: Figure 9), including lineage and subset markers (CD3, TCRβ, CD8, CD4, FoxP3, CD44, CD127, and NK1.1) that delineated conventional T cells, Tregs, and NK cells, as well as activation and exhaustion markers (Ki67, PD‐1, CD39, CD101, Eomes, KLRG1, ICOS, and 4‐1BB).

Beyond naïve and memory CD8^+^ T cells that were defined by the expression levels of CD44 and CD127 as described before,^18^ multiple CD8^+^ effector subsets were identified, including short‐lived effector cells (SLECs) expressing KLRG1, activated, transitory, and terminally exhausted effector populations based on PD‐1, TIM3, CD39, and CD101 expression (Supporting Information S3: Figures 6C and 7; Supporting Information S1: Table 2). Within the CD4^+^ compartment, one naïve, two memory, one effector T follicular helper (Tfh)‐like, and one T helper (Th)‐like subset were resolved (Supporting Information S3: Figures 6C and 7; Supporting Information S1: Table 2). Notably, we identified distinct clusters of FoxP3‐negative regulatory Type 1 (Tr1)‐like cells expressing Eomesodermin (Eomes) and either the proliferation marker Ki67 or several exhaustion markers. Tregs, defined by the expression of FoxP3^+^, comprised one resting and several activated clusters with increasing expression of inhibitory and co‐stimulatory markers. To our surprise, we identified non‐canonical Eomes‐expressing Tregs, which we separated into two clusters based on KLRG1 expression. In addition to αβ T cells, γδ T and NK cells were resolved into phenotypically distinct subsets, including immature and mature NK‐cell populations with progressive CD39 expression.

Longitudinal analysis revealed pronounced temporal shifts in T‐cell composition (Figure 3D). While control and early post‐transfer samples were dominated by naïve T‐cell clusters, advanced disease stages showed accumulation of CD8^+^ effector populations and Tr1‐like cells, indicating progressive remodeling of the T‐cell compartment.

Together, these data demonstrate that high‐dimensional spectral FC enables precise resolution of longitudinal and clonal dynamics within the T‐ and NK‐cell compartments during CLL progression in mice. This approach enables tracking of population shifts over time and identifies phenotypically distinct, non‐canonical immune subsets, including Eomes‐expressing Tregs, underscoring its utility for refining immune classification in complex tumor‐associated immune landscapes.

### Emergence of exhausted and regulatory T cells defines a suppressive microenvironment in CLL

We next used the spectral FC data to determine how longitudinal immune remodeling translates into functional immune suppression during CLL development by quantifying temporal changes in effector and regulatory lymphocyte populations. The proportions of T‐ and NK‐cell subsets within individual mice demonstrated low within‐group variability, supporting the robustness of the groups with different disease stages (Supporting Information S3: Figure 10). As the disease progressed, the T‐cell compartment in the spleen underwent a marked compositional shift with a relative loss of CD8^+^ T cells and a concomitant expansion of CD4^+^ T cells (Figure 4A) confirming the conventional flow cytometry data shown above (Figure 2F). This imbalance suggests a gradual erosion of cytotoxic CD8^+^ T‐cell potential within the TME, while pointing to a potentially important role of CD4^+^ T‐cell subsets. Therefore, we sought to characterize both populations in greater depth.

Within the CD8^+^ T‐cell compartment, CLL progression was associated with a pronounced redistribution of differentiation states. While memory and effector CD8^+^ T cells expanded over time, naïve CD8^+^ T cells declined steadily (Figure 4B; Supporting Information S3: Figure 11A), consistent with sustained antigen exposure and previously reported findings in CLL.^19^, ^20^ The proportions of all CD8^+^ effector subsets out of all CD8^+^ T cells increased over time (Figure 4C); however, a dynamic shift from functional to dysfunctional states was observed (Figure 4D). SLECs showed a modest relative decrease compared to the other CD8^+^ effector subsets, whereas activated CD8^+^ effectors were progressively lost (Figure 4C,D; Supporting Information S3: Figure 11B). In contrast, transitory exhausted CD8^+^ T cells relatively increased early during disease progression (Week 2), followed by a dominant relative accumulation of terminally exhausted CD8^+^ effector cells at later stages (Figure 4C,D; Supporting Information S3: Figure 11C). This was further supported by an increase in expression of well‐established markers of exhaustion in CD8^+^ effector T cells, including PD‐1, CD39, TIM3, CD101, and Eomes (Figure 4E,F; Supporting Information S3: Figure 11D,E), indicating a stepwise transition from activation to exhaustion over time.

To validate these findings across independent TCL1 tumors, we analyzed CD8^+^ T‐cell phenotypes at end‐stage disease in the seven TCL1‐AT cohorts described in Figure 1. Consistent with the longitudinal spectral FC data, all cohorts exhibited a marked enrichment of exhausted CD8^+^ T‐cell populations at end‐stage disease, confirming that progressive CD8^+^ T‐cell dysfunction is a robust and reproducible feature of TCL1‐driven CLL irrespective of disease kinetics (Supporting Information S3: Figure 11F; Supporting Information S3: Figure 12 for gating strategy).

We further analyzed the CD4^+^ T‐cell compartment, which showed parallel but distinct changes. Conventional CD4^+^ T cells, particularly naïve subsets, declined markedly during disease progression (Figure 4G; Supporting Information S3: Figure 13A). In parallel, Tr1‐like CD4^+^ T cells expanded substantially (Figure 4G; Supporting Information S3: Figure 13B).

Tregs further contributed to the evolving suppressive landscape. The frequency of total Tregs increased during leukemia progression (Figure 4H), although a relative reduction at Week 4 compared to Week 3 reflected a more pronounced expansion of other CD4^+^ subsets. Phenotypic analysis revealed a loss of resting Tregs accompanied by a marked gain of activated Treg populations with increasing expression of markers linked to their suppressive function, including CTLA4, ICOS, PD‐1, TIGIT, CD39, and 4‐1BB (Figure 4I; Supporting Information S3: Figure 13C, D, and F). Notably, an unexpected population of Tregs expressing both FoxP3 and the transcription factor Eomes accumulated over time (Figure 4I). The co‐expression of FoxP3 and Eomes was confirmed at the single‐cell level by flow cytometric dot plot analysis (Figure 4J), supporting the presence of a distinct Eomes^+^ Treg subset emerging during disease progression. These cells showed a transient increase in PD‐1, TIGIT, and CD39 expression up to Week 2, and a loss of CTLA4 and ICOS expression over time (Supporting Information S3: Figure 13E,G), suggesting distinct regulation and function of this regulatory T‐cell subset.

Importantly, increased frequencies of the Eomes^+^ Treg subset as well as of Tr1‐like cells were observed in all independent TCL1‐AT cohorts at end‐stage disease (Supporting Information S3: Figure 14A,B), demonstrating that expansion of these regulatory populations is a highly conserved feature of TCL1‐driven CLL. Together with the accumulation of exhausted CD8^+^ T cells, these data support the establishment of a reproducible immunosuppressive T‐cell landscape during leukemia progression.

Monitoring the dynamic changes in γδ T cells, we observed transient increases in frequency during intermediate stages of disease progression, followed by a drop in percentage (Supporting Information S3: Figure 15A,B). Phenotypic analysis revealed a qualitative shift characterized by a gain in CD39‐expressing subsets (Supporting Information S3: Figure 15C,D), suggesting a transition toward an exhausted or suppressive phenotype, mirroring exhaustion trajectories observed in conventional T‐cell populations.

NK cells, which represent a minor population of the cytotoxic effector cell compartment (Supporting Information S3: Figure 15A), increased transiently during early disease stages, peaking around Week 2, followed by a decline at later time points (Figure 4K; Supporting Information S3: Figure 15A). This reduction was accompanied by a shift toward more terminally differentiated KLRG1‐expressing NK cells and an increasing acquisition of an exhausted phenotype, with a pronounced shift toward CD39‐expressing NK‐cell states observed at advanced disease stages (Figure 4L,M; Supporting Information S3: Figure 15E).

Collectively, these data demonstrate that CLL progression is associated with a gradual loss of functional effector lymphocytes and a concomitant accumulation of exhausted effector cells and regulatory T‐cell populations. The emergence of terminally exhausted CD8^+^ T cells, Tr1‐like CD4^+^ T cells, and Eomes‐expressing Tregs defines a progressively suppressive TME that likely limits effective adaptive immune control of CLL.

### High‐dimensional profiling unveils complex myeloid diversity in the CLL microenvironment

To extend our detailed examination of lymphoid compartments, we employed a new high‐dimensional spectral FC panel to analyze myeloid cell diversity and behavior throughout CLL progression. This panel was tailored to distinguish lineage identity, activation state, migration ability, and inhibitory traits of neutrophil, monocyte, and DC subsets within the CLL microenvironment, offering a comprehensive overview of myeloid changes during disease development (Supporting Information S3: Figures 16–19).

Unsupervised clustering with Phenograph^21^ and UMAP^22^ visualization uncovered a complex, diverse landscape of multiple monocyte, neutrophil, and DC subsets (Figure 5A). These clusters were annotated based on differential expression of numerous lineage, activation, migration, and inhibitory markers (Supporting Information S1: Table 3), aided by a detailed heatmap (Figure 5B), and UMAPs colored by marker expression (Figure 5C; Supporting Information S3: Figure 19). The monocyte compartment consisted of three inflammatory clusters, five patrolling clusters, and three Ly6C^int^ monocyte populations. We also identified two monocyte‐like clusters with high CD14 expression and one DC‐like cluster with low forward scatter and an activated profile. In the DC compartment, three conventional DC1 (cDC1) clusters, four cDC2 clusters, two plasmacytoic DC (pDC) clusters, and a group of mature DCs enriched in immunoregulatory molecules (mregDCs) were observed. mregDCs are a conserved population of CCR7^+^, LAMP3^+^ DCs characterized by enhanced antigen presentation, co‐stimulatory molecules, immunomodulatory markers such as TIM3 and PD‐L1/2, and cytokine production.^23^ These cells, variably called migratory DCs, regulatory DCs, DC3s, or LAMP3^+^ DCs across studies, reflect ongoing debates over nomenclature despite similar transcriptional and functional properties.^24^, ^25^, ^26^ Additionally, four distinct neutrophil clusters and one basophil cluster were identified.

Mapping key subset markers onto the UMAP highlighted the functional diversity, particularly among activation and migration markers in myeloid populations (Figure 5B,C). Notably, DC subsets such as mregDCs and cDC2s showed differences indicative of functional specialization in antigen presentation and tissue migration. Monocyte subsets displayed distinct inflammatory and patrolling signatures, with patrolling cells expressing higher PD‐L1. Neutrophil clusters showed variation in activation and inhibitory marker expression, revealing functional heterogeneity within this group (Figure 5C).

Longitudinal UMAP plots showed ongoing remodeling of myeloid subsets as CLL progressed (Figure 5D), with shifts in inflammatory and patrolling monocytes, mregDCs, cDC2s, and neutrophils, reflecting evolving innate immune responses as the disease progressed.

In summary, these findings indicate that CLL progression is associated with extensive and coordinated changes in the myeloid compartment. High‐dimensional spectral FC enables detailed resolution of myeloid subpopulations and their dynamic changes, providing a valuable framework for understanding how myeloid heterogeneity shapes the immunosuppressive TME during CLL development.

### Expansion of immunosuppressive patrolling monocytes during leukemia progression

We next examined how monocyte and neutrophil populations evolve during CLL progression and contribute to an immunosuppressive TME. Similar to the proportions of T‐ and NK‐cell subsets, the myeloid cell compartment within individual mice showed low within‐group variability, further supporting the robustness of the data (Supporting Information S3: Figure 20A). The frequency of total monocytes increased over time, driven primarily by expansion of patrolling monocytes, whereas inflammatory monocyte frequencies remained stable within the myeloid compartment (Figure 6A,B; Supporting Information S3: Figure 20B). Among inflammatory monocytes, the ICAM‐1^+^ subset expanded, indicating enhanced activation and adhesion potential (Figure 6C,D; Supporting Information S3: Figure 20C).

In addition, patrolling monocytes shifted toward immunosuppressive states: subsets with intermediate PD‐L1 expression decreased, whereas PD‐L1^high^ cells expanded (Figure 6E,F; Supporting Information S3: Figure 20D,E). Among the latter, we identified a cell cluster with higher MHC II expression and a cluster with pronounced CD115 expression, a molecule recently linked to failure of CAR T‐cell therapy.^27^ PD‐L1 levels were consistently higher in patrolling than in inflammatory monocytes (Figure 6D,F), confirming previous observations and highlighting the dominant immunoregulatory role of this subset.^28^ In parallel, CD44 expression declined in inflammatory monocytes (Figure 6D), and even more so in patrolling monocytes (Figure 6F), suggesting a shift in monocyte adhesion and migratory behavior during CLL development. Importantly, increased frequencies of the patrolling monocyte subset were confirmed in spleen samples of the seven TCL1‐AT cohorts presented in Figure 1 at end‐stage disease (Supporting Information S3: Figure 20F), showing that expansion of these immunosuppressive monocytes is a highly conserved feature of TCL1‐driven CLL.

Neutrophil frequency decreased at Weeks 3 and 4, with no major change in subset composition (Figure 6G; Supporting Information S3: Figure 21A,B). PD‐L1 expression on neutrophils increased (Figure 6H), indicating immunosuppressive features in advanced disease. The relative decrease of neutrophils within the myeloid cell compartment was also observed in the seven TCL1‐AT cohorts at end‐stage disease (Supporting Information S3: Figure 21C).

Together, these results demonstrate that CLL progression drives expansion and functional rewiring of patrolling monocytes, a loss of neutrophils, and acquisition of a suppressive phenotype at late stages. These changes likely contribute to an immunosuppressive TME that limits effective anti‐leukemic responses.

### Reduction of conventional Type 2 DCs and increase of regulatory DCs in CLL

We further examined how DC subsets change over time. The relative frequency of cDC2s within the myeloid cell group gradually decreased as the disease progressed, whereas cDC1s remained unchanged over time (Figure 7A,B; Supporting Information S3: Figure 22A–C). In the cDC2 group, there was an increase in mature or activated cells, suggesting that residual cDC2s become activated during leukemia progression (Figure 7C; Supporting Information S3: Figure 22D,E).

Alongside the decline of cDC2s, mregDCs appeared early in disease progression, first detectable by Week 2 and steadily increasing through Week 4 (Figure 7B). Their accumulation indicates a significant shift toward immunoregulatory DC states, consistent with their known roles in suppressing T‐cell activation and fostering immune tolerance.

When analyzing the phenotype of DC subsets over time during CLL development, we found that XCR1 was upregulated on cDC1s, consistent with a more mature state, as indicated by upregulation of the co‐stimulatory molecule CD40 and a transient upregulation of the adhesion molecule ICAM‐1 (Figure 7D; Supporting Information S3: Figure 22F). However, this contrasts with reduced expression of MHC II and the co‐stimulatory molecules CD86, ICOS‐L, and CD44, a migration marker (Figure 7E; Supporting Information S3: Figure 22F). PD‐L1 was slightly upregulated over time on cDC1s (Supporting Information S3: Figure 22F). This phenotype indicates a tolerogenic state that supports expansion of exhausted cytotoxic T cells and Tregs.

cDC2s and mregDCs displayed similar changes in the marker expression to that of cDC1s, but with subtle differences. They were characterized by MHC II downregulation and upregulation of CD80, CD86, CD40, ICAM‐1, and PD‐L1, downregulation of ICOS‐L, and minor changes in CD44 expression (Figure 7E,F; Supporting Information S3: Figure 22E, G, and H). Notably, even activated cDC2s showed lower MHC II expression (Supporting Information S3: Figure 22E), indicating that their antigen‐presenting ability remains compromised despite activation.

To summarize, downregulation of distinct markers suggests restricted antigen presentation, reduced migration capacity, and reduced Th1/Tfh support, whereas upregulation of some markers may drive T‐cell exhaustion and Treg expansion.

### Coordinated remodeling of T‐cell and myeloid compartments during CLL progression

To assess whether adaptive and innate immune alterations evolve in a coordinated manner during CLL development, we performed correlation analysis across all immune subsets throughout disease progression. This integrative analysis revealed that immunoregulatory myeloid populations including mregDCs and PD‐L1^high^ patrolling monocytes showed strong positive correlations with Tregs, Tr1‐like CD4^+^ T cells, and terminally exhausted CD8^+^ T cells (Figure 8A; Supporting Information S1: Tables 4 and 5). Conversely, mature and immunostimulatory DC subsets were consistently anti‐correlated with these regulatory and exhausted T‐cell populations, indicating that loss of antigen‐presenting and co‐stimulatory capacity accompanies adaptive immune suppression.

Together, these opposing correlation patterns define reciprocally evolving immune modules: an early stimulatory axis composed of neutrophils, mature DCs and functional effector lymphocytes, and a later suppressive axis comprising regulatory myeloid cells and exhausted cytotoxic T cells and Tregs. This coordinated shift supports a model in which after an initial antitumor activity at early disease stages, progressive myeloid polarization reinforces T‐cell exhaustion and regulatory cell differentiation, ultimately leading to loss of immune control in later stages of CLL.

In summary (Figure 8B), our novel findings and insights provide a conceptual framework for immune escape in CLL and highlight coordinated myeloid–T‐cell reprogramming as a key target to enhance immunotherapeutic efficacy.

## DISCUSSION

In this study, we provide a comprehensive temporal dissection of immune dynamics during CLL progression in the TCL1‐AT mouse model, revealing coordinated remodeling of adaptive and innate compartments that culminates in an immunosuppressive TME. Our longitudinal profiling shows that early expansion of adaptive cytotoxic responses, including CD8^+^ effector T and NK cells, is gradually supplanted by dysfunctional and exhausted states, consistent with evidence from CLL patients showing broad T‐cell exhaustion phenotypes in blood and lymphoid tissue, including precursor and terminally exhausted CD8^+^ T cells.^8^

In addition to qualitative changes in T‐cell states, we observed marked alterations in T‐cell composition during CLL progression. In the PB of TCL1‐AT mice, the CD4/CD8 ratio progressively declined due to expansion of CD8^+^ effector cells, a phenomenon that mirrors observations in patients with CLL, where reduced CD4/CD8 ratios have been associated with disease progression and chronic antigenic stimulation.^17^ Interestingly, this pattern contrasted with the splenic compartment, where CD4^+^ T cells accumulated more prominently than CD8^+^ T cells during advanced disease stages. This compartment‐specific divergence highlights the importance of the local microenvironment in shaping immune responses. Notably, we previously observed a similar enrichment of CD4^+^ over CD8^+^ T cells in malignant LNs from patients with CLL using integrated single‐cell, mass cytometry, and spatial profiling approaches,^8^ suggesting that lymphoid tumor niches may preferentially support the expansion and retention of CD4^+^ T‐cell populations. Together, these findings raise the possibility that the CD4‐dominated immune landscape observed in the TCL1 spleen reflects features of human lymphoid disease sites and warrants further investigation in paired blood and LN samples from patients with CLL. The emergence of multiple regulatory CD4^+^ T‐cell subsets, including Tr1‐like cells and a population of Eomes‐expressing FoxP3^+^ Tregs, underscores the complexity of adaptive immune suppression in CLL. While conventional FoxP3^+^ Tregs have long been recognized as barriers to effective antitumor immunity, it has become increasingly clear that additional CD4^+^ subsets actively contribute to immune escape. Among these, Tr1 cells represent a critical but incompletely understood population.^29^ Tr1 cells are FoxP3^−^ regulatory CD4^+^ T cells characterized by high IL‐10 production and expression of inhibitory receptors, typically induced under conditions of chronic antigen exposure.^30^, ^31^ Their differentiation depends on Eomes, which promotes IL‐10 expression and constrains alternative T‐helper lineage programs.^32^ Recent work in B‐ALL demonstrated that leukemic blasts can actively polarize tumor‐reactive TIM3^+^ CD4^+^ T cells toward a Tr1 phenotype, suppressing CD8^+^ T‐cell proliferation and correlating with poor clinical outcome.^33^ These findings position Tr1 differentiation as an active immune‐evasion strategy rather than a passive consequence of immune dysfunction. Importantly, Tr1 cells exhibit considerable functional plasticity. In CLL, IL‐10–producing, Eomes‐dependent Tr1 cells can exert cytotoxic activity and contribute to immune control,^34^ whereas vaccination studies have shown that cytotoxic Tr1 cells induced by high‐dose MHC II neoantigens suppress antitumor immunity by selectively eliminating antigen‐presenting cDC1s, thereby impairing T‐cell priming and checkpoint blockade efficacy.^35^ Thus, Tr1 cells can either enhance or inhibit tumor rejection depending on context. In the CLL mouse model, the accumulation of Tr1‐like cells alongside Eomes^+^ FoxP3^+^ CD4^+^ T cells suggests that chronic inflammation promotes a broader Eomes‐driven regulatory program within the CD4^+^ compartment.

The identification of CD4^+^ T cells co‐expressing FoxP3 and Eomes adds further complexity to regulatory T‐cell plasticity. Traditionally, FoxP3 and Eomes have been considered mutually exclusive transcriptional programs in CD4^+^ T cells, with Eomes expression limiting FoxP3 induction and stabilization.^36^ Indeed, loss of Eomes has been associated with enhanced accumulation of conventional FoxP3^+^ Tregs, supporting the concept that Eomes constrains terminal Treg differentiation. However, emerging evidence indicates that FoxP3^+^ Eomes^+^ CD4^+^ T cells may represent a transient, plastic, intermediate state rather than a stable lineage. Such double‐positive cells may reside at the intersection of Treg and Tr1 differentiation pathways, retaining the potential to adopt suppressive or effector‐like fates in response to contextual cues. Notably, FoxP3^+^ Eomes^+^ CD4^+^ T cells have been shown to acquire cytotoxic functions and eliminate virus‐induced tumor cells following therapeutic co‐stimulation, demonstrating that tumor‐induced Tregs can be reprogrammed to restore antitumor immunity.^37^ In the context of CLL, accumulation of FoxP3^+^ Eomes^+^ T cells may therefore reflect an unstable regulatory state driven by chronic antigen exposure and inflammatory signals. Whether these cells preferentially mature into suppressive Tregs, transition toward Tr1‐like states, or retain latent cytotoxic potential remains an important question with therapeutic implications.

Given that IL‐10 signaling is central to Tr1 induction and function, and that immunosuppressive myeloid cells represent a major source of IL‐10 and other cytokines, it is plausible that myeloid–T‐cell cross‐talk reinforces both Tr1 and Eomes^+^ Treg differentiation, thereby accelerating T‐cell exhaustion and immune escape. Immunosuppressive myeloid cells, including PD‐L1^+^ patrolling monocytes and regulatory DC subsets, expand in our model and in other tumor contexts where they impede T‐cell activation and skew the local milieu toward anti‐inflammatory signaling. Suppressive myeloid cells have recently been recognized as central mediators of resistance to immunotherapies across cancers, capable of inhibiting T‐cell priming, altering checkpoint biology, and orchestrating recruitment and expansion of regulatory lymphocytes. There is further evidence that they prevent effective CAR T‐cell responses in B‐cell lymphoma.^27^ In this light, our observation of increased PD‐L1 expression on patrolling monocytes, neutrophils, cDC1, cDC2, and mregDCs, alongside Treg expansion and T‐cell exhaustion, suggests interconnected suppressive networks that evolve with CLL progression.

Tumor‐associated DCs frequently display a semi‐mature phenotype characterized by altered expression of antigen‐presentation and co‐stimulatory molecules. During CLL progression, cDC1s increased XCR1 expression, indicating terminal differentiation and elevated secretion of inflammatory cytokines and chemokines.^38^, ^39^, ^40^ However, their phenotype was characterized by low levels of MHC II, CD80/86, CD44, and ICOS‐L, with high expression of CD40, CD54, and PD‐L1, a semi‐mature profile indicative of incomplete maturation. In several tumor models, including KPC pancreatic cancer and MC‐38 colon carcinoma, cDC1 dysfunction is characterized by reduced MHC II and co‐stimulatory molecule expression, increased PD‐L1 and ICAM‐1, and impaired Th1/CD8 priming capacity, findings that align closely with our observations in the TCL1‐AT mouse model.^41^, ^42^ IL‐6–driven apoptosis and checkpoint upregulation further contribute to systemic cDC1 dysfunction in tumor‐bearing hosts.^43^ Similarly, tumor‐resident cDC2 and mregDC subsets have been described as hyper‐adhesive, PD‐L1^high^ populations with reduced migratory capacity and diminished ICOS‐L expression, favoring stable but exhausted T‐cell synapses and impaired Tfh/Th2 support.^44^ These phenotypes align with reports of “mregDC tumor‐resident” populations, characterized by high CD80/CD86 and PD‐L1 expression, reduced antigen presentation, and local retention within the TME.^45^ Functionally, such tolerogenic DC (tolDC) states promote regulatory and exhausted T‐cell dominance rather than effective effector responses. Bidirectional DC–Tr1 interactions further stabilize tolerogenic circuits: DC‐derived IL‐10, TGF‐β, and IL‐27 drive Tr1 differentiation, while Tr1 cells reciprocally condition DCs to produce IL‐10 and reduce co‐stimulation, establishing a feedback loop that reinforces peripheral tolerance during chronic antigen exposure.^46^, ^47^ Tumors exploit this DC–Tr1 axis to create local immunosuppression.^48^, ^49^ Collectively, the myeloid alterations observed in the TCL1‐AT model point to immune suppression, as evidenced by expansion of PD‐L1^high^ cells alongside reduced MHC class II expression, a combination likely to compromise antigen‐specific T‐cell activation. Altogether, this results in Treg/exhaustion dominance rather than effector T cells, as supported by our data on Tr1‐like cells and Tregs and corroborated by other studies.^50^ While low ICOS‐L reduces Treg expansion, recent data indicate that ICOS‐L on APCs is decreased through endocytosis when interacting with ICOS‐expressing T cells, potentially explaining the lowered ICOS‐L levels on DC subsets during CLL progression.^51^, ^52^, ^53^

A notable strength of our study is the longitudinal resolution of immune remodeling, which provides insight into the temporal sequence of events leading to immune escape. Rather than emerging simultaneously, suppressive immune populations accumulated in a stepwise manner during disease progression. Early stages were characterized by expansion of neutrophils, NK cells, and CD8^+^ effector T cells, suggesting an initial phase of innate and adaptive immune activation in response to the developing leukemia. This was followed by the appearance of Eomes^+^ Tregs, which expanded before the broader accumulation of suppressive immune populations. At later stages, Tr1‐like cells, PD‐L1^high^ patrolling monocytes, mregDCs, and exhausted CD8^+^ T cells increased in parallel, coinciding with the emergence of a fully established immunosuppressive microenvironment. These observations suggest that early regulatory populations, particularly Eomes^+^ Tregs, may contribute to the initiation of immune dysfunction and help orchestrate the subsequent development of suppressive myeloid and lymphoid networks. While the mechanisms driving this sequential remodeling remain to be defined, our findings support a model in which immune escape in CLL results from a progressive and coordinated evolution of regulatory circuits rather than the abrupt appearance of terminally exhausted immune states.

The combined presence of immunosuppressive cytokines, checkpoint ligands, and regulatory subsets likely limits the efficacy of conventional immune checkpoint inhibitors in CLL, a phenomenon reflected in clinical trials reporting low response rates to anti‐PD‐1/PD‐L1 therapies.^5^ From a translational perspective, identifying regulatory myeloid and lymphoid states highlights several opportunities for intervention. Selective modulation of suppressive myeloid populations, including PD‐L1^high^ patrolling monocytes and regulatory DCs, represents an attractive approach to disrupt immune tolerance and reinvigorate antitumor effector functions.^54^ Such combinatorial strategies, aimed at both restoring effector T/NK‐cell competence and alleviating suppressive circuits, may ultimately improve responses to immunotherapy and circumvent adaptive immune resistance in CLL.

Collectively, these findings underscore CD4^+^ T‐cell fate decisions and the immune‐suppressive polarization of the myeloid compartment as pivotal determinants of immune control in leukemia, suggesting that therapeutic strategies aimed at reprogramming regulatory CD4^+^ T cells and/or myeloid cells may represent a promising avenue for combination immunotherapies.

## AUTHOR CONTRIBUTIONS

**Hannah Briesch**: Conceptualization; writing—original draft; methodology; visualization; software; formal analysis; data curation; validation; investigation. **Mariana Coelho**: Conceptualization; investigation; methodology; formal analysis; writing—review and editing; data curation. **Alessia Floerchinger**: Investigation; formal analysis; software; visualization; writing—review and editing. **Yuliia Borysovych**: Writing—review and editing; formal analysis; methodology; investigation. **Nicolas Aubert**: Investigation; methodology; formal analysis; writing—review and editing; software. **Daniel Medina‐Gil**: Formal analysis; writing—review and editing; methodology; investigation. **Manina Breitinger**: Investigation; formal analysis; writing—review and editing. **Marc Zapatka**: Software; data curation; formal analysis. **Stella E. Autenrieth**: Writing—original draft; project administration; supervision; resources; methodology; software; formal analysis; funding acquisition. **Martina Seiffert**: Conceptualization; writing—original draft; project administration; resources; supervision; funding acquisition.

## CONFLICT OF INTEREST STATEMENT

The authors declare no conflicts of interest.

## ETHICS STATEMENT

All experiments involving laboratory animals were conducted in pathogen‐free animal facilities at the German Cancer Research Center in Heidelberg according to governmental and institutional guidelines and authorized by the local authorities (Regierungspräsidium Karlsruhe, permit numbers: G‐39/19, G‐112/21, and G‐230/23). Mice were treated following the European guidelines.

## FUNDING

This work was supported by the German José Carreras Foundation (project 03R/2021). H.B., M.C., A.F., and Y.B. were funded with a DKFZ International PhD Program Fellowship. Open Access funding enabled and organized by Projekt DEAL.

## Supporting information

Supporting Information.

Supporting Information.

Supporting Information.

## Data Availability

The data that support the findings of this study are available from the corresponding author upon reasonable request.

## References

[1] Jain N , Wierda WG , O'Brien S . Chronic lymphocytic leukaemia. Lancet. 2024;404(10453):694‐706. 39068951 10.1016/S0140-6736(24)00595-6

[2] Koehrer S , Burger JA . Chronic lymphocytic leukemia: disease biology. Acta Haematol. 2024;147(1):8‐21. 37717577 10.1159/000533610 PMC11753505

[3] van Attekum MH , Eldering E , Kater AP . Chronic lymphocytic leukemia cells are active participants in microenvironmental cross‐talk. Haematologica. 2017;102(9):1469‐1476. 28775118 10.3324/haematol.2016.142679 PMC5685246

[4] Nadeu F , Royo R , Massoni‐Badosa R , et al. Detection of early seeding of Richter transformation in chronic lymphocytic leukemia. Nat Med. 2022;28(8):1662‐1671. 35953718 10.1038/s41591-022-01927-8 PMC9388377

[5] Ding W , LaPlant BR , Call TG , et al. Pembrolizumab in patients with CLL and Richter transformation or with relapsed CLL. Blood. 2017;129(26):3419‐3427. 28424162 10.1182/blood-2017-02-765685 PMC5492091

[6] Fraietta JA , Lacey SF , Orlando EJ , et al. Determinants of response and resistance to CD19 chimeric antigen receptor (CAR) T cell therapy of chronic lymphocytic leukemia. Nat Med. 2018;24(5):563‐571. 29713085 10.1038/s41591-018-0010-1 PMC6117613

[7] Nicholas NS , Apollonio B , Ramsay AG . Tumor microenvironment (TME)‐driven immune suppression in B cell malignancy. Biochim Biophys Acta Mol Cell Res. 2016;1863(3):471‐482. 10.1016/j.bbamcr.2015.11.003 26554850

[8] Llaó‐Cid L , Wong J , Fernandez Botana I , et al. Integrative multi‐omics reveals a regulatory and exhausted T‐cell landscape in CLL and identifies galectin‐9 as an immunotherapy target. Nat Commun. 2025;16(1):7271. 40775219 10.1038/s41467-025-61822-x PMC12331977

[9] Jitschin R , Braun M , Büttner M , et al. CLL‐cells induce IDOhi CD14^+^HLA‐DRlo myeloid‐derived suppressor cells that inhibit T‐cell responses and promote TRegs. Blood. 2014;124(5):750‐760. 24850760 10.1182/blood-2013-12-546416

[10] Riches JC , Davies JK , McClanahan F , et al. T cells from CLL patients exhibit features of T‐cell exhaustion but retain capacity for cytokine production. Blood. 2013;121(9):1612‐1621. 23247726 10.1182/blood-2012-09-457531 PMC3587324

[11] Wierz M , Pierson S , Guyonnet L , et al. Dual PD1/LAG3 immune checkpoint blockade limits tumor development in a murine model of chronic lymphocytic leukemia. Blood. 2018;131(14):1617‐1621. 29439955 10.1182/blood-2017-06-792267 PMC5887766

[12] Sun C , Chen YC , Martinez Zurita A , et al. The immune microenvironment shapes transcriptional and genetic heterogeneity in chronic lymphocytic leukemia. Blood Adv. 2023;7(1):145‐158. 35358998 10.1182/bloodadvances.2021006941 PMC9811214

[13] Roider T , Baertsch MA , Fitzgerald D , et al. Multimodal and spatially resolved profiling identifies distinct patterns of T cell infiltration in nodal B cell lymphoma entities. Nat Cell Biol. 2024;26(3):478‐489. 38379051 10.1038/s41556-024-01358-2 PMC10940160

[14] Floerchinger A , Seiffert M . Lessons learned from the Eµ‐TCL1 mouse model of CLL. Semin Hematol. 2024;61(3):194‐200. 38839457 10.1053/j.seminhematol.2024.05.002

[15] Brinkmann BJ , Floerchinger A , Schniederjohann C , et al. CD20‐bispecific antibodies improve response to CD19‐CAR T‐cells in lymphoma in‐vitro and CLL in‐vivo models. Blood. 2024;144(7):784‐789. 38805637 10.1182/blood.2023022682

[16] Bichi R , Shinton SA , Martin ES , et al. Human chronic lymphocytic leukemia modeled in mouse by targeted TCL1 expression. Proc Natl Acad Sci USA. 2002;99(10):6955‐6960. 12011454 10.1073/pnas.102181599 PMC124510

[17] Nunes C , Wong R , Mason M , Fegan C , Man S , Pepper C . Expansion of a CD8(+)PD‐1(+) replicative senescence phenotype in early stage CLL patients is associated with inverted CD4:CD8 ratios and disease progression. Clin Cancer Res. 2012;18(3):678‐687. 22190592 10.1158/1078-0432.CCR-11-2630

[18] Hanna BS , Llaó‐Cid L , Iskar M , et al. Interleukin‐10 receptor signaling promotes the maintenance of a PD‐1int TCF‐1^+^ CD8^+^ T cell population that sustains anti‐tumor immunity. Immunity. 2021;54(12):2825‐2841. 34879221 10.1016/j.immuni.2021.11.004

[19] McClanahan F , Riches JC , Miller S , et al. Mechanisms of PD‐L1/PD‐1‐mediated CD8 T‐cell dysfunction in the context of aging‐related immune defects in the Eµ‐TCL1 CLL mouse model. Blood. 2015;126(2):212‐221. 25979947 10.1182/blood-2015-02-626754 PMC4497962

[20] Hanna BS , Roessner PM , Yazdanparast H , et al. Control of chronic lymphocytic leukemia development by clonally‐expanded CD8+ T‐cells that undergo functional exhaustion in secondary lymphoid tissues. Leukemia. 2019;33(3):625‐637. 30267008 10.1038/s41375-018-0250-6

[21] Levine JH , Simonds EF , Bendall SC , et al. Data‐driven phenotypic dissection of AML reveals progenitor‐like cells that correlate with prognosis. Cell. 2015;162(1):184‐197. 26095251 10.1016/j.cell.2015.05.047 PMC4508757

[22] McInnes L , Healy J , Saul N , Großberger L . UMAP: Uniform Manifold Approximation and Projection. J Open Source Softw. 2018;3(29):861.

[23] Maier B , Leader AM , Chen ST , et al. A conserved dendritic‐cell regulatory program limits antitumour immunity. Nature. 2020;580(7802):257‐262. 32269339 10.1038/s41586-020-2134-y PMC7787191

[24] Zilionis R , Engblom C , Pfirschke C , et al. Single‐cell transcriptomics of human and mouse lung cancers reveals conserved myeloid populations across individuals and species. Immunity. 2019;50(5):1317‐1334.e1310. 30979687 10.1016/j.immuni.2019.03.009 PMC6620049

[25] Zhang Q , He Y , Luo N , et al. Landscape and dynamics of single immune cells in hepatocellular carcinoma. Cell. 2019;179(4):829‐845.e820. 31675496 10.1016/j.cell.2019.10.003

[26] Gerhard GM , Bill R , Messemaker M , Klein AM , Pittet MJ . Tumor‐infiltrating dendritic cell states are conserved across solid human cancers. J Exp Med. 2021;218(1):e20200264. 33601412 10.1084/jem.20200264 PMC7754678

[27] Stahl D , Gödel P , Balke‐Want H , et al. CSF1R(+) myeloid‐monocytic cells drive CAR‐T cell resistance in aggressive B cell lymphoma. Cancer Cell. 2025;43(8):1476‐1494.e1410. 40513575 10.1016/j.ccell.2025.05.013

[28] Hanna BS , McClanahan F , Yazdanparast H , et al. Depletion of CLL‐associated patrolling monocytes and macrophages controls disease development and repairs immune dysfunction in vivo. Leukemia. 2016;30(3):570‐579. 26522085 10.1038/leu.2015.305

[29] Groux H , O'Garra A , Bigler M , et al. A CD4^+^ T‐cell subset inhibits antigen‐specific T‐cell responses and prevents colitis. Nature. 1997;389(6652):737‐742. 9338786 10.1038/39614

[30] Roncarolo MG , Gregori S , Bacchetta R , Battaglia M , Gagliani N . The biology of T regulatory type 1 cells and their therapeutic application in immune‐mediated diseases. Immunity. 2018;49(6):1004‐1019. 30566879 10.1016/j.immuni.2018.12.001

[31] Vieira PL , Christensen JR , Minaee S , et al. IL‐10‐secreting regulatory T cells do not express Foxp3 but have comparable regulatory function to naturally occurring CD4^+^CD25^+^ regulatory T cells. J Immunol. 2004;172(10):5986‐5993. 15128781 10.4049/jimmunol.172.10.5986

[32] Zhang P , Lee JS , Gartlan KH , et al. Eomesodermin promotes the development of type 1 regulatory T (T(R)1) cells. Sci Immunol. 2017;2(10):eaah7152. 28738016 10.1126/sciimmunol.aah7152 PMC5714294

[33] Venkatesh H , Granados Centeno E , Luo Q , et al. Leukemia escapes immunity by imposing a type 1 regulatory program on neoantigen‐specific CD4^+^ T cells. Blood. 2025;146(23):2779‐2793. 40906918 10.1182/blood.2024028194 PMC12983000

[34] Roessner PM , Llao Cid L , Lupar E , et al. EOMES and IL‐10 regulate antitumor activity of T regulatory type 1 CD4(+) T cells in chronic lymphocytic leukemia. Leukemia. 2021;35(8):2311‐2324. 33526861 10.1038/s41375-021-01136-1 PMC8324479

[35] Sultan H , Takeuchi Y , Ward JP , et al. Neoantigen‐specific cytotoxic Tr1 CD4 T cells suppress cancer immunotherapy. Nature. 2024;632(8023):182‐191. 39048822 10.1038/s41586-024-07752-y PMC11291290

[36] Lupar E , Brack M , Garnier L , et al. Eomesodermin expression in CD4^+^ T cells restricts peripheral Foxp3 induction. J Immunol. 2015;195(10):4742‐4752. 26453746 10.4049/jimmunol.1501159

[37] Akhmetzyanova I , Zelinskyy G , Littwitz‐Salomon E , et al. CD137 agonist therapy can reprogram regulatory T cells into cytotoxic CD4^+^ T cells with antitumor activity. J Immunol. 2016;196(1):484‐492. 26608920 10.4049/jimmunol.1403039

[38] Yamazaki C , Sugiyama M , Ohta T , et al. Critical roles of a dendritic cell subset expressing a chemokine receptor, XCR1. J Immunol. 2013;190(12):6071‐6082. 23670193 10.4049/jimmunol.1202798

[39] Dorner BG , Dorner MB , Zhou X , et al. Selective expression of the chemokine receptor XCR1 on cross‐presenting dendritic cells determines cooperation with CD8^+^ T cells. Immunity. 2009;31(5):823‐833. 19913446 10.1016/j.immuni.2009.08.027

[40] Heger L , Hatscher L , Liang C , et al. XCR1 expression distinguishes human conventional dendritic cell type 1 with full effector functions from their immediate precursors. Proc Natl Acad Sci. 2023;120(33):e2300343120. 37566635 10.1073/pnas.2300343120 PMC10438835

[41] Lin JH , Huffman AP , Wattenberg MM , et al. Type 1 conventional dendritic cells are systemically dysregulated early in pancreatic carcinogenesis. J Exp Med. 2020;217(8):e20190673. 32453421 10.1084/jem.20190673 PMC7398166

[42] Sapoznikov A , Kozlovski S , Levi N , et al. Dendritic cell ICAM‐1 strengthens synapses with CD8 T cells but is not required for their early differentiation. Cell Rep. 2023;42(8):112864. 37494182 10.1016/j.celrep.2023.112864

[43] Peng Q , Qiu X , Zhang Z , et al. PD‐L1 on dendritic cells attenuates T cell activation and regulates response to immune checkpoint blockade. Nat Commun. 2020;11(1):4835. 32973173 10.1038/s41467-020-18570-x PMC7518441

[44] Li J , Zhou J , Huang H , Jiang J , Zhang T , Ni C . Mature dendritic cells enriched in immunoregulatory molecules (mregDCs): a novel population in the tumour microenvironment and immunotherapy target. Clin Transl Med. 2023;13(2):e1199. 36808888 10.1002/ctm2.1199 PMC9937888

[45] Lee CYC , Kennedy BC , Richoz N , et al. Tumour‐retained activated CCR7(+) dendritic cells are heterogeneous and regulate local anti‐tumour cytolytic activity. Nat Commun. 2024;15(1):682. 38267413 10.1038/s41467-024-44787-1 PMC10808534

[46] Wakkach A , Fournier N , Brun V , Breittmayer JP , Cottrez F , Groux H . Characterization of dendritic cells that induce tolerance and T regulatory 1 cell differentiation in vivo. Immunity. 2003;18(5):605‐617. 12753738 10.1016/s1074-7613(03)00113-4

[47] Ness S , Lin S , Gordon JR . Regulatory dendritic cells, T cell tolerance, and dendritic cell therapy for immunologic disease. Front Immunol. 2021;12:633436. 33777019 10.3389/fimmu.2021.633436 PMC7988082

[48] Zeng H , Zhang R , Jin B , Chen L . Type 1 regulatory T cells: a new mechanism of peripheral immune tolerance. Cell Mol Immunol. 2015;12(5):566‐571. 26051475 10.1038/cmi.2015.44 PMC4579656

[49] Janikashvili N , Bonnotte B , Katsanis E , Larmonier N . The dendritic cell‐regulatory T lymphocyte crosstalk contributes to tumor‐induced tolerance. Clin Dev Immunol. 2011;2011:430394. 22110524 10.1155/2011/430394 PMC3216392

[50] Liu Q , Lu JY , Wang XH , Qu BJ , Li SR , Kang JR . Changes in the PD‐1 and PD‐L1 expressions of splenic dendritic cells in multiple‐organ dysfunction syndrome mice and their significance. Genet Mol Res. 2014;13(3):7666‐7672. 25299080 10.4238/2014.September.26.4

[51] Watanabe M , Takagi Y , Kotani M , et al. Down‐regulation of ICOS ligand by interaction with ICOS functions as a regulatory mechanism for immune responses. J Immunol. 2008;180(8):5222‐5234. 18390703 10.4049/jimmunol.180.8.5222

[52] Aragoneses‐Fenoll L , Montes‐Casado M , Ojeda G , et al. Role of endocytosis and trans‐endocytosis in ICOS costimulator‐induced downmodulation of the ICOS Ligand. J Leukoc Biol. 2021;110(5):867‐884. 33527556 10.1002/JLB.2A0220-127R PMC8597029

[53] Landuyt AE , Klocke BJ , Colvin TB , Schoeb TR , Maynard CL . Cutting edge: ICOS‐deficient regulatory T cells display normal induction of Il10 but readily downregulate expression of Foxp3. J Immunol. 2019;202(4):1039‐1044. 30642977 10.4049/jimmunol.1801266 PMC6363853

[54] Lei X , Wang Y , Broens C , Borst J , Xiao Y . Immune checkpoints targeting dendritic cells for antibody‐based modulation in cancer. Int Rev Cell Mol Biol. 2024;382:145‐179. 38225102 10.1016/bs.ircmb.2023.07.006

