# Supplementary material for: High‐dimensional spectral flow cytometry uncovers progressive T‐cell exhaustion and myeloid cell reprogramming in a CLL mouse model
Source: Hemasphere. 2026 Sep 14;10(9):e70476. doi: 10.1002/hem3.70476 (PMC13573940; doi:10.1002/hem3.70476)
Supplement: Supplementary file 1 [file HEM3-10-e70476-s001.pdf]

Supporting Information S3: Supplementary Figures

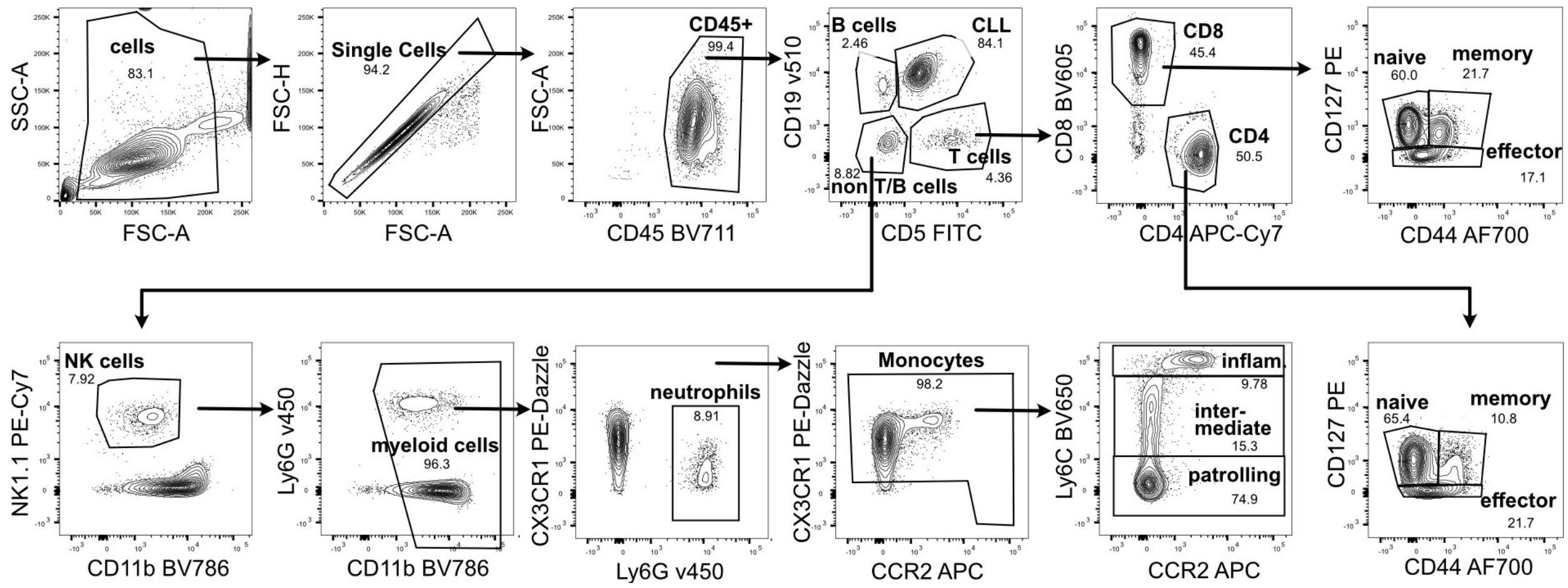

Supp. Fig. 1: Gating strategy for conventional flow cytometry-based analysis of CLL, T, NK and myeloid cells in blood

Representative gating strategy for conventional flow cytometry-based analysis of CLL cells, $CD4^+$ T cells, $CD8^+$ T cells, NK cells, neutrophils and monocytes in blood.

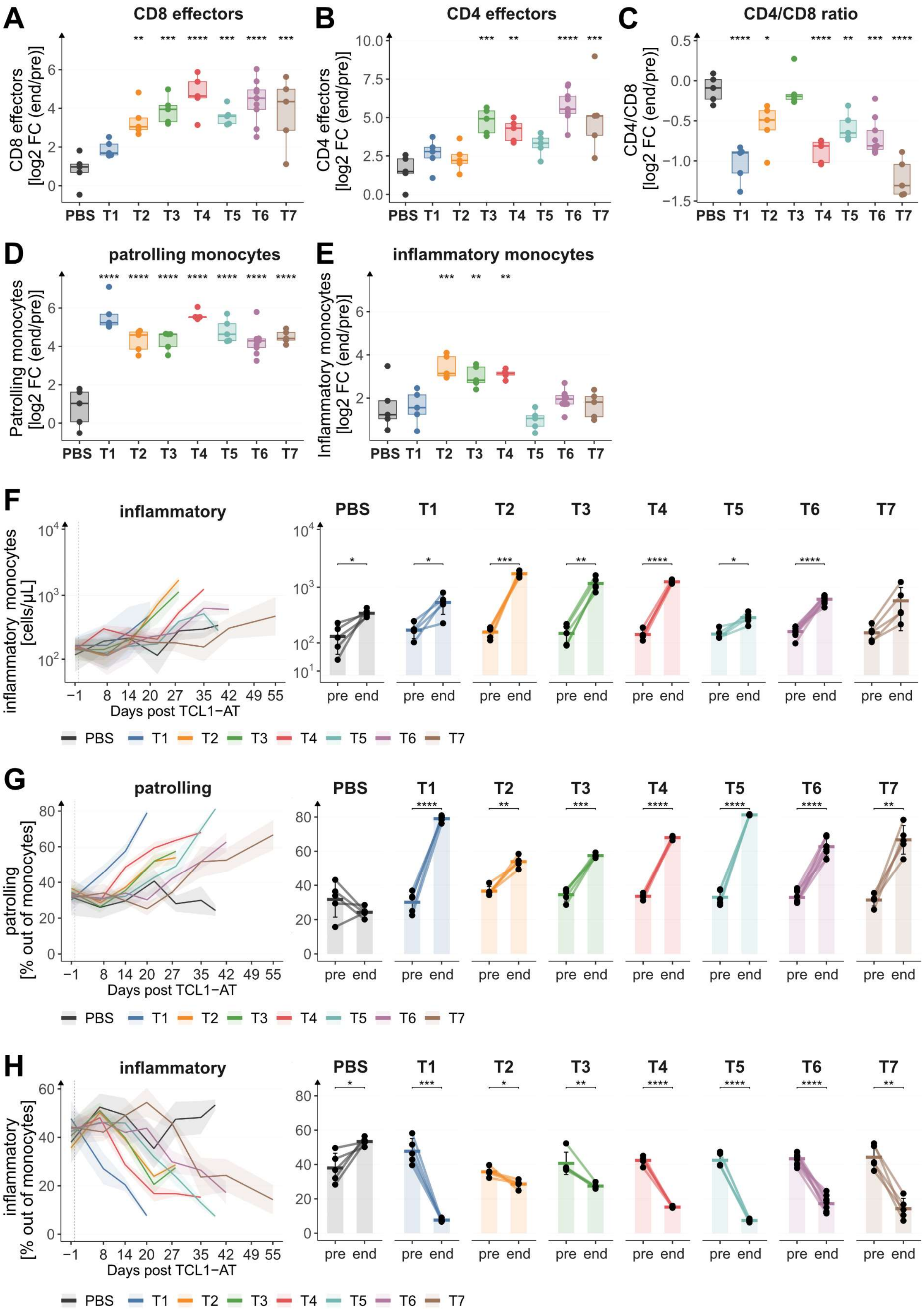
A
CD8 effectors
CD8 effectors [log2 FC (end/pre)]
B
CD4 effectors
CD4 effectors [log2 FC (end/pre)]
C
CD4/CD8 ratio
CD4/CD8 [log2 FC (end/pre)]
D
patrolling monocytes
Patrolling monocytes [log2 FC (end/pre)]
E
inflammatory monocytes
Inflammatory monocytes [log2 FC (end/pre)]
PBS T1 T2 T3 T4 T5 T6 T7
F
inflammatory
inflammatory monocytes [cells/µL]
Days post TCL1−AT
pre end
G
patrolling
patrolling [% out of monocytes]
Days post TCL1−AT
pre end
H
inflammatory
inflammatory [% out of monocytes]
Days post TCL1−AT
pre end
PBS T1 T2 T3 T4 T5 T6 T7

Supp. Fig. 2: Additional analysis of T cell and monocyte subsets in blood during CLL development

Supporting quantifications related to Figure 1. Conventional flow cytometry-based analysis of T cells myeloid cells in peripheral blood from mice after seven independent TCL1 adoptive transfer (AT) experiments (T1-T7). A-E) Log_2 fold change (FC) in $CD8^+$ effector counts (A), $CD4^+$ effector counts (B), CD4/CD8 ratio (C), patrolling monocyte counts (D) and inflammatory monocyte counts (E), comparing the last blood withdrawal performed 0-5 days before euthanasia upon reaching endpoint criteria (end) with one day before AT (pre). The exact timepoints of blood withdrawals and euthanasia for each of the seven TCL1-AT experiments are provided in Supplementary Table 1. F-H) Absolute counts of Inflammatory monocytes (F), and frequencies of patrolling (G) and inflammatory monocytes (H) in peripheral blood were quantified over time (left panels). Lines represent the mean, and shaded areas indicate the standard deviation (SD). Right panels show paired comparisons pre and end timepoint, as indicated above. A-F) Box plots indicate medians with interquartile ranges (whiskers to 1.5×IQR). One-way ANOVA followed by Dunnett's multiple comparisons test (each group vs. PBS control). F-H) Bar plots indicate mean with SD, paired measurement within the same mice are indicated by connected line. Statistical significance between 1 day before AT and last timepoint was assessed within each group using paired two-tailed Student’s t-tests with Bonferroni-Holm correction for multiple testing. ∗∗∗∗$p < 0.0001$, ∗∗∗$p \leq 0.001$, ∗∗$p \leq 0.01$, ∗$p \leq 0.05$.

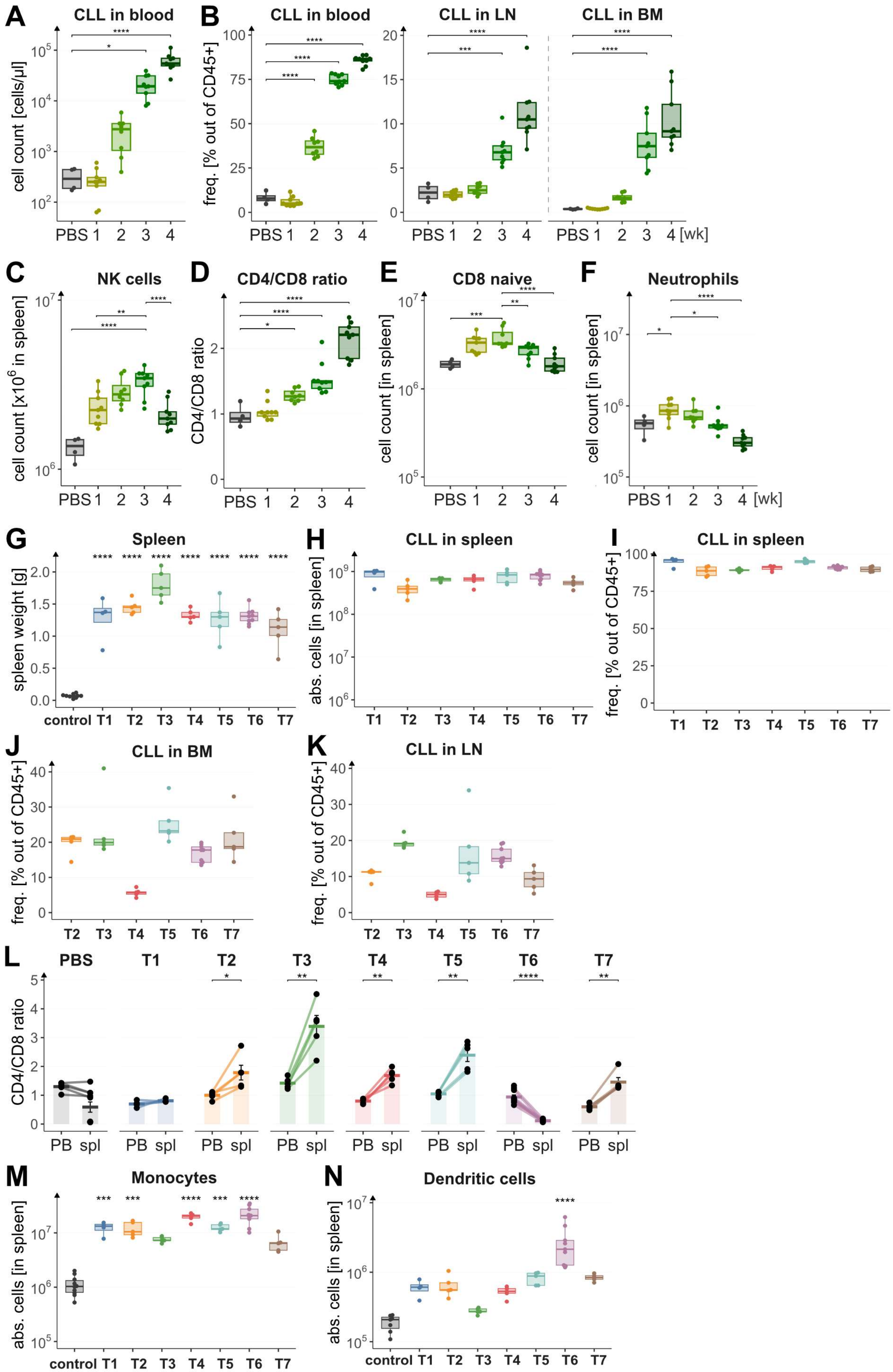
A
CLL in blood
cell count [cells/µl]
PBS 1 2 3 4
B
CLL in blood
freq. [% out of CD45+]
CLL in LN
CLL in BM
PBS 1 2 3 4 [wk]
C
NK cells
cell count [x10^6 in spleen]
D
CD4/CD8 ratio
CD4/CD8 ratio
E
CD8 naive
cell count [in spleen]
F
Neutrophils
cell count [in spleen]
PBS 1 2 3 4 [wk]
G
Spleen
spleen weight [g]
control T1 T2 T3 T4 T5 T6 T7
H
CLL in spleen
abs. cells [in spleen]
T1 T2 T3 T4 T5 T6 T7
I
CLL in spleen
freq. [% out of CD45+]
T1 T2 T3 T4 T5 T6 T7
J
CLL in BM
freq. [% out of CD45+]
T2 T3 T4 T5 T6 T7
K
CLL in LN
freq. [% out of CD45+]
T2 T3 T4 T5 T6 T7
L
PBS T1 T2 T3 T4 T5 T6 T7
CD4/CD8 ratio
PB spl
M
Monocytes
abs. cells [in spleen]
control T1 T2 T3 T4 T5 T6 T7
N
Dendritic cells
abs. cells [in spleen]
control T1 T2 T3 T4 T5 T6 T7

Supp. Fig. 3: Conventional flow cytometry-based analysis of CLL or immune cell types in blood, BM, LN and spleen

A-F) Conventional flow cytometry-based analysis of TCL1 CLL, T, NK, and myeloid cells in mice before (PBS) and after 1-4 weeks post TCL1-AT, supporting quantifications related to Figure 2 A) Absolute CLL cell count in blood (in cells/µl). B) Frequencies of CLL cells out of $CD45^+$ cells in blood (left), lymph node (LN, middle), and bone marrow (BM, right). C) Absolute cell counts of NK cells in spleen. Same data as in Fig. 1E; one-way ANOVA followed by Dunnett's multiple comparisons test was performed for all groups vs. 3 weeks after TCL1-AT. D) CD4/CD8 ratio over time, which was calculated as the percentage of $CD4^+$ T cells divided by the percentage of $CD8^+$ T cells within T cells. E) Absolute cell counts of naïve $CD8^+$ T cells in spleen. Same data as in Fig. 1G; one-way ANOVA followed by Dunnett's multiple comparisons test was performed for all groups vs. 2 weeks after TCL1-AT. F) Absolute cell counts of neutrophils in the spleen. Same data as in Fig. 1I; one-way ANOVA followed by Dunnett's multiple comparisons test was performed for all groups vs. 1 week after TCL1-AT. G-N) Conventional flow cytometry-based analysis of CLL and immune cell populations in spleen, bone marrow (BM), and lymph node (LN) from mice after seven independent TCL1 adoptive transfer (AT) experiments (T1-T7). G) Spleen weight in gram (g). H) Absolute CLL counts in spleen. I-K) Frequency of CLL cells out of $CD45^+$ cells in spleen (I), BM (J) and LN (K). L) Paired comparison of CD4/CD8 ration in spleen (spl) with peripheral blood (PB) at end-stage disease. M-N) Absolute cell counts of monocytes (M) and dendritic cells (N) in spleen at end-stage disease. A-K, M-N) Box plots indicate medians with interquartile ranges (whiskers to 1.5×IQR). One-way ANOVA followed by Dunnett's multiple comparisons test (if not specified otherwise, each group vs. PBS control). L) Bar plots indicate mean with SD, paired measurement within the same mice are indicated by connected line. Statistical significance between spl and PB at end-stage disease was assessed within each group using paired two-tailed Student’s t-tests with Bonferroni-Holm correction for multiple testing. A-N) ****$p < 0.0001$, ***$p \leq 0.001$, **$p \leq 0.01$, *$p \leq 0.05$.

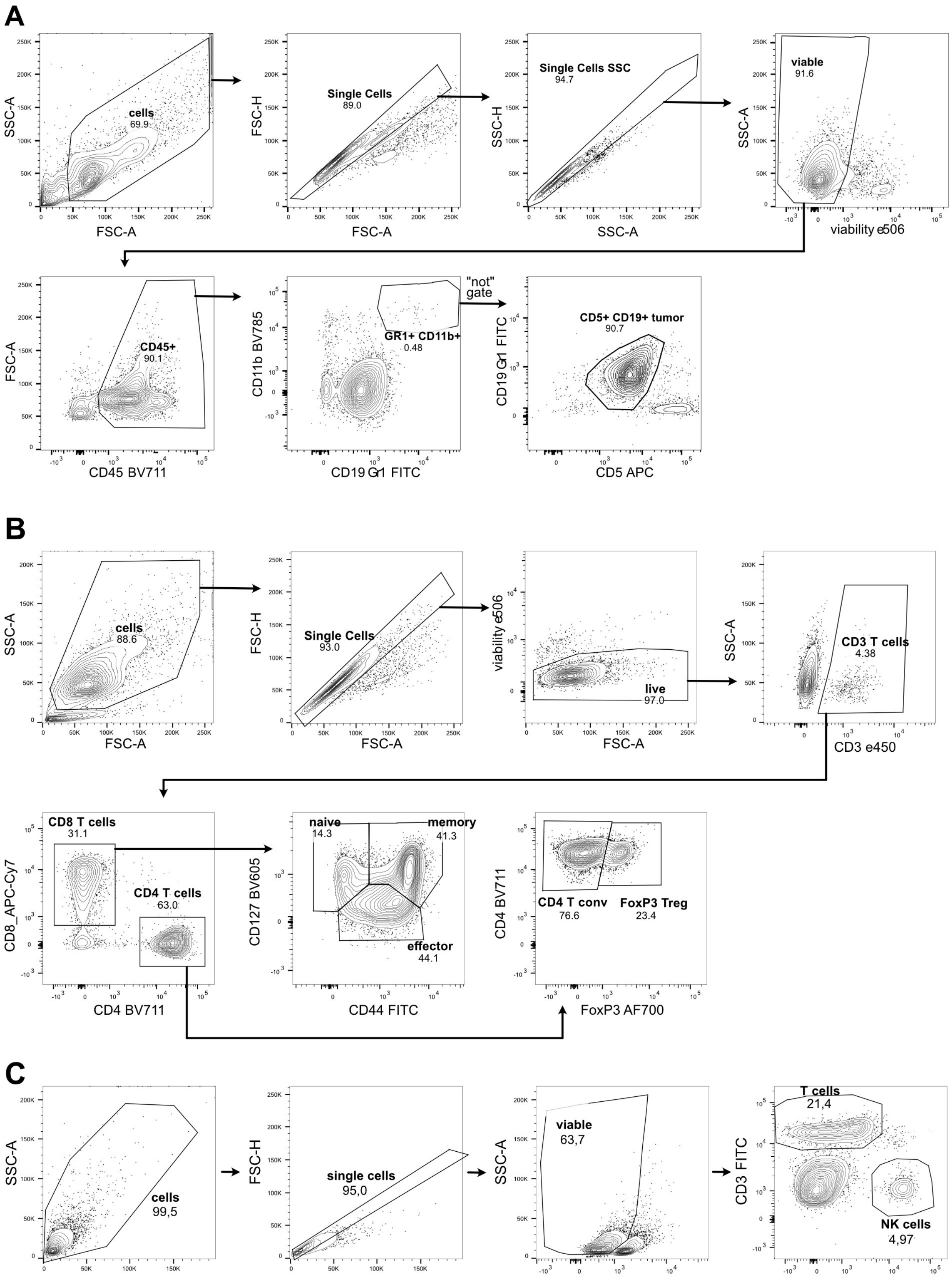

Supp. Fig. 4: Gating strategy for conventional flow cytometry-based analysis of CLL, T and NK cells

A-C) Representative gating strategy for conventional flow cytometry-based analysis of CLL cells (A), $CD4^+$ conventional T cells, $CD4^+$ Tregs, naïve, memory, and effector $CD8^+$ T cells (B) and NK cells (C) in spleen.

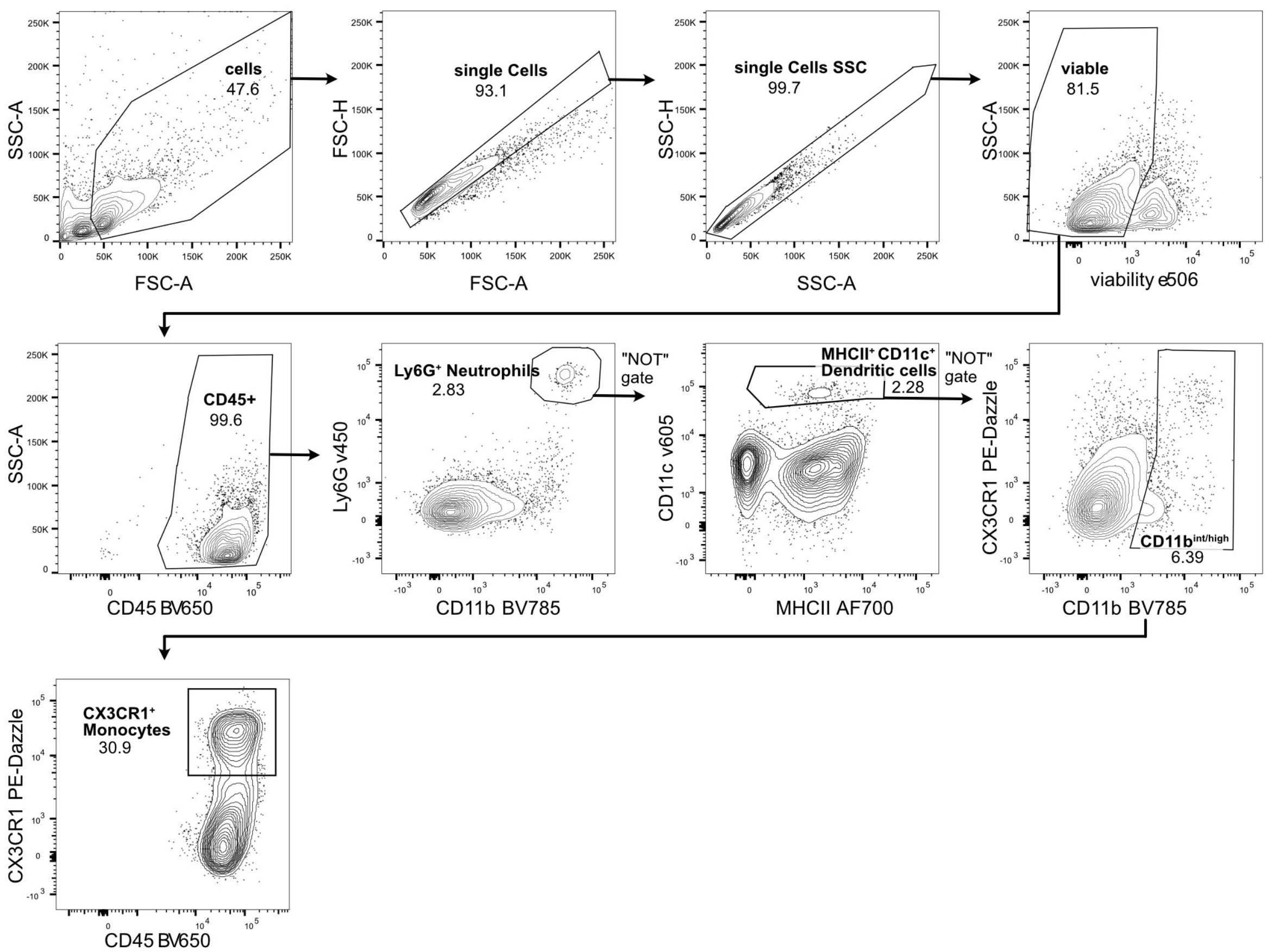

Supp. Fig. 5: Gating strategy for conventional flow cytometry-based analysis of myeloid cells

Representative gating strategy for conventional flow cytometry-based analysis of neutrophils, monocytes, and dendritic cells in spleen.

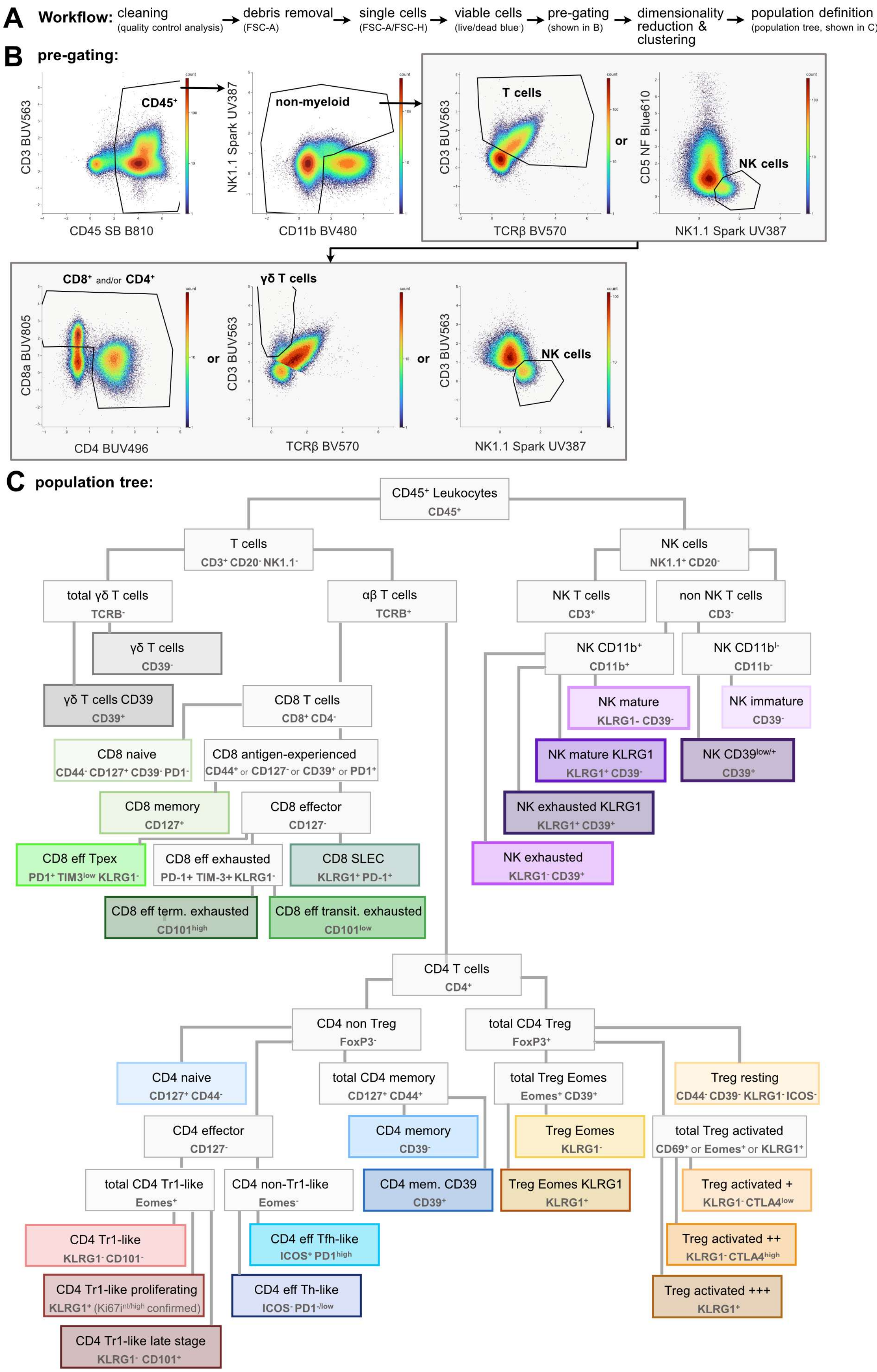
A Workflow: cleaning (quality control analysis) → debris removal (FSC-A) → single cells (FSC-A/FSC-H) → viable cells (live/dead blue-) → pre-gating (shown in B) → dimensionality reduction & clustering → population definition (population tree, shown in C)
B pre-gating:
CD45+
CD3 BUV563
CD45 SB B810
non-myeloid
NK1.1 Spark UV387
CD11b BV480
T cells
CD3 BUV563
TCRβ BV570
or
NK cells
CD5 NF Blue610
NK1.1 Spark UV387
CD8+ and/or CD4+
CD8a BUV805
CD4 BUV496
or
γδ T cells
CD3 BUV563
TCRβ BV570
or
NK cells
CD3 BUV563
NK1.1 Spark UV387
C population tree:
CD45+ Leukocytes CD45+
T cells CD3+ CD20- NK1.1-
NK cells NK1.1+ CD20-
total γδ T cells TCRB-
αβ T cells TCRB+
NK T cells CD3+
non NK T cells CD3-
γδ T cells CD39-
γδ T cells CD39 CD39+
NK CD11b+ CD11b+
NK CD11bl- CD11b-
CD8 T cells CD8+ CD4-
NK mature KLRG1- CD39-
NK immature CD39-
CD8 naive CD44- CD127+ CD39- PD1-
CD8 antigen-experienced CD44+ or CD127- or CD39+ or PD1+
NK mature KLRG1 KLRG1+ CD39-
NK CD39low/+ CD39+
CD8 memory CD127+
CD8 effector CD127-
NK exhausted KLRG1 KLRG1+ CD39+
NK exhausted KLRG1- CD39+
CD8 eff Tpex PD1+ TIM3low KLRG1-
CD8 eff exhausted PD-1+ TIM-3+ KLRG1-
CD8 SLEC KLRG1+ PD-1+
CD8 eff term. exhausted CD101high
CD8 eff transit. exhausted CD101low
CD4 T cells CD4+
CD4 non Treg FoxP3-
total CD4 Treg FoxP3+
CD4 naive CD127+ CD44-
total CD4 memory CD127+ CD44+
total Treg Eomes Eomes+ CD39+
Treg resting CD44- CD39- KLRG1- ICOS-
CD4 effector CD127-
CD4 memory CD39-
Treg Eomes KLRG1-
total Treg activated CD69+ or Eomes+ or KLRG1+
total CD4 Tr1-like Eomes+
CD4 non-Tr1-like Eomes-
CD4 mem. CD39 CD39+
Treg Eomes KLRG1 KLRG1+
Treg activated + KLRG1- CTLA4low
CD4 Tr1-like KLRG1- CD101-
CD4 eff Tfh-like ICOS+ PD1high
Treg activated ++ KLRG1- CTLA4high
CD4 Tr1-like proliferating KLRG1+ (Ki67int/high confirmed)
CD4 eff Th-like ICOS- PD1-/low
Treg activated +++ KLRG1+
CD4 Tr1-like late stage KLRG1- CD101+

Supp. Fig. 6: Workflow, pre-gating and population tree for the spectral flow cytometry-based analysis of T and NK cells

A) Workflow of spectral flow cytometry-based analysis of T and NK cells in the spleen. B) Pre-gating of $CD8^+$ and $CD4^+$ T cells, γδ T cells, and NK cells before performing dimensionality reduction. C) Hierarchical population tree (incl. marker expression) used for annotation of clusters via Cytolution Software. Colored rectangles represent the final populations annotated.

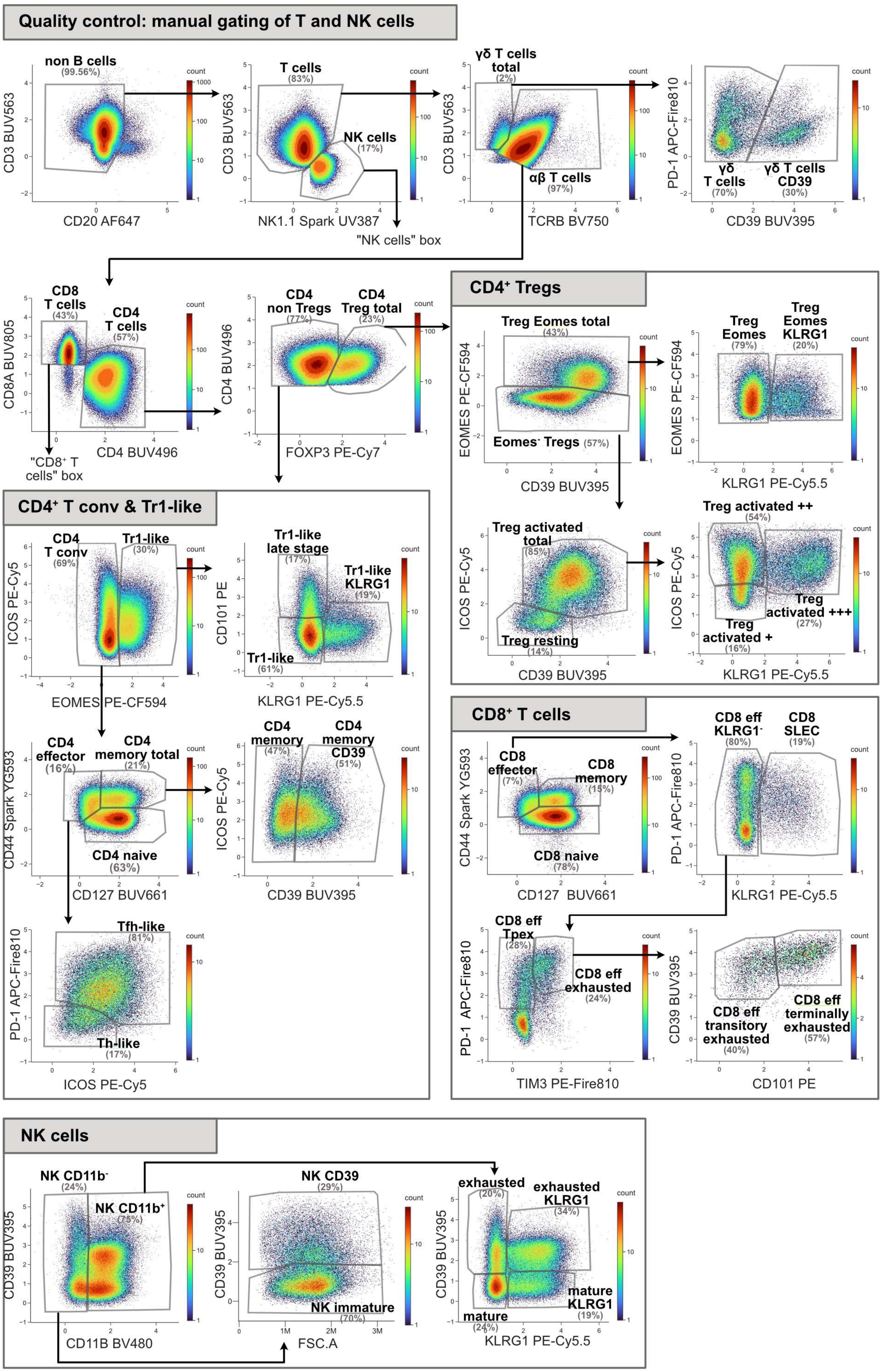

Quality control: manual gating of T and NK cells
non B cells
(99.56%)
CD3 BUV563
CD20 AF647
T cells
(83%)
NK cells
(17%)
NK1.1 Spark UV387
"NK cells" box
γδ T cells total
(2%)
αβ T cells
(97%)
TCRB BV750
PD-1 APC-Fire810
γδ T cells
(70%)
γδ T cells CD39
(30%)
CD39 BUV395
CD8 T cells
(43%)
CD4 T cells
(57%)
CD8A BUV805
CD4 BUV496
"CD8+ T cells" box
CD4 non Tregs
(77%)
CD4 Treg total
(23%)
FOXP3 PE-Cy7
CD4+ Tregs
Treg Eomes total
(43%)
Eomes- Tregs (57%)
EOMES PE-CF594
Treg Eomes
(79%)
Treg Eomes KLRG1
(20%)
KLRG1 PE-Cy5.5
Treg activated total
(85%)
Treg resting
(14%)
ICOS PE-Cy5
Treg activated ++
(54%)
Treg activated +++
(27%)
Treg activated +
(16%)
CD4+ T conv & Tr1-like
CD4 T conv
(69%)
Tr1-like
(30%)
EOMES PE-CF594
Tr1-like late stage
(17%)
Tr1-like KLRG1
(19%)
Tr1-like
(61%)
CD101 PE
CD4 effector
(16%)
CD4 memory total
(21%)
CD4 naive
(63%)
CD44 Spark YG593
CD127 BUV661
CD4 memory
(47%)
CD4 memory CD39
(51%)
Tfh-like
(81%)
Th-like
(17%)
CD8+ T cells
CD8 effector
(7%)
CD8 memory
(15%)
CD8 naive
(78%)
CD8 eff KLRG1-
(80%)
CD8 SLEC
(19%)
CD8 eff Tpex
(28%)
CD8 eff exhausted
(24%)
TIM3 PE-Fire810
CD8 eff transitory exhausted
(40%)
CD8 eff terminally exhausted
(57%)
CD101 PE
NK cells
NK CD11b-
(24%)
NK CD11b+
(75%)
CD11B BV480
NK CD39
(29%)
NK immature
(70%)
FSC.A
exhausted
(20%)
exhausted KLRG1
(34%)
mature
(24%)
mature KLRG1
(19%)
count

Supp. Fig. 7: Manual gating strategy of T and NK cell spectral flow cytometry panel

As a quality control for spectral flow cytometry analysis, a manual gating strategy for identifying the main cellular subsets, as described in the unsupervised data analysis, is presented. Arrows link related populations across plots. After pre-gating on $CD8^+$ and/or $CD4^+$ T cells or γδ T cells or NK cells (as shown in Supp. Fig. 4B), non-B cells were separated into $CD3^+$ T cells and $NK1.1^+$ NK cells, consistent with [1]. Within T cells, αβ and γδ T cells were identified by TCRβ expression. Total γδ T cells were subdivided into $CD39^+$ or $CD39^-$ subsets, based on [2-4]. Within αβ T cells, CD4 and CD8 served as markers for helper T cells and cytotoxic T cells, respectively. $CD4^+$ T cells were further categorized into non-Tregs and $FoxP3^+$ Tregs, which were separated into $Eomes^+$ or $Eomes^-$ Tregs. Total $Eomes^+$ Treg were categorized based on KLRG1 expression in Treg Eomes and Treg Eomes KLRG1. Additional markers, such as CD39, ICOS and KLRG1 enabled the classification of Tregs resting ($CD39^-$, $ICOS^{low}$), Tregs activated+ ($CD39^+$ $ICOS^{low/int}$ $KLRG1^-$), Tregs activated++ ($CD39^+$ $ICOS^{high}$ $KLRG1^-$), Tregs activated+++ ($CD39^+$ $ICOS^{high}$ $KLRG1^+$) within $Eomes^-$ Tregs, in accordance with [5-8] . Non-Tregs $CD4^+$ T cells were gated based on Eomes expression into $Eomes^+$ regulatory Type 1 (Tr1)-like cells and conventional (conv) $CD4^+$ T cells, in line with [9,10]. Based on CD44 and CD127 expression, naïve, memory, and effector T cells were defined in conv $CD4^+$ T cells [1,11,12]. Within the total $Eomes^+$ Tr1-like cells, KLRG1 and CD101 expression allowed the classification of Tr1-like $Eomes^+$ ($CD101^-$ $KLRG1^-$), Tr1-like $Eomes^+$ late stage ($CD101^+$ $KLRG1^{+/-}$) and Tr1-like $Eomes^+$ $KLRG1^+$ ($CD101^-$ $KLRG1^+$). Within memory $CD4^+$ T cells, $CD39^+$ and $CD39^-$ subsets were subgated. CD4 effectors were subdivided into T follicular helper (Tfh)-like and T helper (Th)-like cells based on PD1 and ICOS expression, according to [13]. The $CD8^+$ T cell compartment was divided into naïve, memory, and effector subsets based on CD44 and CD127 expression. The $CD8^+$ effectors were subgated into $KLRG1^+$ short-lived effector cells (SLEC) and $KLRG1^-$ $CD8^+$ effectors, consistent with [14]. Additional markers, such as PD-1, TIM-3, CD39 and CD101 enabled the classification of precursor exhausted (Tpex, $PD\text{-}1^+$ $TIM3^-$), transitory exhausted ($PD\text{-}1^+$ $TIM3^+$ $CD101^-$ $CD39^{int}$) and terminally exhausted ($PD\text{-}1^+$ $TIM3^+$ $CD101^+$ $CD39^{high}$) $CD8^+$ T cells, according to [15-19]. NK cells were first divided into different maturation statuses based on CD11b expression. Within the immature $CD11b^-$ NK subset, the $CD39^+$ and $CD39^-$ subsets were defined. According to the expression of CD39 and KLRG1 in the mature $CD11b^+$ NK cell subset, the following subsets were identified: mature ($CD39^-$ $KLRG1^-$), mature $KLRG1^+$ ($CD39^-$ $KLRG1^+$), exhausted ($CD39^+$ $KLRG1^-$), and exhausted $KLRG1^+$ ($CD39^+$ $KLRG1^+$), based on [18-23].The marker expression of annotated T and NK cell populations and corresponding literature referencesare also listed in Suppl. Table 2.

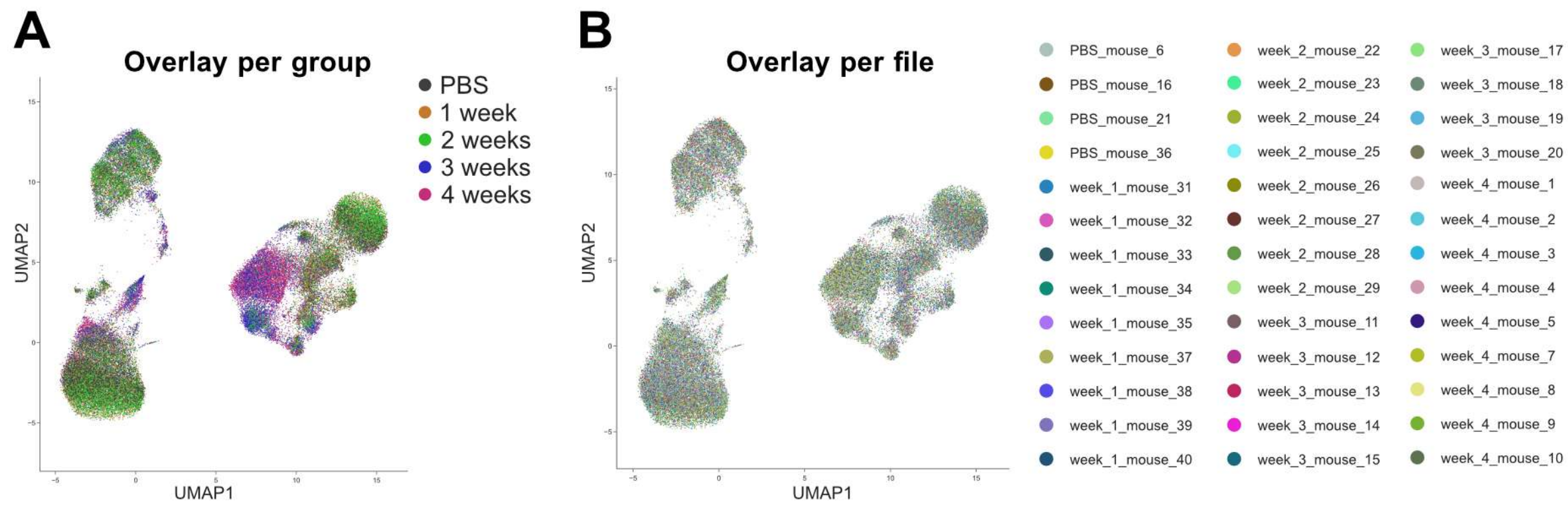

Supp. Fig. 8: UMAP projections of T/NK cell populations with overlaid groups and individual mice

UMAP projections of T and NK cell populations with overlaid groups (A, PBS control and 1-4 weeks post-AT) and all individual mice (B, n=39).

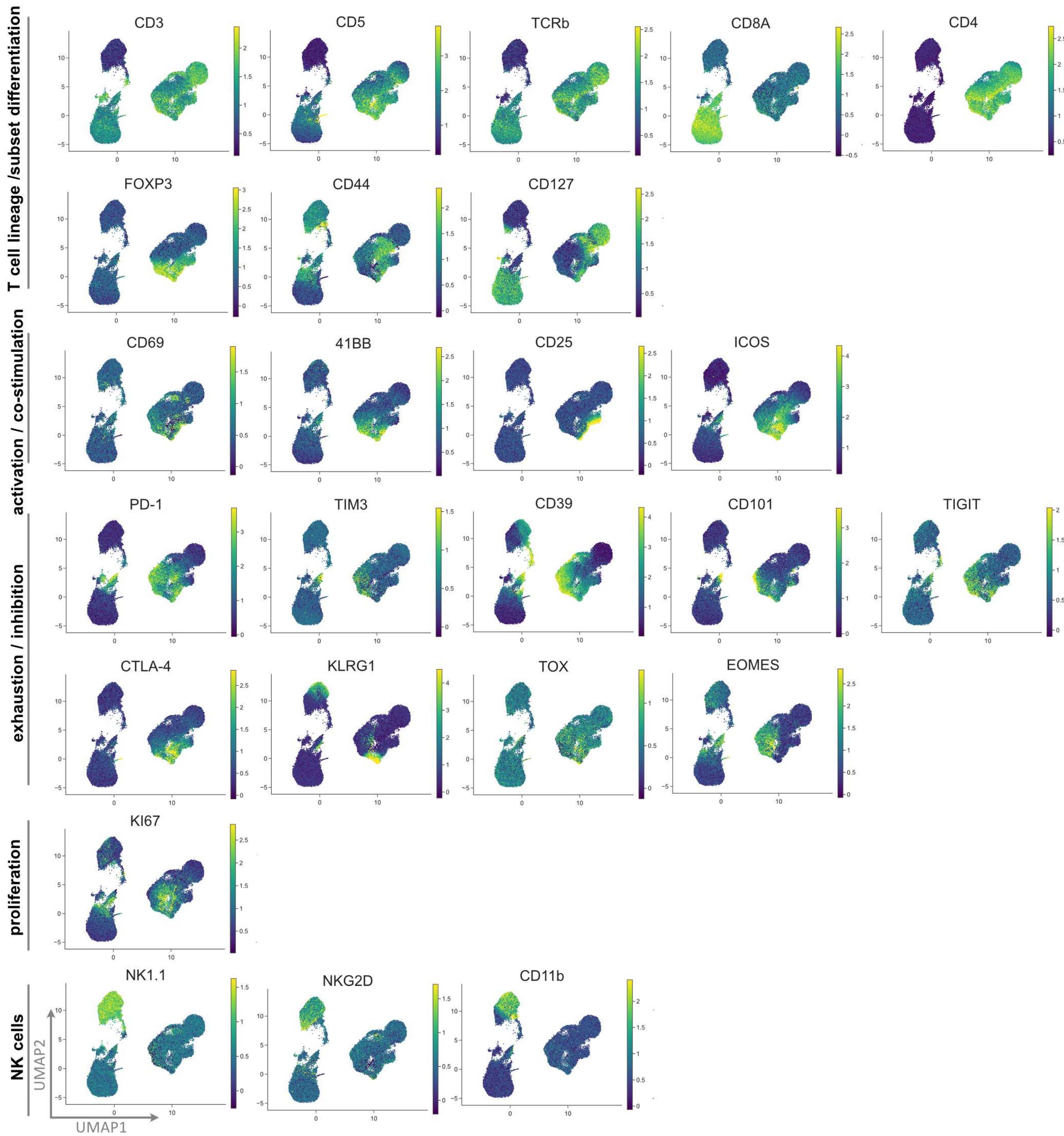

Supp. Fig. 9: UMAP projections of T/NK cell populations with overlaid marker expression

UMAP projection overlaid with T cell lineage and subset differentiation markers (CD3, CD5, TCR, CD8, CD4, FoxP3, CD44, CD127), activation/co-stimulatory markers (CD69, 41BB, CD25, ICOS), exhaustion/inhibitory markers (PD-1, TIM3, CD39, CD101, TIGIT, CTLA-4, KLRG1, TOX, Eomes), proliferation marker (Ki67) and NK cell markers (NK1.1, NKG2D, CD11b). Transformed fluorescent intensities (MFI) are represented by a color scale, with yellow indicating higher and blue indicating lower expression.

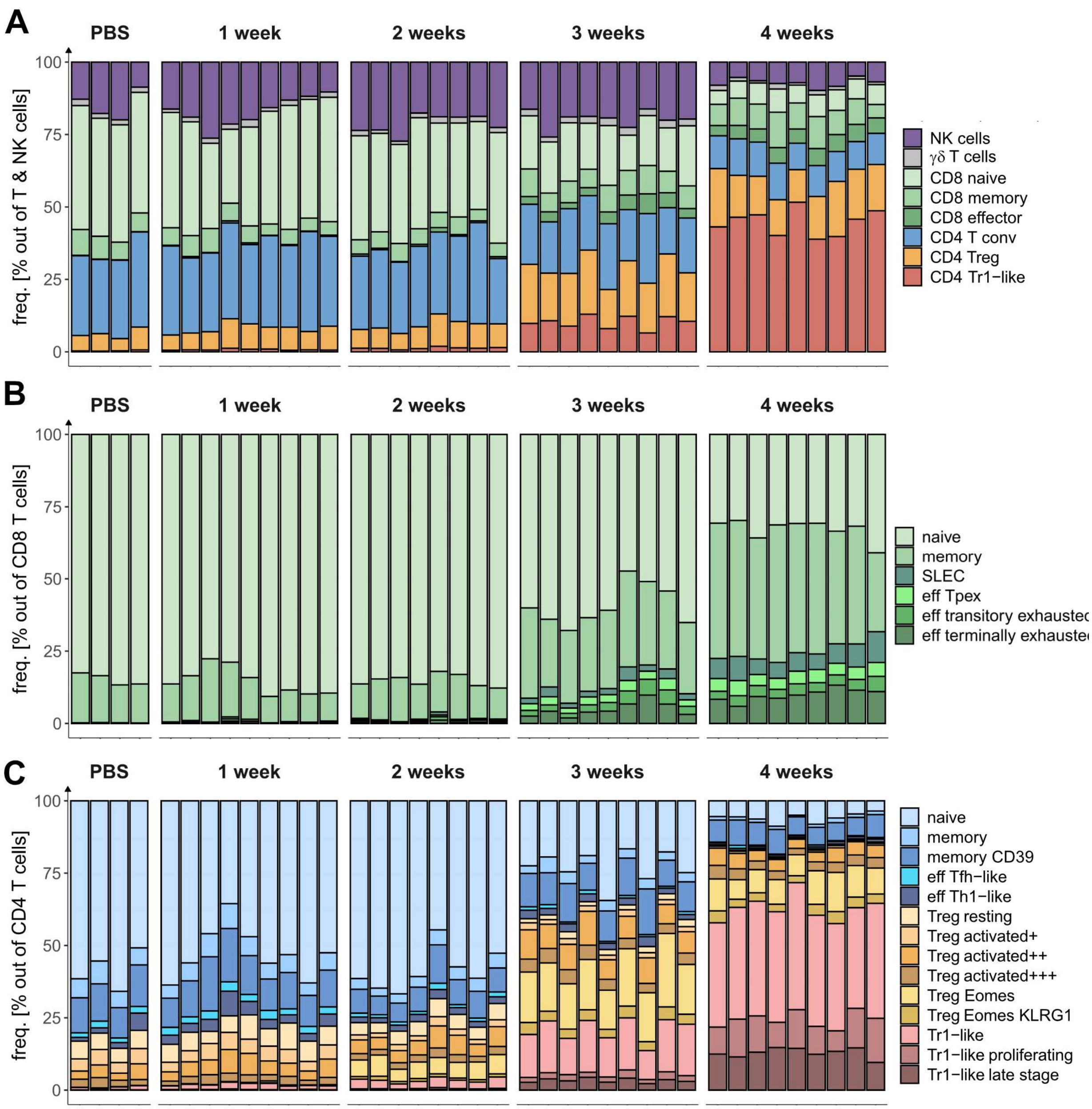

Supp. Fig. 10: Proportions of T and NK cell subsets per individual mouse

A-C) Proportions of T cell and NK cell subsets within all T and NK cells (A), $CD8^+$ T cells (B) and $CD4^+$ T cells (C) shown as stacked bar per individual mouse.

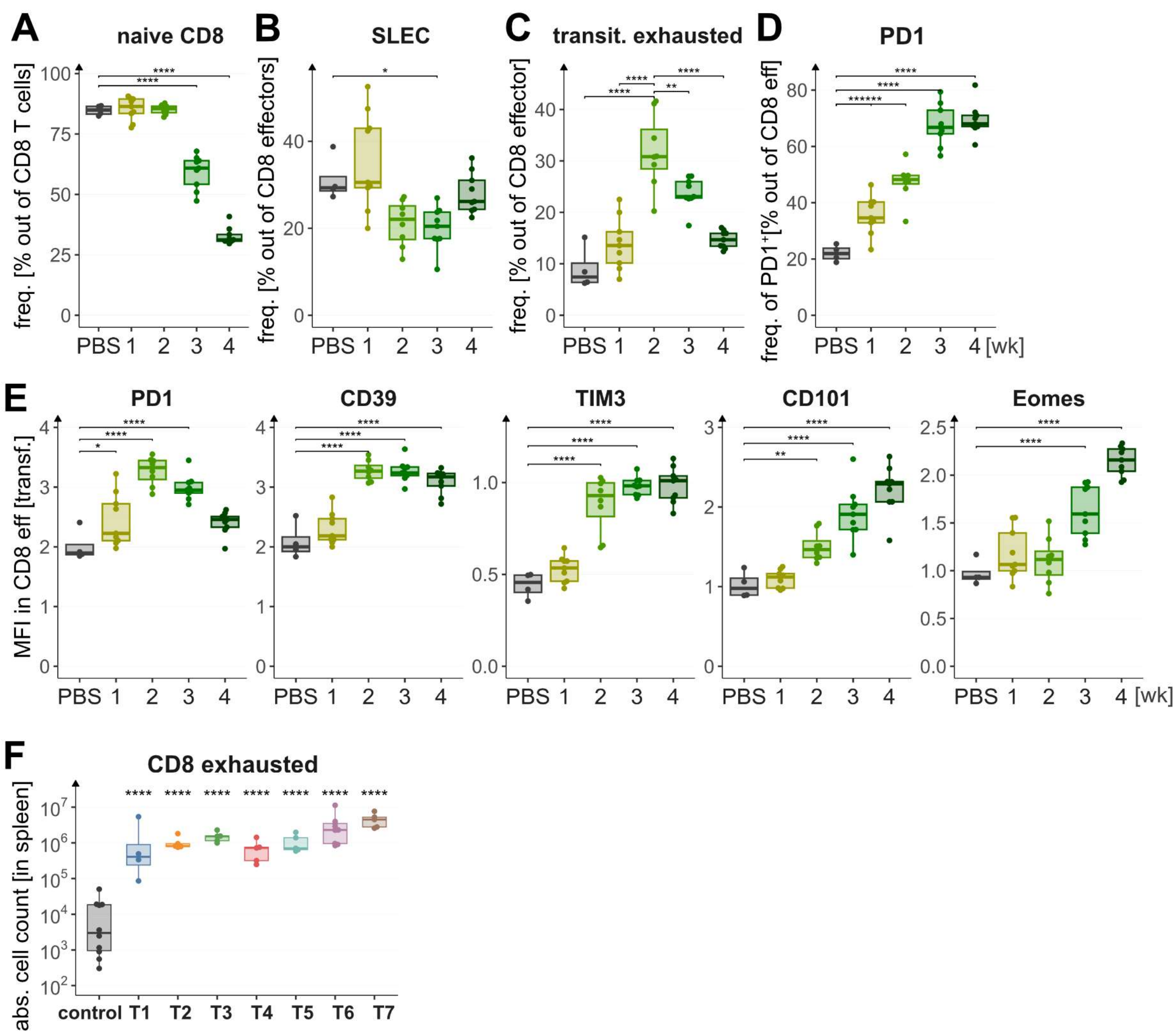

Supp. Fig. 11: Additional analysis of CD8⁺ T cell populations during CLL development

A-E) Quantitative analysis of CD8⁺ T cells in the spleen of mice before (PBS) and after 1-4 weeks (wk) after TCL1-AT using the spectral flow cytometry data shown in Figures 3 and 4 (n = 9 mice per time point, except for 2 weeks after AT, n = 8, PBS group n=4). A) Frequency of naïve CD8⁺ T cells within CD8⁺ T cells. B) Percentage of short-lived effector cells (SLEC) in CD8 effectors. C) Frequency of transitory exhausted CD8⁺ T cells within CD8⁺ effectors. Same data as in Fig. 3D; one-way ANOVA followed by Dunnett's multiple comparisons test was performed for all groups vs. 2 weeks after TCL1-AT. D) Percentage of PD-1⁺ population with CD8 effectors, defined by manual gating. E) Quantifications of PD-1, CD39, TIM3, CD101, and Eomes expression (transformed median fluorescent intensity (transf. MFI)) in CD8 effectors. F) Absolute cell count of exhausted CD8⁺ T cells, defined by the expression of PD1, CD39, and TIM3, in spleen from mice after seven independent TCL1 adoptive transfer (AT) experiments (T1-T7) and age-matched control mice. A-F) Box plots indicate medians with interquartile ranges (whiskers to 1.5×IQR). One-way ANOVA followed by Dunnett's multiple comparisons test (if not specified otherwise, each group vs. PBS control). ****$p < 0.0001$, ***$p \leq 0.001$, **$p \leq 0.01$, *$p \leq 0.05$.

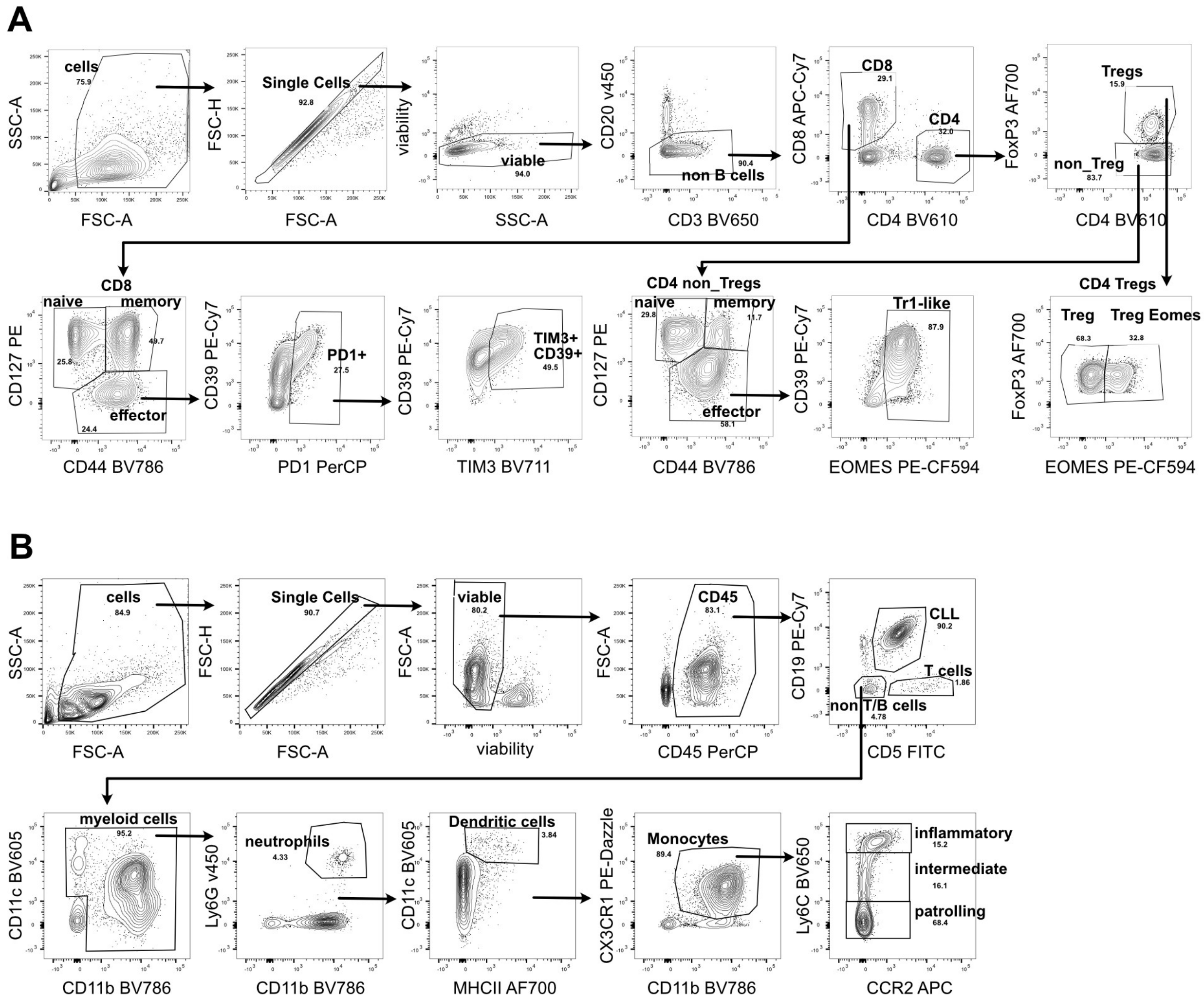

Supp. Fig. 12: Gating strategy for conventional flow cytometry-based analysis of T and myeloid cells in spleen

A) Representative gating strategy for conventional flow cytometry-based analysis of CD4⁺ Tregs, CD4⁺ Eomes⁺ Type 1 regulatory (Tr1)-like cells, naïve, memory, and effector CD8⁺ T cells, PD1⁺ TIM3⁺ CD39⁺ exhausted CD8⁺ T cell subset in spleen. B) Representative gating strategy for analysis of CLL, neutrophils, dendritic cells, inflammatory and patrolling monocytes.

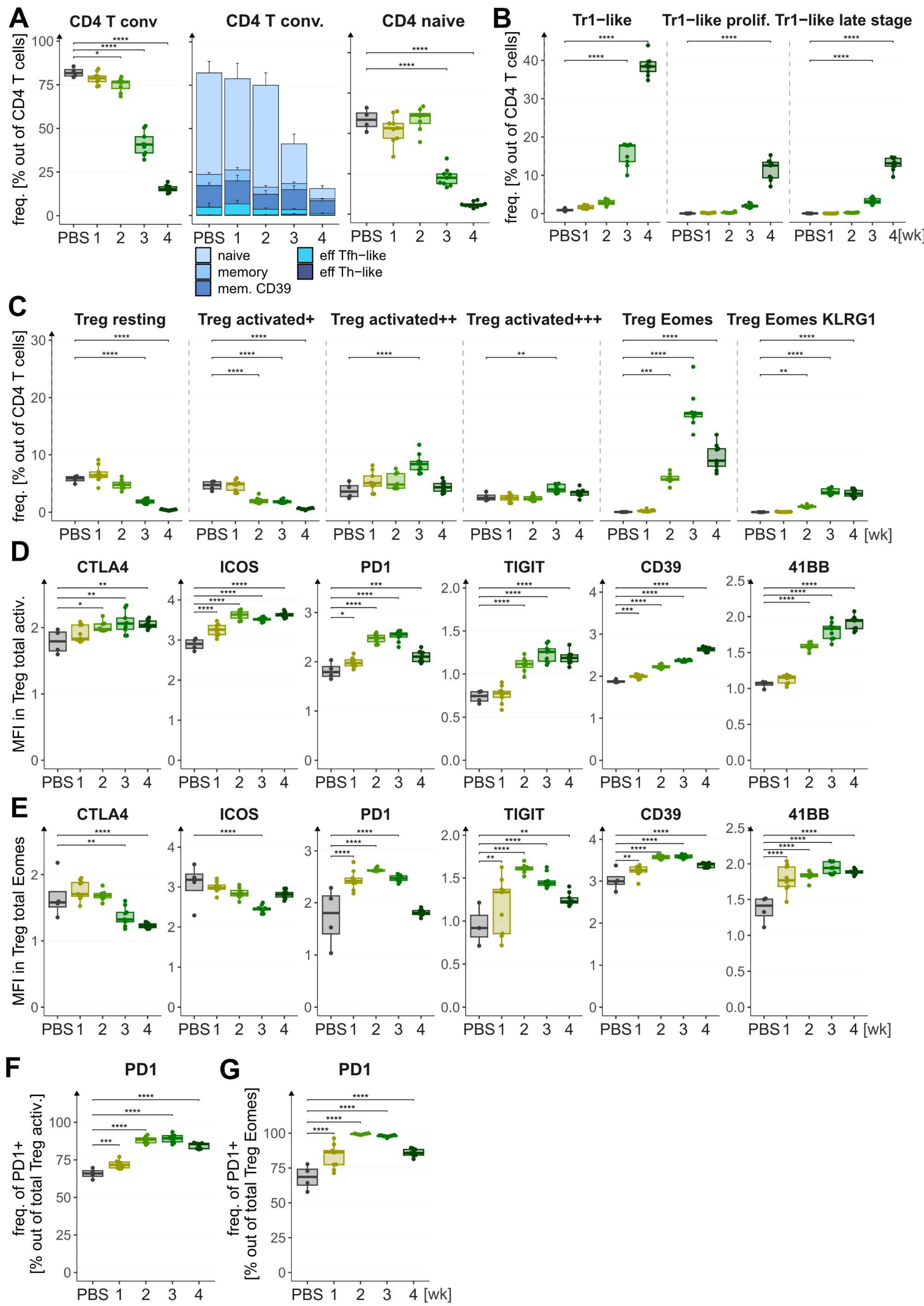
A
CD4 T conv
CD4 T conv.
CD4 naive
freq. [% out of CD4 T cells]
naive
memory
mem. CD39
eff Tfh−like
eff Th−like
B
Tr1−like
Tr1−like prolif.
Tr1−like late stage
freq. [% out of CD4 T cells]
PBS 1 2 3 4 [wk]
C
Treg resting
Treg activated+
Treg activated++
Treg activated+++
Treg Eomes
Treg Eomes KLRG1
freq. [% out of CD4 T cells]
D
CTLA4
ICOS
PD1
TIGIT
CD39
41BB
MFI in Treg total activ.
E
CTLA4
ICOS
PD1
TIGIT
CD39
41BB
MFI in Treg total Eomes
F
PD1
freq. of PD1+ [% out of total Treg activ.]
G
PD1
freq. of PD1+ [% out of total Treg Eomes]
PBS 1 2 3 4 [wk]

Supp. Fig. 13: Additional analysis of $CD4^+$ T cells during CLL development

Quantitative analysis of $CD4^+$ T cells in the spleen of mice before (PBS) and after 1-4 weeks (wk) after TCL1-AT using the spectral flow cytometry data shown in Figures 3 and 4 (n = 9 mice per time point, except for 2 weeks after AT, n = 8, PBS group n=4). A) Frequency of conventional (conv) $CD4^+$ T cells within $CD4^+$ T cells (left). Proportions of conventional $CD4^+$ T cell subsets (naïve, memory, memory CD39, T follicular helper (Tfh)-like, and T helper (Th)-like $CD4^+$ T cells) within $CD4^+$ T cells, shown as a stacked bar plot (middle). Percentage of naïve $CD4^+$ T cells out of $CD4^+$ T cells (right). B) Frequencies of three $Eomes^+$ regulatory type 1 (Tr1)-like $CD4^+$ T cell subsets within $CD4^+$ T cells. C) Percentages of six Treg subsets out of $CD4^+$ T cells. D-E) Quantifications of CTLA-4, ICOS, PD-1, TIGIT, CD39 and 41BB expression (transformed median fluorescent intensity (transf. MFI)) in total activated Tregs (D) and total $Eomes^+$ Tregs (E). F-G) Percentage of PD-1^+ population with total activated Tregs (F) and total $Eomes^+$ Tregs (G), defined by manual gating. A-G) Stacked bars show subset proportions within indicated parent populations as mean with SD error bars; box plots indicate medians with interquartile ranges (whiskers to 1.5×IQR). One-way ANOVA followed by Dunnett's multiple comparisons test (each group vs. PBS control). $****p < 0.0001$, $***p \leq 0.001$, $**p \leq 0.01$, $*p \leq 0.05$.

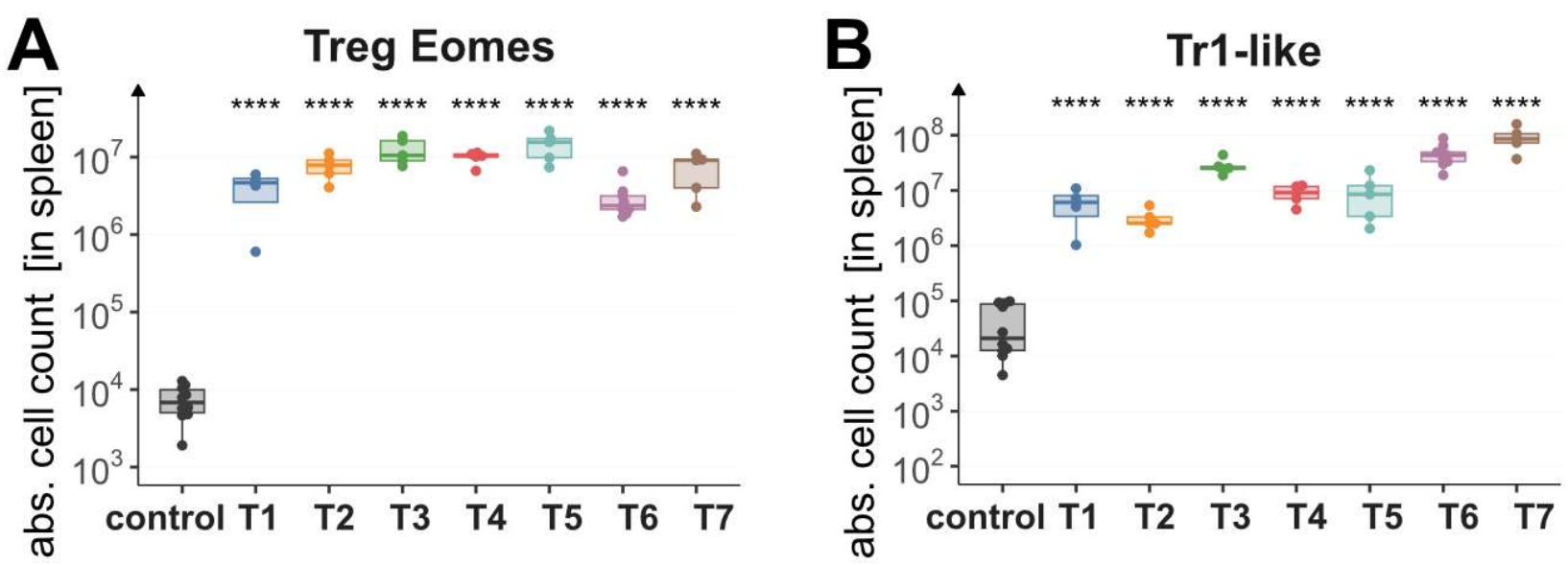

Supp. Fig. 14: Additional analysis of CD4⁺ T cells during CLL development

Quantitative analysis CD4⁺ T cells in spleen of mice after seven independent TCL1-AT experiments and age-matched control mice using the conventional data. A-B) Absolute cell counts of Treg Eomes (A) and Tr1-like cells (B) in spleen. Box plots indicate medians with interquartile ranges (whiskers to 1.5×IQR). One-way ANOVA followed by Dunnett's multiple comparisons test (each group vs. naïve control). ****p < 0.0001, ***p ≤ 0.001, **p ≤ 0.01, *p ≤ 0.05.

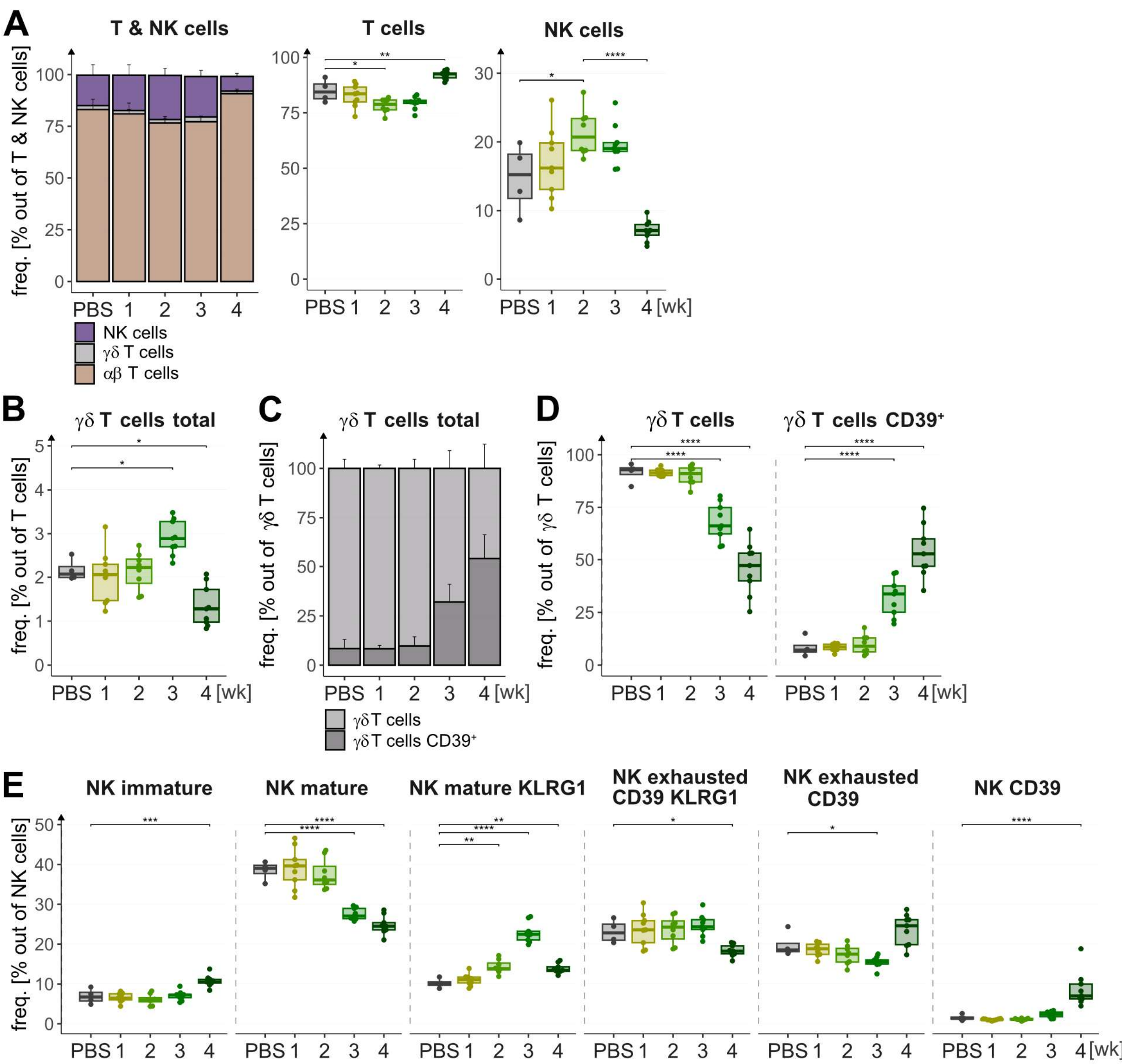

Supp. Fig. 15: Additional analysis of γδ T and NK cells during CLL development

Quantitative analysis of γδ T and NK cells in the spleen of mice before (PBS) and after 1-4 weeks (wk) after TCL1-AT using the spectral flow cytometry data described in Figures 3 and 4 (n = 9 mice per time point, except for 2 weeks after AT, n = 8, PBS group n=4). A) Proportions of NK, αβ T, and γδ T cells over time, shown as a stacked bar plot (left). Frequency of total T cells (middle) and NK cells (right) over time. For NK cells, same data as in Fig. 3K; one-way ANOVA followed by Dunnett's multiple comparisons test was performed for all groups vs. 2 weeks after TCL1-AT. B) Frequency of total γδ T cells within T cells. C-D) Proportions of γδ T cells and γδ T cells CD39⁺ within total γδ T cells, as stacked bar plot (B) and box plot (C). E) Frequencies of six NK cell subsets within total NK cells. A-E) Stacked bars show subset proportions within indicated parent populations as mean with SD error bars; box plots indicate medians with interquartile ranges (whiskers to 1.5×IQR). One-way ANOVA followed by Dunnett's multiple comparisons test (if not specified otherwise, each group vs. PBS control). ****$p < 0.0001$, ***$p \leq 0.001$, **$p \leq 0.01$, *$p \leq 0.05$.

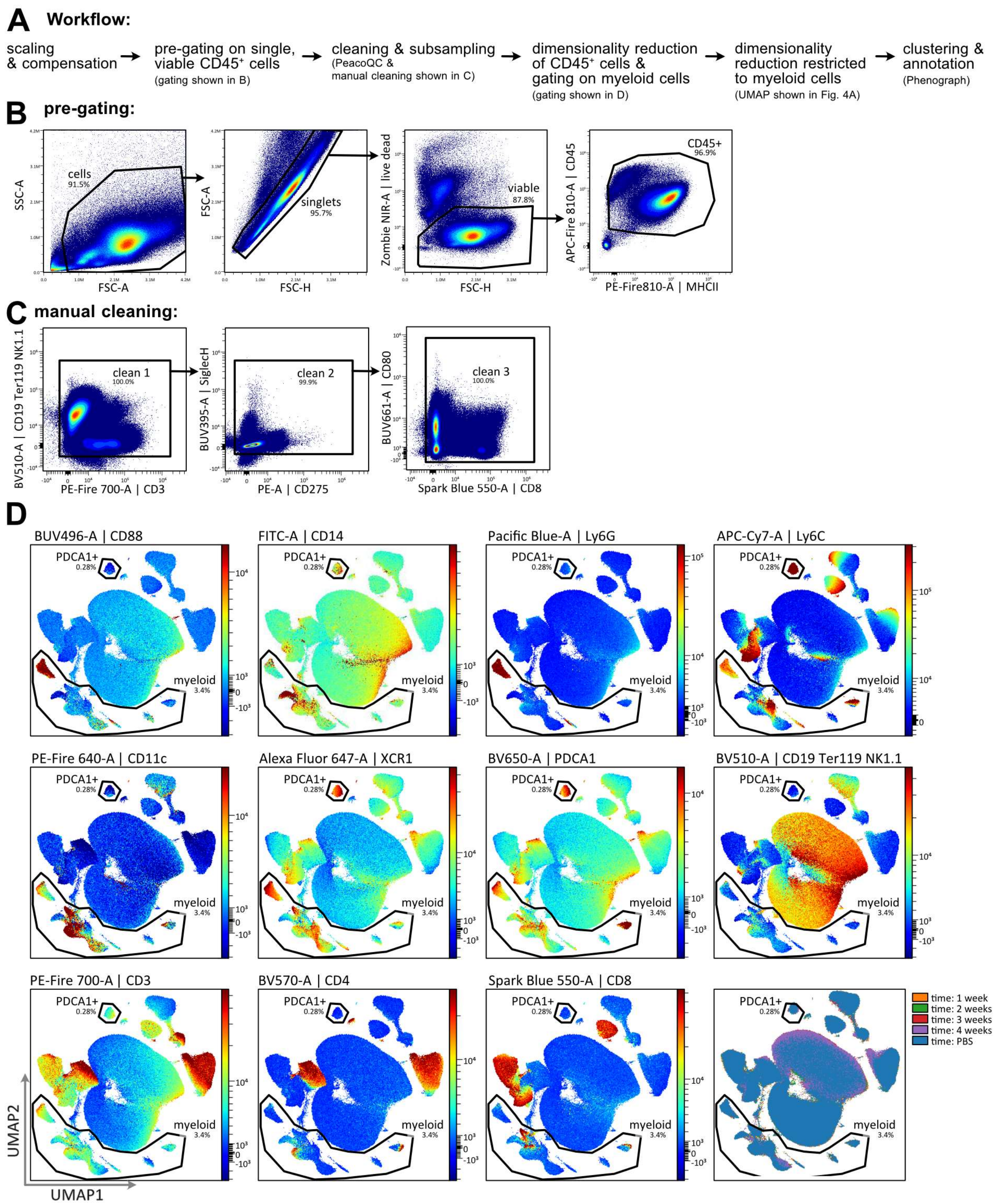

Supp. Fig. 16: Workflow and pre-gating for the spectral flow cytometry-based analysis of myeloid cells

A) Workflow of spectral flow cytometry-based analysis of monocytes, neutrophils and dendritic cells in the spleen. B) Pre-gating of single, viable CD45⁺ cells. C) Manual cleaning performed in addition to PeacoQC. D) Gating of myeloid clusters on UMAP of CD45⁺ cells based on expression of lymphoid and myeloid lineage markers. Shown gates for myeloid and PDCA1⁺ cells were used for dimensionality reduction restricted to myeloid cells.

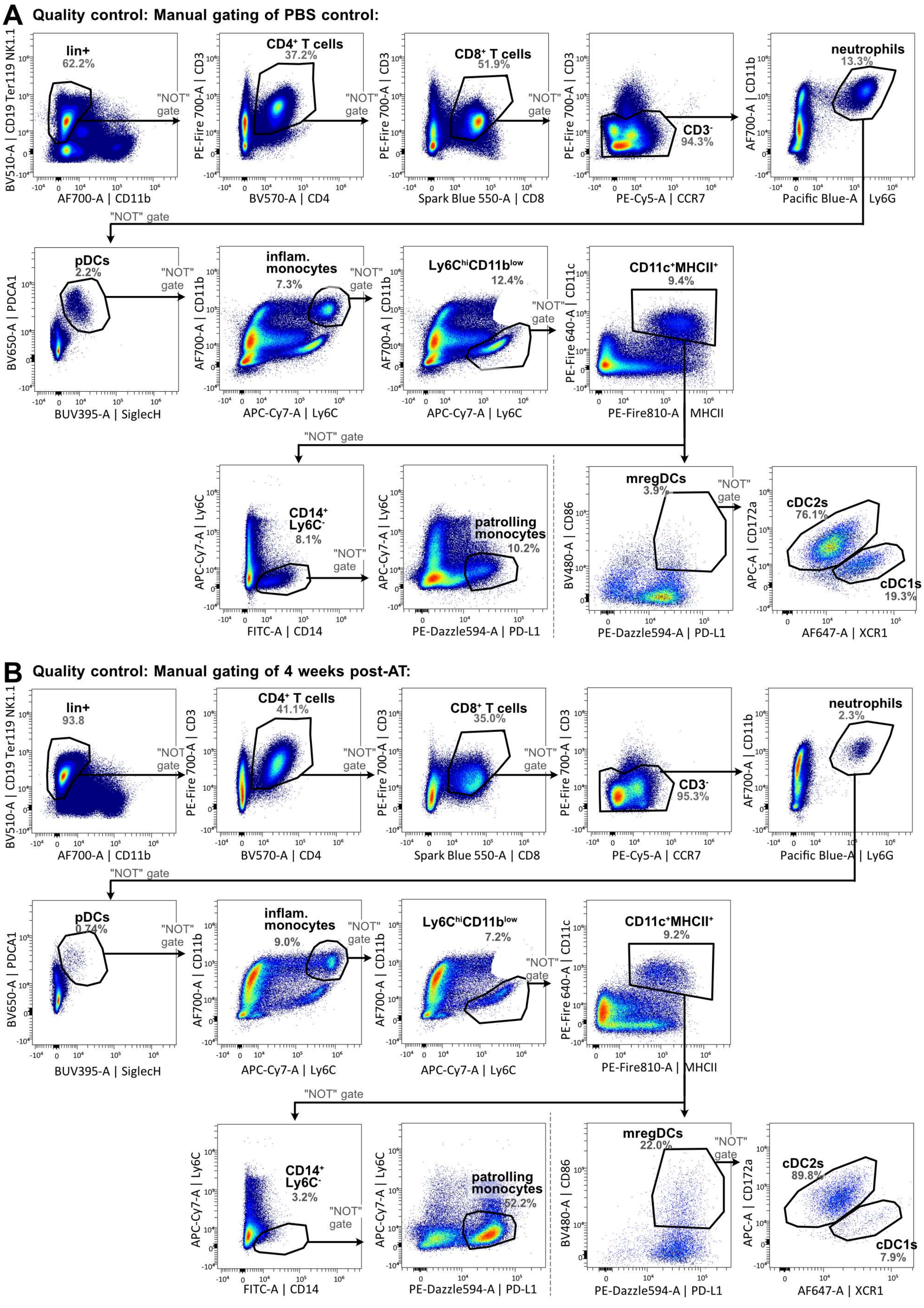
A Quality control: Manual gating of PBS control:
lin+
62.2%
CD4+ T cells
37.2%
CD8+ T cells
51.9%
CD3-
94.3%
neutrophils
13.3%
"NOT" gate
pDCs
2.2%
inflam. monocytes
7.3%
Ly6ChiCD11blow
12.4%
CD11c+MHCII+
9.4%
CD14+ Ly6C-
8.1%
patrolling monocytes
10.2%
mregDCs
3.9%
cDC2s
76.1%
cDC1s
19.3%
BV510-A | CD19 Ter119 NK1.1
AF700-A | CD11b
PE-Fire 700-A | CD3
BV570-A | CD4
Spark Blue 550-A | CD8
PE-Cy5-A | CCR7
Pacific Blue-A | Ly6G
BV650-A | PDCA1
BUV395-A | SiglecH
APC-Cy7-A | Ly6C
PE-Fire 640-A | CD11c
PE-Fire810-A | MHCII
FITC-A | CD14
PE-Dazzle594-A | PD-L1
BV480-A | CD86
APC-A | CD172a
AF647-A | XCR1
B Quality control: Manual gating of 4 weeks post-AT:
lin+
93.8
CD4+ T cells
41.1%
CD8+ T cells
35.0%
CD3-
95.3%
neutrophils
2.3%
pDCs
0.74%
inflam. monocytes
9.0%
Ly6ChiCD11blow
7.2%
CD11c+MHCII+
9.2%
CD14+ Ly6C-
3.2%
patrolling monocytes
52.2%
mregDCs
22.0%
cDC2s
89.8%
cDC1s
7.9%

Supp. Fig. 17: Manual gating strategy of myeloid cell spectral flow cytometry panel

A-B) Manual gating of myeloid cells from PBS control mice (A) or mice 4 weeks post-AT (B). First, lineage^{+} (CD19, Ter119, NK1.1) cells were excluded, as well as CD4^{+} or CD8^{+} or CD3^{+} T cells. Neutrophils were identified based on Ly6G and CD11b expression, in consistence with [24,25], followed by plasmacytoid dendritic cell (pDCs) gating (Siglec-H^{+} PDCA-1^{+}), based on [26-29]. Subsequently, Ly6C^{high} CD11b^{+} inflammatory monocytes as well as Ly6C^{high} CD11b^{low} cells were defined, in line with [30,31]. Based on the expression of MHC II and CD11c, conventional DCs (cDCs) were gated. Within cDCs, mature immune regulatory DCs (mregDCs) were identified based on PD-L1 and CD86 expression; CCR7 and ICAM-1 expression was also confirmed (not shown), based on [32-34]. cDCs not exhibiting a mregDC phenotype were subgated into cDC2s (CD172a^{+} XCR1^{-}) and cDC1s (CD172a^{-} XCR1^{+}), in accordance with [26-29,35,36]. After excluding DCs, a CD14^{+} Ly6C^{-} myeloid subset as well as PD-L1^{high} Ly6C$^{-/low}$ patrolling monocytes were defined, in line with [30,31]. A-B) All mice per group concatenated. The marker expression of annotated myeloid cell populations and corresponding literature referencesare also listed in Suppl. Table 3.

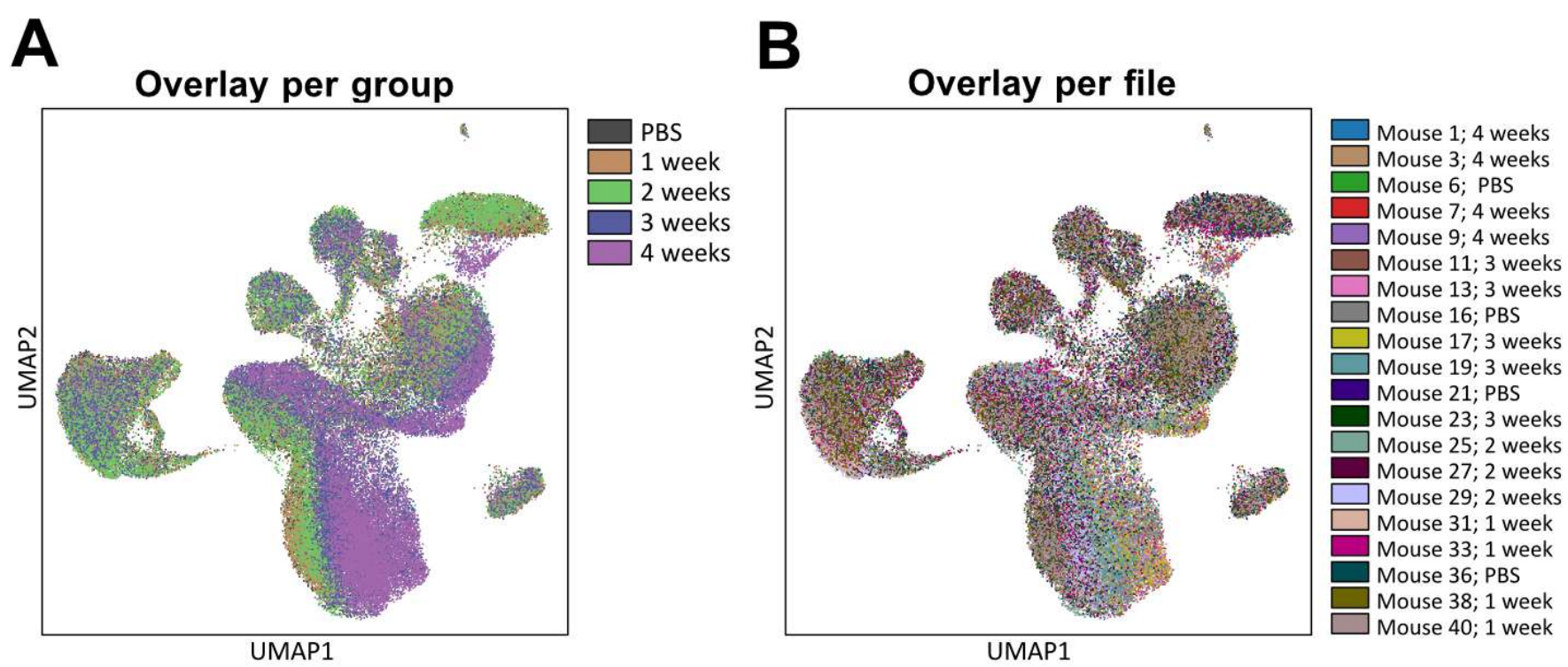

Supp. Fig. 18: UMAP projections of myeloid cell populations with overlaid groups and individual mice

UMAP projections of myeloid cells with overlaid groups (A, PBS control and 1-4 weeks post-AT) and all individual mice (B, n=20).

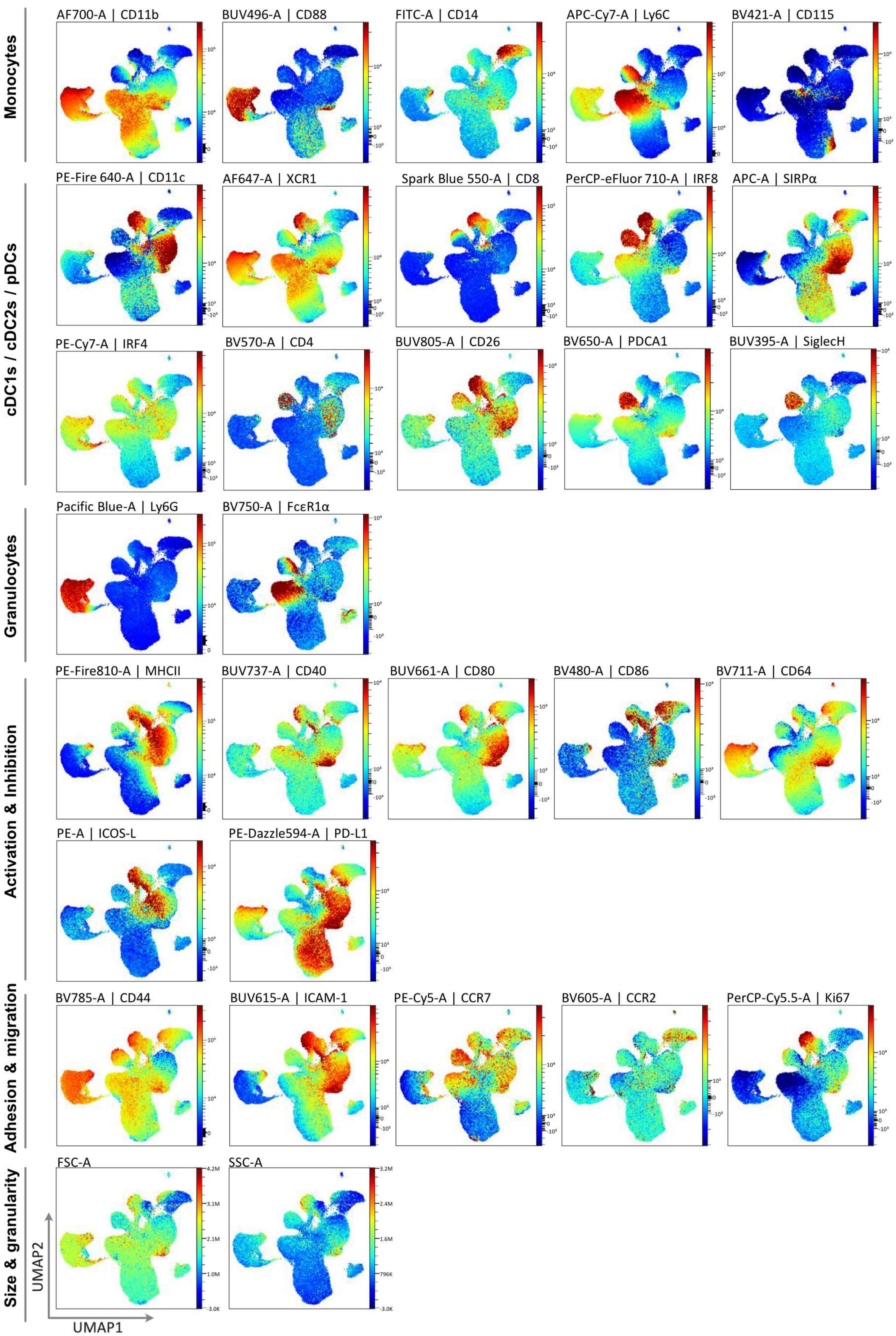
Monocytes
AF700-A | CD11b
BUV496-A | CD88
FITC-A | CD14
APC-Cy7-A | Ly6C
BV421-A | CD115
cDC1s / cDC2s / pDCs
PE-Fire 640-A | CD11c
AF647-A | XCR1
Spark Blue 550-A | CD8
PerCP-eFluor 710-A | IRF8
APC-A | SIRPα
PE-Cy7-A | IRF4
BV570-A | CD4
BUV805-A | CD26
BV650-A | PDCA1
BUV395-A | SiglecH
Granulocytes
Pacific Blue-A | Ly6G
BV750-A | FcεR1α
Activation & Inhibition
PE-Fire810-A | MHCII
BUV737-A | CD40
BUV661-A | CD80
BV480-A | CD86
BV711-A | CD64
PE-A | ICOS-L
PE-Dazzle594-A | PD-L1
Adhesion & migration
BV785-A | CD44
BUV615-A | ICAM-1
PE-Cy5-A | CCR7
BV605-A | CCR2
PerCP-Cy5.5-A | Ki67
Size & granularity
FSC-A
SSC-A
UMAP2
UMAP1

Supp. Fig. 19: UMAP projections of myeloid cell populations with overlaid marker expression

UMAP projection overlaid with the following markers: general (CD11b), monocytes (CD88, CD14, Ly6C, CD115), dendritic cells (CD11c; cDC1s: XCR1, CD8, IRF8; cDC2s: SIRPα/CD172α, IRF4; pDCs: PDCA-1 and Siglec-H), granulocytes (Ly6G, FcεR1), activation/co-stimulation marker (MHCII, CD40, CD80, CD86, CD64, ICOS-L/CD275), inhibition marker (PD-L1/CD274), migration/adhesion marker (CD44, ICAM-1/CD54, CCR7/CD197, CCR2/CD192), proliferation marker (Ki67) and size/granularity parameters (FSC, SSC). Median fluorescent intensities (MFIs) are represented by a color scale, with red indicating higher and blue indicating lower expression.

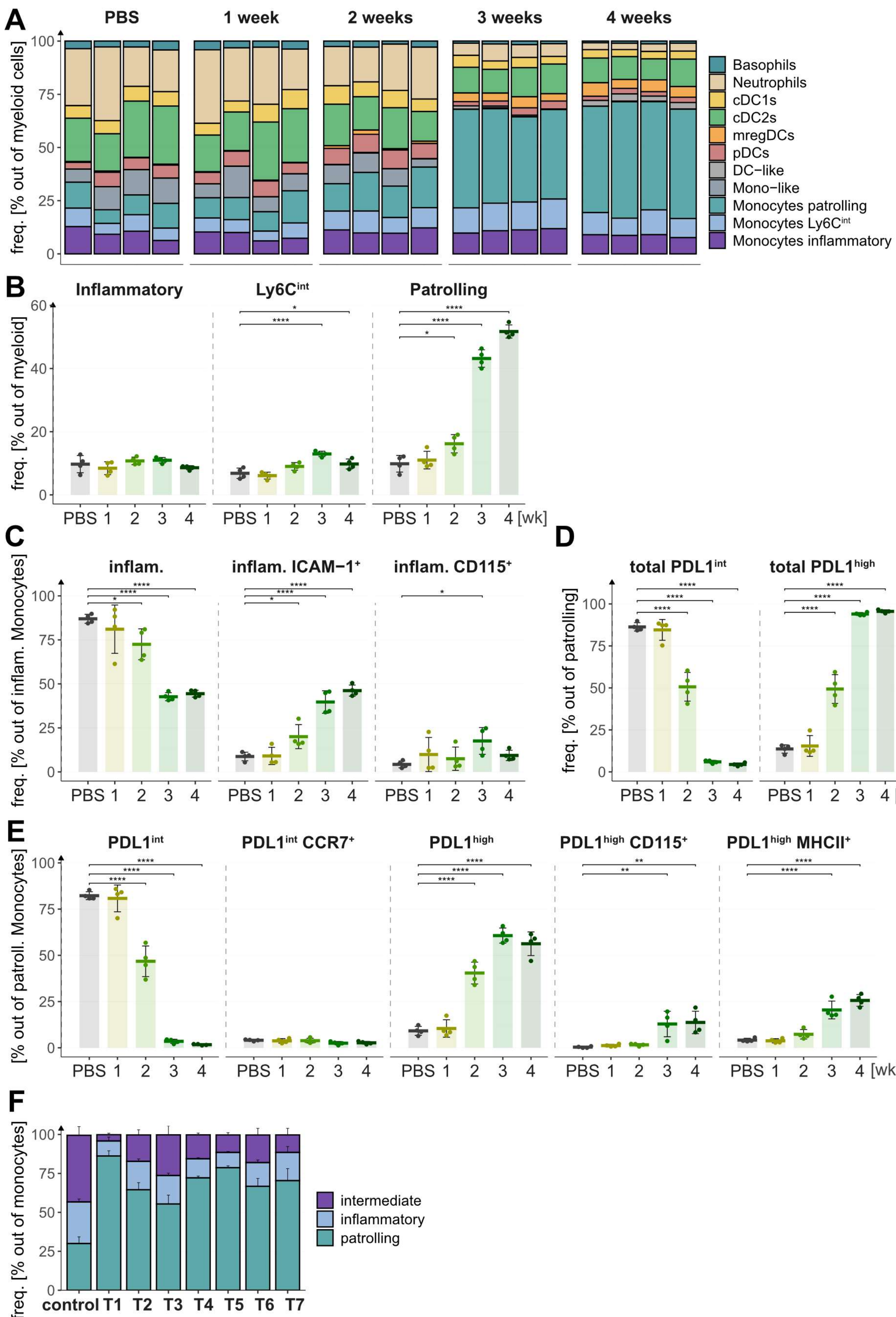

A
PBS
1 week
2 weeks
3 weeks
4 weeks
freq. [% out of myeloid cells]
Basophils
Neutrophils
cDC1s
cDC2s
mregDCs
pDCs
DC−like
Mono−like
Monocytes patrolling
Monocytes Ly6C^int
Monocytes inflammatory
B
Inflammatory
Ly6C^int
Patrolling
freq. [% out of myeloid]
PBS 1 2 3 4 [wk]
C
inflam.
inflam. ICAM−1+
inflam. CD115+
freq. [% out of inflam. Monocytes]
D
total PDL1^int
total PDL1^high
freq. [% out of patrolling]
E
PDL1^int
PDL1^int CCR7+
PDL1^high
PDL1^high CD115+
PDL1^high MHCII+
[% out of patroll. Monocytes]
F
freq. [% out of monocytes]
control T1 T2 T3 T4 T5 T6 T7
intermediate
inflammatory
patrolling

Supp. Fig. 20: Additional analysis of monocyte subsets during CLL development

A-E) Quantitative analysis of monocytes in the spleen of mice before (PBS) and after 1-4 weeks (wk) after TCL1-AT (n = 4 mice per group) using the spectral flow cytometry data depicted in Figures 5, 6, and 7. A) Proportions of basophils, neutrophils, DC subsets and monocyte subsets within all myeloid cells shown as stacked bar per individual mouse. B) Frequencies of inflammatory, $Ly6C^{int}$ and patrolling monocytes within myeloid cells. C) Proportions of three inflammatory monocyte subsets within inflammatory monocytes. D) Percentages of total $PD\text{-}L1^{int}$ and total $PD\text{-}L1^{high}$ patrolling monocytes within total patrolling monocytes. E) Frequencies of five patrolling monocyte subsets within patrolling monocytes over time. F) Proportion of inflammatory, intermediate and patrolling monocytes in the spleens of mice after seven independent TCL1-AT experiments and age-matched control mice, shown as stacked bar plot. A-F) Stacked bars show subset proportions within indicated parent populations as mean with SD error bars; bar plots indicate mean with SD. One-way ANOVA followed by Dunnett's multiple comparisons test (each group vs. PBS control). ****p < 0.0001, ***p ≤ 0.001, **p ≤ 0.01, *p ≤ 0.05.

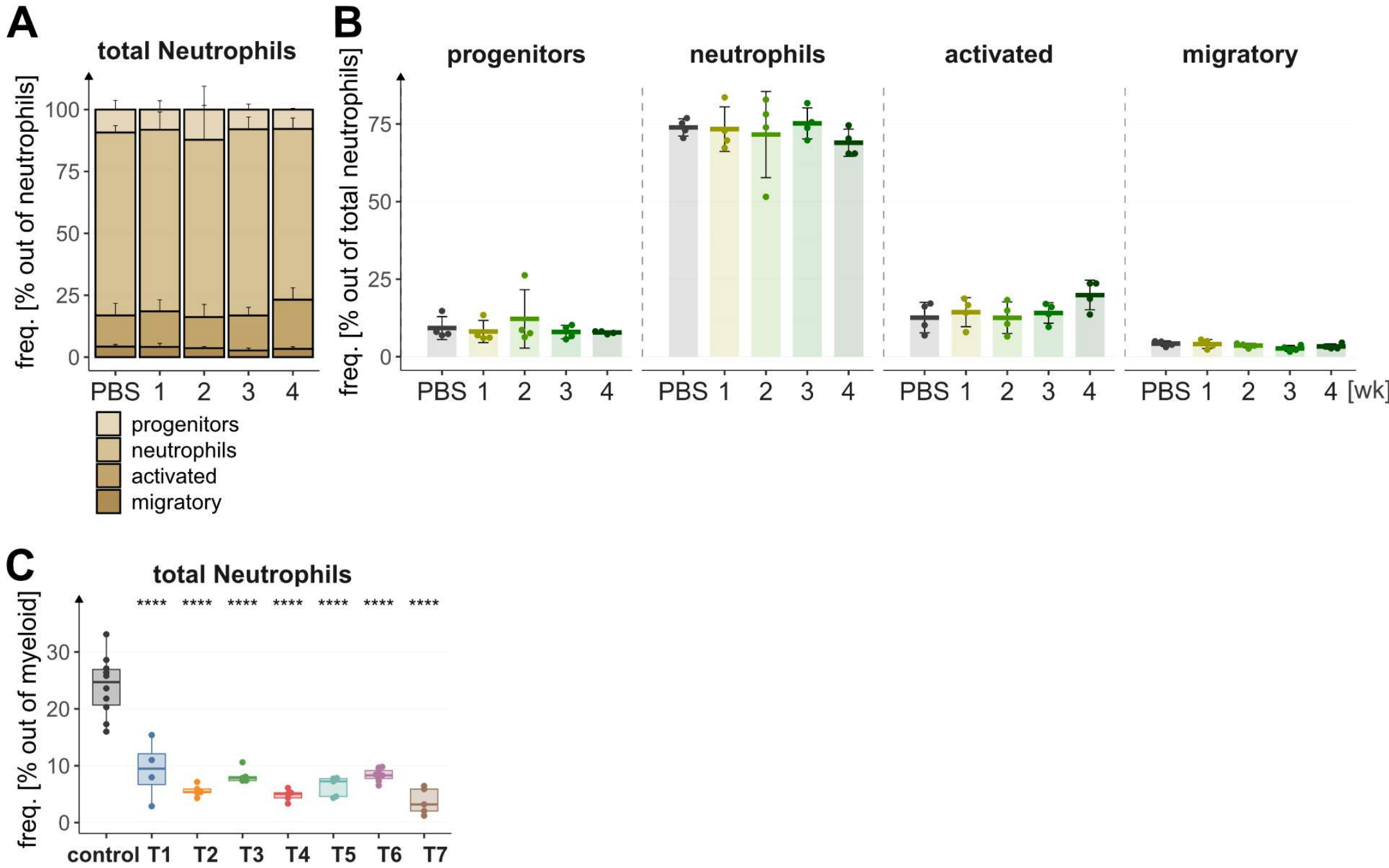

Supp. Fig. 21: Additional analysis of neutrophils subsets during CLL development

A-B) Quantitative analysis of neutrophils in the spleen of mice before (PBS) and after 1-4 weeks (wk) after TCL1-AT (n = 4 mice per group) using the spectral flow cytometry data depicted in Figures 5, 6, and 7. Proportions of neutrophils subsets within total neutrophils, as stacked bar plot (A) and bar plot (B). C) Frequency of neutrophils out of myeloid cells in spleen from mice after seven independent TCL1 adoptive transfer (AT) experiments (T1-T7) and age-matched control mice. A-B) Stacked bars show subset proportions within indicated parent populations as mean with SD error bars; bar plots indicate mean with SD. C) Box plots indicate medians with interquartile ranges (whiskers to 1.5×IQR). One-way ANOVA followed by Dunnett's multiple comparisons test (each group vs. naïve control).

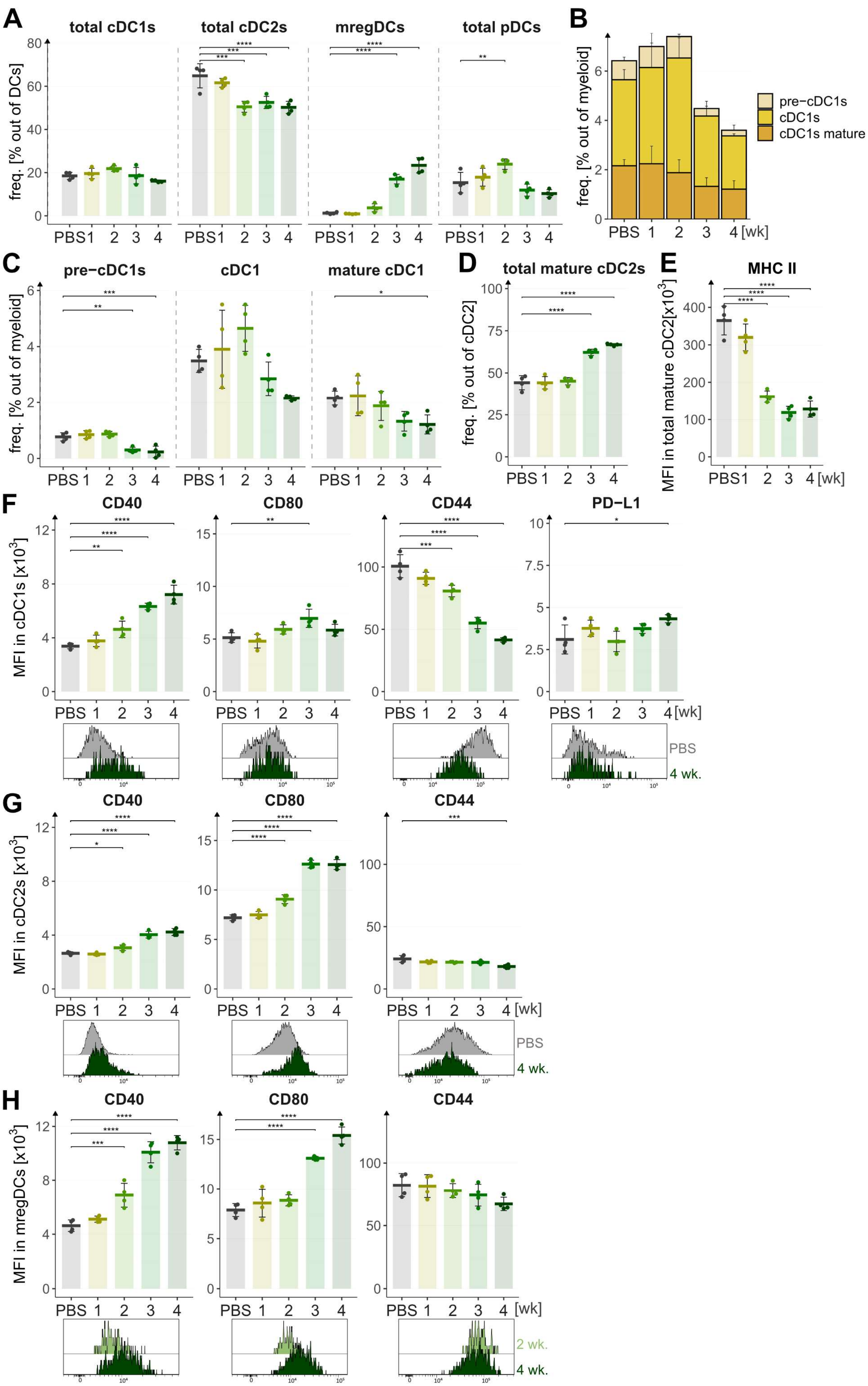
A
total cDC1s
total cDC2s
mregDCs
total pDCs
freq. [% out of DCs]
PBS 1 2 3 4
B
freq. [% out of myeloid]
pre−cDC1s
cDC1s
cDC1s mature
PBS 1 2 3 4 [wk]
C
pre−cDC1s
cDC1
mature cDC1
freq. [% out of myeloid]
D
total mature cDC2s
freq. [% out of cDC2]
E
MHC II
MFI in total mature cDC2[x10^3]
F
CD40
CD80
CD44
PD−L1
MFI in cDC1s [x10^3]
PBS
4 wk.
G
CD40
CD80
CD44
MFI in cDC2s [x10^3]
PBS
4 wk.
H
CD40
CD80
CD44
MFI in mregDCs [x10^3]
2 wk.
4 wk.

Supp. Fig. 22: Frequency of additional dendritic cell subsets during CLL development

Quantitative analysis of dendritic cells (DCs) in the spleen of mice before (PBS) and after 1-4 weeks (wk) after TCL1-AT (n = 4 mice per group) using the spectral flow cytometry data shown in Figures 5, 6, and 7. A) Frequencies of total cDC1s, cDC2s, mregDCs, and pDCs within total DC. B-C) Proportions of cDC1 subsets within myeloid cells, shown as stacked bar plot (B) and box plot (C). D) Frequency of total mature cDC2s within cDC2s. E-H) Quantification of MHCII, CD40, CD80, CD44, and/or PD-L1 expression (median fluorescent intensity (MFI)) in total mature cDC2s (E), cDC1s (F)), cDC2s (G), and mregDCs (H) (above). Histograms of marker expression in respective DC subsets from PBS control mice (F, G, grey), 2 weeks (H, light green) or 4 weeks (F-H, dark green) after AT (below, all mice per group concatenated). A-H) Stacked bars show subset proportions within indicated parent populations as mean with SD error bars; bar plots indicate mean with SD. One-way ANOVA followed by Dunnett's multiple comparisons test (each group vs. PBS control). ****$p < 0.0001$, ***$p \leq 0.001$, **$p \leq 0.01$, *$p \leq 0.05$.

References for Supplementary Figures

1. Masopust D, Awasthi A, Bosselut R, et al. Guidelines for T cell nomenclature. *Nat Rev Immunol*. 2026;26(4):298-313.

2. Otsuka A, Hanakawa S, Miyachi Y, Kabashima K. CD39: a new surface marker of mouse regulatory gammadelta T cells. *J Allergy Clin Immunol*. 2013;132(6):1448-1451.

3. Hu G, Wu P, Cheng P, et al. Tumor-infiltrating CD39(+)gammadeltaTregs are novel immunosuppressive T cells in human colorectal cancer. *Oncoimmunology*. 2017;6(2):e1277305.

4. Daley D, Zambirinis CP, Seifert L, et al. gammadelta T Cells Support Pancreatic Oncogenesis by Restraining alphabeta T Cell Activation. *Cell*. 2016;166(6):1485-1499 e1415.

5. Ager CR, Zhang M, Chaimowitz M, et al. KLRG1 marks tumor-infiltrating CD4 T cell subsets associated with tumor progression and immunotherapy response. *J Immunother Cancer*. 2023;11(9).

6. Kornete M, Sgouroudis E, Piccirillo CA. ICOS-dependent homeostasis and function of Foxp3+ regulatory T cells in islets of nonobese diabetic mice. *J Immunol*. 2012;188(3):1064-1074.

7. Borsellino G, Kleinewietfeld M, Di Mitri D, et al. Expression of ectonucleotidase CD39 by Foxp3+ Treg cells: hydrolysis of extracellular ATP and immune suppression. *Blood*. 2007;110(4):1225-1232.

8. Cheng G, Yuan X, Tsai MS, Podack ER, Yu A, Malek TR. IL-2 receptor signaling is essential for the development of Klrg1+ terminally differentiated T regulatory cells. *J Immunol*. 2012;189(4):1780-1791.

9. Zhang P, Lee JS, Gartlan KH, et al. Eomesodermin promotes the development of type 1 regulatory T (T(R)1) cells. *Sci Immunol*. 2017;2(10).

10. Roessner PM, Llao Cid L, Lupar E, et al. EOMES and IL-10 regulate antitumor activity of T regulatory type 1 CD4(+) T cells in chronic lymphocytic leukemia. *Leukemia*. 2021;35(8):2311-2324.

11. Hanna BS, Llao-Cid L, Iskar M, et al. Interleukin-10 receptor signaling promotes the maintenance of a PD-1(int) TCF-1(+) CD8(+) T cell population that sustains anti-tumor immunity. *Immunity*. 2021;54(12):2825-2841 e2810.

12. Llao-Cid L, Roessner PM, Chapaprieta V, et al. EOMES is essential for antitumor activity of CD8(+) T cells in chronic lymphocytic leukemia. *Leukemia*. 2021;35(11):3152-3162.

13. Johnston RJ, Poholek AC, DiToro D, et al. Bcl6 and Blimp-1 are reciprocal and antagonistic regulators of T follicular helper cell differentiation. *Science*. 2009;325(5943):1006-1010.

14. Joshi NS, Cui W, Chandele A, et al. Inflammation directs memory precursor and short-lived effector CD8(+) T cell fates via the graded expression of T-bet transcription factor. *Immunity*. 2007;27(2):281-295.

15. Franco F, Jaccard A, Romero P, Yu YR, Ho PC. Metabolic and epigenetic regulation of T-cell exhaustion. *Nat Metab*. 2020;2(10):1001-1012.

16. Gupta PK, Godec J, Wolski D, et al. CD39 Expression Identifies Terminally Exhausted CD8+ T Cells. *PLoS Pathog*. 2015;11(10):e1005177.

17. Hudson WH, Gensheimer J, Hashimoto M, et al. Proliferating Transitory T Cells with an Effector-like Transcriptional Signature Emerge from PD-1(+) Stem-like CD8(+) T Cells during Chronic Infection. *Immunity*. 2019;51(6):1043-1058 e1044.

18. Huntington ND, Tabarias H, Fairfax K, et al. NK cell maturation and peripheral homeostasis is associated with KLRG1 up-regulation. *J Immunol*. 2007;178(8):4764-4770.

19. Zhang Y, Chen S, Tang X, et al. The role of KLRG1: a novel biomarker and new therapeutic target. *Cell Commun Signal*. 2024;22(1):337.

20. Kim S, Iizuka K, Kang HS, et al. In vivo developmental stages in murine natural killer cell maturation. *Nat Immunol*. 2002;3(6):523-528.

21. Gordon SM, Chaix J, Rupp LJ, et al. The transcription factors T-bet and Eomes control key checkpoints of natural killer cell maturation. *Immunity*. 2012;36(1):55-67.

22. Robbins SH, Nguyen KB, Takahashi N, Mikayama T, Biron CA, Brossay L. Cutting edge: inhibitory functions of the killer cell lectin-like receptor G1 molecule during the activation of mouse NK cells. *J Immunol*. 2002;168(6):2585-2589.

23. Young A, Ngiow SF, Gao Y, et al. A2AR Adenosine Signaling Suppresses Natural Killer Cell Maturation in the Tumor Microenvironment. *Cancer Res*. 2018;78(4):1003-1016.

24. Ng LG, Ostuni R, Hidalgo A. Heterogeneity of neutrophils. *Nat Rev Immunol*. 2019;19(4):255-265.

25. Lagasse E, Weissman IL. Flow cytometric identification of murine neutrophils and monocytes. *J Immunol Methods*. 1996;197(1-2):139-150.

26. Guilliams M, Ginhoux F, Jakubzick C, et al. Dendritic cells, monocytes and macrophages: a unified nomenclature based on ontogeny. *Nat Rev Immunol*. 2014;14(8):571-578.

27. Guilliams M, Dutertre CA, Scott CL, et al. Unsupervised High-Dimensional Analysis Aligns Dendritic Cells across Tissues and Species. *Immunity*. 2016;45(3):669-684.

28. Amon L, Seichter A, Vurnek D, et al. Protocol for mapping the heterogeneous dendritic cell network across the murine tissue landscape via high-dimensional flow cytometry. *STAR Protoc*. 2024;5(3):103151.

29. Backer RA, Probst HC, Clausen BE. Multiparameter Flow Cytometric Analysis of the Conventional and Monocyte-Derived DC Compartment in the Murine Spleen. *Vaccines (Basel)*. 2024;12(11).

30. Auffray C, Fogg D, Garfa M, et al. Monitoring of blood vessels and tissues by a population of monocytes with patrolling behavior. *Science*. 2007;317(5838):666-670.

31. Hanna BS, McClanahan F, Yazdanparast H, et al. Depletion of CLL-associated patrolling monocytes and macrophages controls disease development and repairs immune dysfunction in vivo. *Leukemia*. 2016;30(3):570-579.

32. Maier B, Leader AM, Chen ST, et al. A conserved dendritic-cell regulatory program limits antitumour immunity. *Nature*. 2020;580(7802):257-262.

33. Li J, Zhou J, Huang H, Jiang J, Zhang T, Ni C. Mature dendritic cells enriched in immunoregulatory molecules (mregDCs): A novel population in the tumour microenvironment and immunotherapy target. *Clin Transl Med*. 2023;13(2):e1199.

34. Lee CYC, Kennedy BC, Richoz N, et al. Tumour-retained activated CCR7(+) dendritic cells are heterogeneous and regulate local anti-tumour cytolytic activity. *Nat Commun*. 2024;15(1):682.

35. Dorner BG, Dorner MB, Zhou X, et al. Selective expression of the chemokine receptor XCR1 on cross-presenting dendritic cells determines cooperation with CD8+ T cells. *Immunity*. 2009;31(5):823-833.

36. Crozat K, Tamoutounour S, Vu Manh TP, et al. Cutting edge: expression of XCR1 defines mouse lymphoid-tissue resident and migratory dendritic cells of the CD8alpha+ type. *J Immunol*. 2011;187(9):4411-4415.
