# Supplementary material for: High‐dimensional spectral flow cytometry uncovers progressive T‐cell exhaustion and myeloid cell reprogramming in a CLL mouse model
Source: Hemasphere. 2026 Sep 14;10(9):e70476. doi: 10.1002/hem3.70476 (PMC13573940; doi:10.1002/hem3.70476)
Supplement: Supplementary file 2 [file HEM3-10-e70476-s003.pdf]

Supporting Information S2: Supplementary Methods

Conventional Flow Cytometry

Single-cell suspensions (3×10^6) from spleen, BM and LNs were washed by centrifugation at 400×g for 5 min at 4°C unless otherwise stated. After washing, the cell suspensions were incubated with PBS containing 0.1% fixable viability dye (Thermo Fisher Scientific) for 20min. Following an additional washing step, the cells were stained with fluorochrome-conjugated antibodies specific for cell surface molecules (Supporting Information S1: Table 6) in PBS with 2% FBS (FACS buffer I) for 30 min at 4 °C. Alternatively, the viability stain was added directly to the surface staining mix. . After washing twice with FACS buffer I, samples were analyzed fresh or fixed and stored at 4 °C for up to 7 days.

For intracellular staining, surface-stained cells were fixed and permeabilized for 30 min at room temperature using Foxp3/Transcription factor fixation/permeabilization buffer (Thermo Fisher Scientific). After fixation, all following centrifugations were performed at 500×g for 5 min. Antibodies to detect intracellular proteins (Supporting Information S1: Table 6) were applied in 1x permeabilization buffer (Thermo Fisher Scientific) for 30 min at room temperature, followed by washing with 1x permeabilization buffer and immediate analysis in FACS Buffer I.

CountBright Absolute Counting Beads (Thermo Fisher Scientific) were added directly before data acquisition. Based on the ratio of cells of interest to counting beads, absolute cell numbers in spleen were calculated according to the following formula:

$$\text{absolute cell count of interest [cells/spleen]} = \frac{\text{freq of cell population [\% out of live]} \times \text{ total live cell count [cells/spleen]}}{100}$$

For staining of cells in whole blood, 25 µl PB or 50 µl cardiac puncture blood samples were stained with antibodies targeting cell surface markers (Supporting Information S1: Table 6) for 30 min at 4 °C. Afterwards, cells were fixed, and erythrocytes were lysed by addition of 2 mL Fix/Lyse Solution (eBioscience) for 10 min. After washing in FACS buffer I, cells were resuspended in FACS buffer I and stored at 4 °C. CountBright Absolute Counting Beads (Thermo Fisher Scientific) were added directly before the flow cytometry measurement. Based on the ratio of malignant cells to counting beads, absolute cell numbers in blood were calculated according to the following formula:

$$\text{absolute CLL cell counts [cells/μL]} = \frac{\text{CLL cell count [cells]} \times \text{bead volume [μL]} \times \text{bead concentration [beads/μL]}}{\text{bead count [beads]} \times \text{stained blood volume [μL]}}$$

Representative gating strategies for analysis of blood, spleen, BM and LNs are shown in Supporting Information S3: Figure 2+3.

Spectral Flow Cytometry

For spectral flow cytometry analysis of the T and NK cell compartment, B-cell depleted single cell suspensions (1×10^6 cells) were washed by centrifugation at 400×g for 5 min at 4°C unless otherwise stated. First, the cell suspensions were washed twice with PBS and stained with LIVE/DEAD Fixable Blue Dead Cell Stain Kit (Thermo Fisher Scientific) for 30 min at 4 °C. After washing with PBS containing 2 % BSA (FACS buffer II), cells were blocked for 15 min at 4 °C with mouse serum (Thermo Fisher Scientific), rat serum (Thermo Fisher Scientific), Super Bright Complete Staining Buffer (eBioscience), and CellBlox Plus Blocking Buffer (Thermo Fisher Scientific) in FACS buffer II. Subsequently, cells were directly stained with antibodies against cell surface proteins (Supporting Information S1: Table 7, 8) in FACS buffer II for 30 min at 4 °C. After washing twice with FACS buffer II, surface-stained cells were fixed for 1h at 4 °C with Foxp3/Transcription factor fixation/permeabilization buffer (Thermo Fisher Scientific). After fixation, all following centrifugations were performed at 500×g for 5 min at 4°C. Cells were washed twice with 1x permeabilization buffer (Thermo Fisher Scientific), and intracellular staining with antibodies against intracellular proteins was performed in 1x permeabilization buffer for 45 min at 4 °C. Afterwards, cells were washed twice with 1x permeabilization buffer, resuspended in FACS buffer II and stored at 4 °C in dark conditions until data acquisition on the same day.

For the spectral flow cytometry analysis of myeloid cells, 3×10^6 splenocytes were washed with PBS and stained with Zombie NIR Fixable Viability Kit (Biolegend) for 30 min at 4 °C. All centrifugation steps were performed at 400×g for 5 min at 4 °C. After washing with Cell Staining Buffer (Biolegend), cells were blocked with rat anti-mouse FcγRII/FcγRIII monoclonal Ab (2.4G2) for 10 min at 4 °C. Next, cells were directly stained with antibodies against surface proteins (Supporting Information S1: Table 7, 8) in Cell Staining Buffer (Biolegend) for 1 h at 4 °C. After washing with Cell Staining Buffer (Biolegend), cells were fixed with True-Nuclear Transcription Factor Fixation Buffer (Biolegend) overnight at 4 °C. Cells were washed with the True-Nuclear Transcription Factor Permeabilization Buffer (Biolegend) and stained with antibodies targeting intracellular proteins in True-Nuclear Transcription Factor Permeabilization Buffer (Biolegend) for

30 min at RT in the dark. After washing with True-Nuclear Transcription Factor Permeabilization Buffer (Biolegend), cells were directly acquired.

Data Acquisition and Analysis

Conventional flow cytometry was performed on BD LSRFortessa™ or FACSCanto II instruments. Compensation was performed using single-color controls, and gating was validated using fluorescence-minus-one (FMO) controls. Data were analyzed with FlowJo X v10.

Spectral flow cytometry was performed on 5-laser Cytek Aurora (UV 355 nm, violet 405 nm, blue 488 nm, yellow-green 561 nm, red 640 nm) using SpectroFlo QC beads for daily instrument standardization. The raw spectral data were unmixed with SpectroFlo v3.3.0 (Cytek Bioscience) using cell- or bead-based single-stain controls and unstained cells for autofluorescence subtraction (Supporting Information S1: Table 7, 8). Afterwards, unmixed fcs files were imported into Cytolution software (Cytolytics GmbH) or OMIQ (app.omiq.ai, Dotmatics) for data analysis.

Initial data preprocessing included transformation, compensation, time channel analysis and removal of cell debris, doublets and dead cells. Next, Uniform Manifold Approximation and Projection (UMAP) dimensionality reduction, Phenograph clustering, hierarchical classification, and manual gating were conducted. The workflow, pre-gating, and the population tree applied for the analysis of T and NK cells are shown in Supporting Information S3: Figure 4. The workflow and pre-gating for the analysis of myeloid cells are shown in the Supporting Information S3: Figure 12.

Detailed description of spectral flow cytometry data analysis

The spectral flow cytometry data-analysis workflow for T and NK cells is shown in Supporting Information S3: Fig. 4A. First, acquisition-level quality control (QC) metrics are used to detect technical artifacts introduced during data collection, including assessment of flow rate stability and per-channel signal intensity distributions, which enable identification of irregular acquisition behavior and signal dropouts. Subsequently, signal intensities for fluorescent parameters were transformed on a per-channel basis using an inverse hyperbolic sine (asinh) transformation $\mathrm{ft(x)} = \mathrm{asinh}((\mathrm{x} - \mathrm{Poffset})/\mathrm{Pwidth})$, while scatter parameters were scaled linearly. Afterwards, single, viable NK and T cells were identified based on the expression of CD45, CD11b, NK1.1, CD3, CD5, TCRβ, CD4, and CD8 (Supporting Information S3: Figure 4B). Subsequently, dimension reduction was performed using Uniform Manifold Approximation and Projection (UMAP). Afterwards, Phenograph clustering was conducted to identify different cell types (cluster resolution = high, clustering version = 2.2). In addition, fine-clustering was conducted to enhance

resolution of clusters of interest, enabling a more granular separation of phenotypically distinct subpopulations. The clusters were annotated using cell type definitions derived from a hierarchical population tree (Supporting Information S3: Fig 4C). To generate this hierarchical population tree, marker-specific cutoff values were defined, generating intervals with negative / low / high marker expression that serve as conditional rules. Then, conditions of different markers were combined using Boolean operators (AND, OR, NOT) to generate the population tree based on a hierarchical gating logic. Of note, this annotation procedure categorizes clusters post hoc without modifying the underlying clustering result, thereby preserving the unsupervised nature of the clustering. To validate cluster identities, manual gating was performed, showing the robustness of the unsupervised data analysis (Supporting Information S3: Figure 5). In addition, data quality and reproducibility were further confirmed by consistent signal distributions across groups and individual samples (Supporting Information S3: Figure 6).

The workflow for myeloid cell analysis is summarized in Supporting Information S3: Figure 12A. First, scaling and compensation were performed using NxN plots (not shown), followed by subsampling to $1x10^6$ single viable $CD45^+$ cells/sample (Supporting Information S3: Figure 12B). For cleaning, Peak Extraction and Cleaning Oriented Quality Control (PeacoQC; Maximum Allowed Median Absolute Deviations/MAD = 6; Isolation Tree Gain Limit = 0.6; Minimum Number of Consecutive Bins = 5; all fluorescent channels and autofluorescence) was run. After additional manual cleaning (Supporting Information S3: Figure 12C), the data were subsampled to $8×10^5$ cells/group. To visualize different cell types within the $CD45^+$ population, dimensionality reduction analysis was conducted using UMAP with the following parameters: all files used, lineage fluorescent parameters (CD45, CD19/Ter119/NK1.1, CD3, CD4, CD8, CD88, Ly6C, CD115, Ly6G, Fcεr1α, PDCA-1, Siglec-H, CD11c, CD26, MHC II, XCR1, IRF8, SIRPα, IRF4, CD64, PD-L1, ICOS-L, Neighbors = 15, Minimum Distance = 0.4, Components = 2, Metric = Euclidean, Learning Rate = 1, Epochs = 200, Random Seed = 9744, Embedding Initialization = spectral. After gating on the myeloid cell cluster (Supporting Information S3: Figure 12D), a second dimensionality reduction was performed using UMAP with the following parameters: all files used, fluorescent parameters CD88, Ly6C, CD115, Ly6G, Fcεr1α, PDCA-1, Siglec-H, CD11c, CD26, MHC II, XCR1, IRF8, CD8, SIRPα, IRF4, CD64, CD40, CD80, CD86, CCR7, PD-L1, ICOS-L, ICAM-1, CD44, Ki67, Neighbors = 50, Minimum Distance = 0.55, Components = 2, Metric = Euclidean, Learning Rate = 1, Epochs = 200, Random Seed = 8223, Embedding Initialization = spectral. Subsequently, PhenoGraph clustering was performed to cluster the data with the following settings: all files used, clustering features CD88, Ly6C, CD115, Ly6G, Fcεr1α, PDCA-1, Siglec-H, CD11c, CD26, MHC II, IRF8, SIRPα, CD64, CD40, CD80, CD86, CCR7, PD-L1, ICOS-

L, ICAM-1, Ki67, umap_1, umap_2, K Nearest Neighbors = 20, Nearest Neighbors algorithm = Annoy, Distance Metric = euclidian, Clustering method = Leiden, Random Seed = 1722. Cluster identities were validated by parallel manual gating, demonstrating strong concordance between unsupervised data analysis and manual gating (Supporting Information S3: Figure 13). Data quality and reproducibility were further confirmed by consistent signal distributions across groups and individual samples (Supporting Information S3: Figure 14).

Statistical analysis

Data were analyzed using GraphPad Prism V9.5.1 and R programming language in the R studio interface.[1,2] To compare more than 2 groups, one-way analysis of variance (ANOVA) was used followed by Dunnett's multiple comparison test. For pairwise comparison of the immune cell populations in blood within the same mice before and after AT, paired two-tailed Student's t-tests were performed with Bonferroni-Holm correction for multiple testing. In all analyses, values of $p \leq 0.05$ were considered statistically significant. Boxplots show the 25th and 75th percentiles around the median with whiskers extending to the most extreme data point which is no more than 1.5 × the interquartile range.

Cell Population Correlation Analysis

Spectral flow cytometry data from the longitudinal TCL1 mouse study were analyzed for T/NK and myeloid cell populations. Time points were standardized by converting PBS control samples to time point 0 and extracting numeric week values. Cell population frequencies were calculated relative to T/NK or myeloid cells. Spearman rank correlations were computed across all time points combined using the corr.test function (psych package v2.6.1) after combining cell populations frequencies for T/NK and myeloid cells (n=20 mice with complete data). All p-values were adjusted for multiple comparisons using the Benjamini-Hochberg method. Significant correlations were defined as adjusted $p < 0.05$. Correlation matrices were visualized as heatmaps with hierarchical clustering using corrplot. Significant correlations ($p < 0.05$) were marked with asterisks. All analyses were performed in R (v4.5.2) using tidyverse (2.0.0), readxl (1.4.5), psych, and corrplot (0.95) packages.

References

1. R Core Team. R: A Language and Environment for Statistical Computing. R Foundation for Statistical Computing, Vienna. . https://www.R-project.org; 2025.
2. Posit team. RStudio: Integrated Development. Environment for R. Posit Software, PBC, Boston, MA. http://www.posit.co/. 2026.
